# Michael Addition–Elimination Ring-Opening Polymerization

**DOI:** 10.1021/jacs.4c05054

**Published:** 2024-06-21

**Authors:** Yong-Liang Su, Wei Xiong, Liang Yue, Mckinley K. Paul, Kaitlyn S. Otte, John Bacsa, H. Jerry Qi, Will R. Gutekunst

**Affiliations:** † School of Chemistry and Biochemistry, Georgia Institute of Technology, Atlanta, Georgia 30332, United States; ‡ School of Mechanical Engineering, Georgia Institute of Technology, Atlanta, Georgia 30332, United States; § Department of Chemistry, Emory University, Atlanta, Georgia 30322, United States

## Abstract

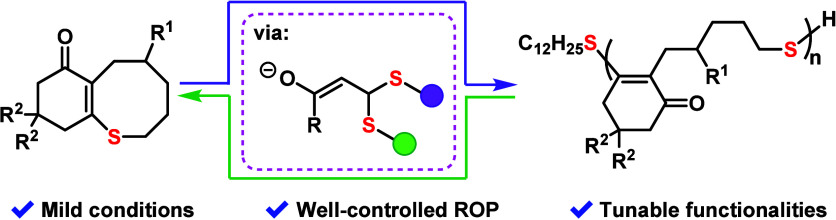

A cyclic thioenone system capable of controlled ring-opening polymerization (ROP) is presented that leverages a reversible Michael addition–elimination (MAE) mechanism. The cyclic thioenone monomers are easy to access and modify and for the first time incorporate the dynamic reversibility of MAE with chain-growth polymerization. This strategy features mild polymerization conditions, tunable functionalities, controlled molecular weights (*M*_n_), and narrow dispersities. The obtained polythioenones exhibit excellent optical transparency and good mechanical properties and can be depolymerized to recover the original monomers. Density functional theory (DFT) calculations of model reactions offer insights into the role of monomer conformation in the polymerization process, as well as explaining divergent reactivity observed in seven-membered thiepane (**TP**) and eight-membered thiocane (**TC**) ring systems. Collectively, these findings demonstrate the feasibility of MAE mechanisms in ring-opening polymerization and provide important guidelines toward future monomer designs.

## Introduction

Ring-opening polymerization (ROP) is recognized for its versatility in creating polymers with a range of architectures and functionalities.^1^ This method’s adaptability extends to reversible depolymerization systems, where a careful balance of polymerization thermodynamics can lead to the recovery of original monomers.^2^ The growing focus on recyclable polymers has led to significant advances using ROP, and several platforms including (thio)lactones,^3^ cyclic carbonates,^4^ cyclic acetals,^5^ cyclooctenes,^6^ and others^7^ have been developed. Ring strain energy, quantified as the enthalpy (Δ*H*), is a fundamental parameter that influences both polymerization and depolymerization processes. Conventional wisdom suggests that modest ring strain is advantageous when designing recyclable monomers, so that the monomer equilibrium can be overcome by entropy (Δ*S*) at the appropriate temperature or concentration.^2g,6^

**Figure 1:**
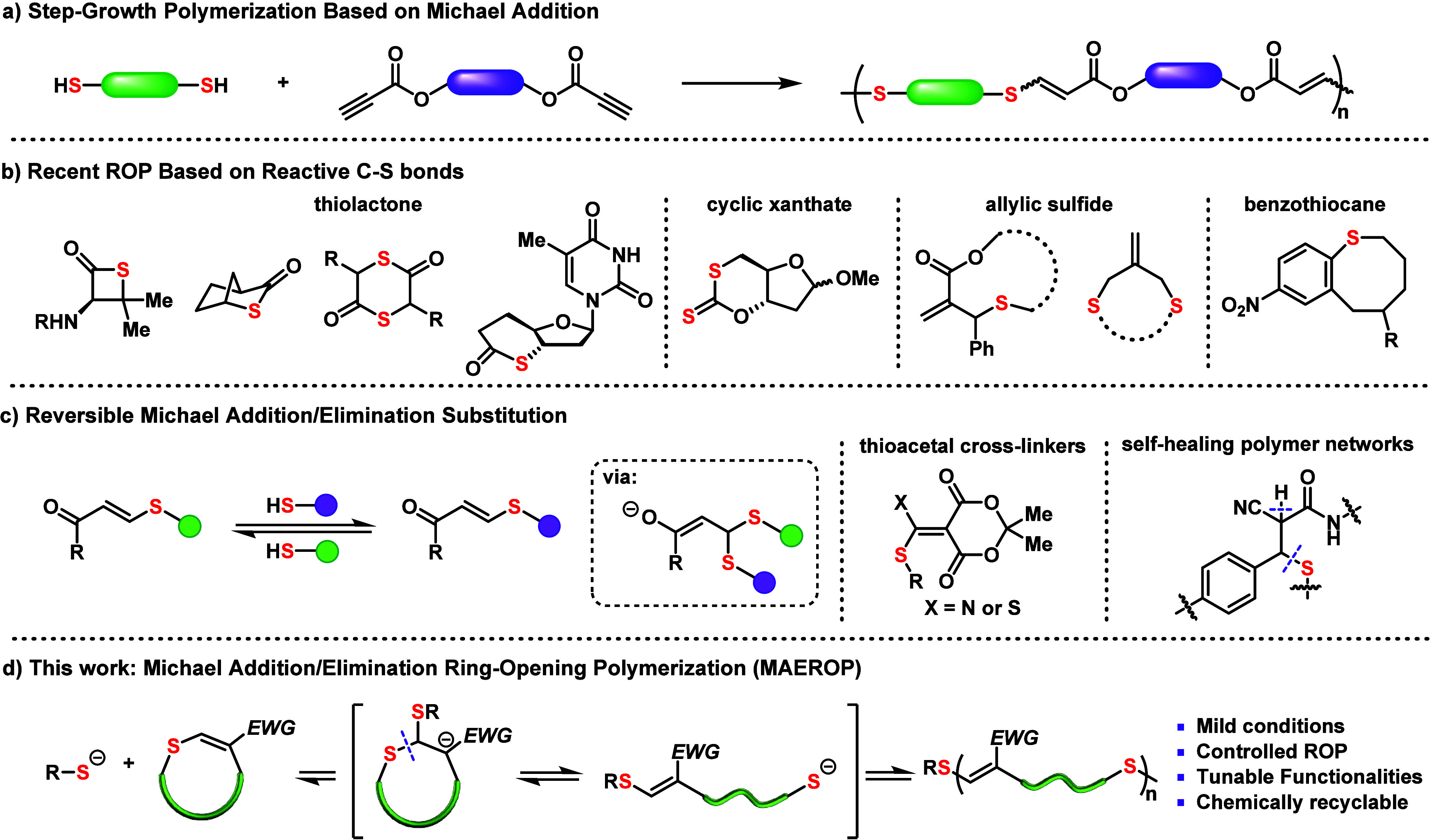
Designs of Michael addition/elimination ring-opening polymerization (MAEROP). (a) Conventional stepwise polymerization via the Michael addition mechanism. (b) Established cyclic monomer scaffolds for preparing sulfur-containing polymers. (c) Overview of reversible Michael addition/elimination reaction and its application in cross-linked polymer network synthesis. (d) MAEROP for the synthesis of chemically recyclable polythioenones.

Sulfur-containing polymers show distinctive properties and characteristics, such as high chemical resistance, thermal stability, and flame retardancy, which make them suitable for specific industrial and technological applications.^8^ Recently, several classes of sulfur-containing monomers for applications of ROP have been developed based on the cleavable C–S bonds,^9^ such as thiolactones,^3d,3h,10^ cyclic xanthates,^11^ cyclic allyl sulfides,^12^ and benzothiocanes^13^ (Figure 1b). Specifically, the groups of Lu and Chen introduced carefully designed thiolactone platforms for recyclable polymers through the utilization of the bridged-ring or geminal dimethyl effect in ROP.^3d,3e^ Despite these advances, the range of monomers for preparing recyclable sulfur-containing polymers remains limited. There is a growing need to diversify the chemical toolbox for producing chemically recyclable polymers, particularly for systems that strike a balance between (de)polymerizability and material performance.

Dynamic covalent chemistry (DCC) is characterized by reaction systems that permit the rapid generation and cleavage of chemical bonds under thermodynamic equilibria. It has been exploited for the synthesis of highly ordered and self-correcting materials in both two- (2D) and three-dimensional (3D) scenarios.^14^ These reversible reactions also provide new avenues for designing dynamic 1D polymeric materials through ROP. The Michael addition reaction of thiols and electron-deficient alkynes has been commonly employed in step-growth polymerization and feature reversibility through thioether exchange (Figure 1a).^15^ The dynamic behavior of Michael addition–elimination (MAE) of the resulting thioenol ether adducts has also been investigated in bioconjugation chemistry^16^ and the preparation of self-healing polymer networks.^17^ Anslyn and co-workers reported a reversible exchange reaction of Meldrum’s acid alkylidene derivatives and thiols for application to peptide conjugation (Figure 1c).^16b^ Afterward, Kalow and co-workers developed a Meldrum’s acid-derived cross-linker to prepare silicone vitrimers.^17a^ Rosales and Anslyn further studied the effect of pH on the properties of hydrogels cross-linked via dynamic thia-Michael addition bonds.^17b^

Despite these advances, these strategies are limited to step-growth polymerization, and chain-growth polymerization utilizing Michael addition–elimination is underexplored. Huang and co-workers reported the ROP of macrocyclic allylic sulfides; however this mechanism involves double-bond transposition and lacks reversibility due to the stability of the ring-opened product.^12c^ To merge the dynamic behavior of Michael addition–elimination reaction with reversible chain-growth polymerization, an electron-deficient cyclic β-thioenone was designed and polymerized via Michael addition–elimination ring-opening polymerization (MAEROP) for the first time (Figure 1d).

## Results and Discussion

### Monomer Design and Synthesis

To initiate studies on MAEROP, seven- and eight-membered ring systems were targeted, as they were anticipated to provide modest ring-strain energies for polymerization. Two fused bicyclic ring systems, seven-membered thiepane (**TP**) and eight-membered thiocane (**TC**), were designed for initial ROP experiments. The syntheses of both **TP** and **TC** structures are rapidly assembled via the [3,3]-sigmatropic rearrangement of alkynyl sulfoxides reported by Zhang and co-workers.^18^ This reaction directly gives bicyclic monomers in which the dialkyl ketone can be chemoselectively transformed into other functional groups. For example, the original ketone (**TC-C=O**) could be easily converted into an exocyclic C=C double bond (**TC-C=C**), through the Wittig reaction. Alternatively, it could be reduced to an alcohol that was amenable to further modification to introduce side-chain functionalities, including **TC-OAc** and **TC-OBz**. The monomers were fully characterized with ^1^H NMR and ^13^C NMR spectrometry and mass spectrometry. Furthermore, single-crystal X-ray analysis revealed differences in the ground state conformations of these compounds. Specifically, **TC-C=O** features an eight-membered ring in twist boat-chair conformation, while **TC-OAc** exhibits a boat–boat conformation.^19^ The twist boat–chair conformation has an exposed enolsulfide that facilitates nucleophilic attack from the top face, while the boat–boat conformation has substituents hindering nucleophilic attack from both the top and bottom faces (see Supplementary Figure S15 for details). These findings lend insight into the subtle role of conformation on the ring-opening behavior of these monomers (vide infra).

### Polymerization Studies

Initial tests to assess the polymerizability of seven-membered **TP-C=O** and eight-membered **TC-C=O** were performed using C_12_H_25_SH as the initiator and 1,8-diazabicyclo[5.4.0]-undec-7-ene (DBU) as the base. Interestingly, **TP-C=O** did not polymerize and instead underwent ring-opening through an S_N_2 pathway upon reaction with benzyl thiolate (Figure 3a). After initial substitution, the resulting thioenolate was found to ring close into a stable thio-hemiacetal upon quenching with acid, which was confirmed through ^1^H NMR and mass spectrometry (see Supplementary Figure S7 for studies). In contrast, **TC-C=O** exhibited successful polymerization and resulted in the formation of polymer **PTC-C=O**. The selection of the solvent was a crucial factor in the polymerization process. It was noted that in polar aprotic solvents such as dimethyl sulfoxide (DMSO), dimethylformamide (DMF), and acetonitrile (CH_3_CN), rapid reaction rates and high conversions were commonly attained (Supplementary Table S1). However, relatively high dispersities were observed, which was attributed to rapid chain transfer of the dynamic linkages. In contrast, relatively narrow distributions were achieved in less polar solvents, such as tetrahydrofuran (THF) and 1,4-dioxane, and mixtures of these solvents were found to balance solubility of the polymer product and reactivity. Further exploration of different bases and thiols revealed that while appropriate basicity is essential to provide the anionic thiolate initiator, excessively strong bases would lead to broader dispersities (Supplementary Table S3 and Table S4). By variation of the monomer to initiator ratios, a series of **PTC-C=O** with targetable molecular weights was obtained with low dispersities (Table 1, entries 2–6). Good control was maintained up to degrees of polymerization of 100 (DP 100), while some reduction in conversion and broadening of dispersity were found with high molecular weight targets (Figure 4a). This is possibly due to chain-end deactivation during extended reaction times or solubility issues. Kinetic studies were performed by sampling and terminating the polymerization at different time points. The linear correlation of the *M*_n_ of **PTC-C=O** as a function of monomer conversion and the unimodal and narrow molecular weight distribution (*Đ* < 1.2) over 90% conversion are consistent with living characteristics (Figure 4b). Additional analysis showed clear first-order kinetic behavior with respect to that of **TC-C=O** (Figure 4c). Upon termination of a **PTC-C=O** polymerization (DP 25) with iodoacetamide, the end-functionalized polymer was analyzed by matrix-assisted laser desorption/ionization-time-of-flight (MALDI-TOF) mass spectrometry (Figure 4d). While major peaks correspond to sodium adducts, protonated ions and potassium adducts can also be readily identified. Notably, the consistent spacing of 238.3 Da between major peaks correlates to the molar mass of the **TC-C=O** repeat unit. Furthermore, all ions exclusively feature (C_12_H_25_S–/–CH_2_CONH_2_) end groups, affirming the fidelity of the chain ends and the high efficiency of the end-capping process.

**Figure 2:**
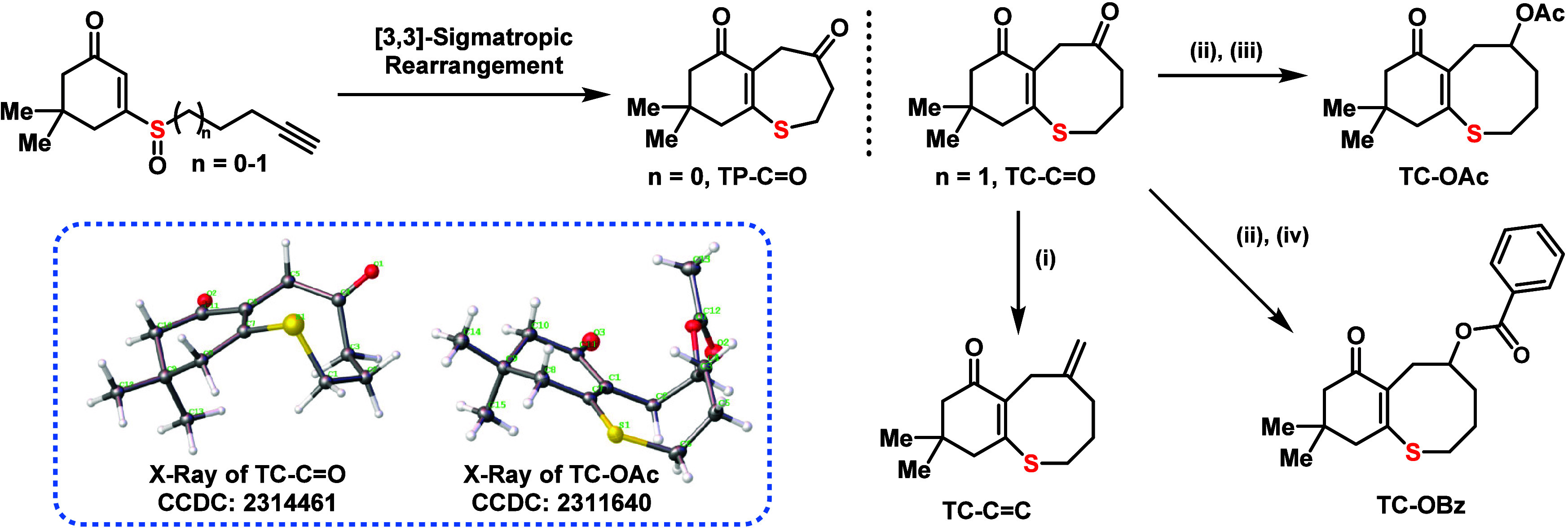
Design, synthesis, and characterization of the monomers. [3,3]-Sigmatropic rearrangement of alkynyl sulfoxides provides fused bicyclic thiocane. (i) ^*t*^BuOK, PPh_3_PMeBr, THF. (ii) NaBH_4_, MeOH, 0 °C. (iii) Ac_2_O, DMAP, pyridine. (iv) BzCl, DMAP, Et_3_N, DCM. The molecular structures of **TC-C=O** (left) and **TC-OAc** (right) as determined by SC-XRD are shown as the inset.

**Figure 3:**
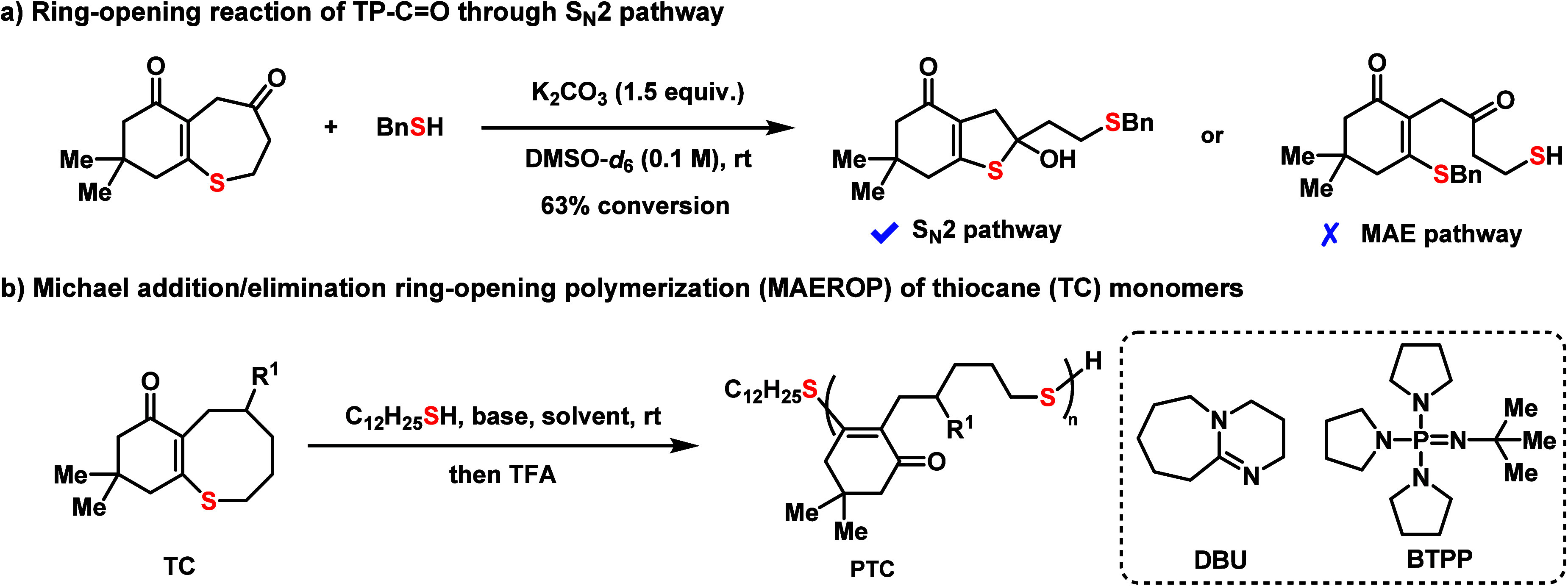
Ring-opening reaction of TP-C=O and MAEROP of TC monomers.

**Table 1:** MAEROP Results for Thiepane and Thiocane Monomers^a^

entry	monomer	base	cond.^b^	[**TC**]_0_:[I]_0_:[base]_0_	time (h)	conv.^c^	*M*_n,SEC_ (kDa)^d^	*Đ*^d^	*T*_d,5%_ (°C)^e^	*T*_g_ (°C)^f^
1	TP-C=O	DBU	A	50:1:1	3	<5%	-	-	-	-
2	TC-C=O	DBU	A	25:1:1	1.5	94%	8.4	1.22	-	-
3	TC-C=O	DBU	A	50:1:1	7	92%	18.8	1.20	328	34
4	TC-C=O	DBU	A	75:1:1	15	88%	27.3	1.34	-	-
5	TC-C=O	DBU	A	100:1:1	24	87%	34.4	1.43	-	-
6	TC-C=O	DBU	A	150:1:1	46	67%	38.5	1.48	-	-
7	TC-C=C	DBU	B	50:1:1	52	71%	17.4	1.34	309	20
8	TC-OAc	DBU	B	25:1:1	5	90%	12.2	1.28	-	-
9	TC-OAc	DBU	B	50:1:1	15	85%	20.1	1.35	263	51
10	TC-OBz	DBU	C	50:1:1	52	82%	22.3	1.40	234	58
11	TC-C=O/TC-OAc	DBU	A/B	25:25:1:1	1.5/16	>99%/70%	22.8	1.65	-	-
12^g^	TC-OAc	BTPP	B	150:1:1	20	96%	92.8	1.50	-	-

a Polymerizations were performed under a N_2_ atmosphere with C_12_H_25_SH as the initiator.

b Conditions: A: THF/1,4-dioxane (1/1), [**TC**]_0_ = 1.5 M; B: DMF, [**TC**]_0_ = 2.5 M; C: DMF, [**TC**]_0_ = 2.0 M.

c The monomer conversion, determined by ^1^H NMR spectroscopy in CDCl_3_.

d Determined by CHCl_3_ size-exclusion chromatography (SEC) calibrated using polystyrene standards.

e The temperature causing a 5% weight loss, measured by thermogravimetric analysis (TGA).

f The glass transition temperature, measured by differential scanning calorimetry (DSC).

g 2.4 mmol of **TC-OAc**.

**Figure 4:**
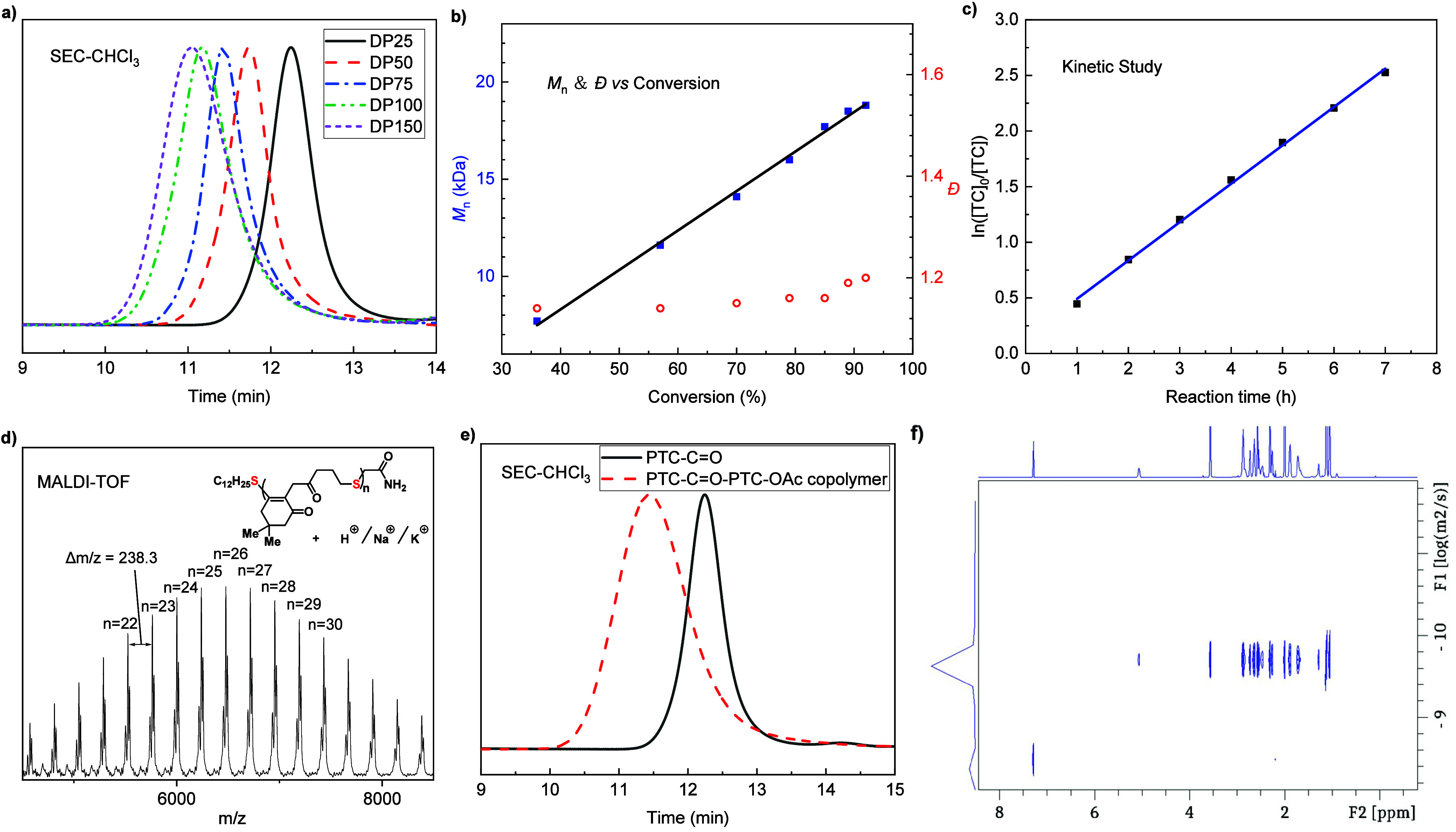
Controlled MAEROP of **TC-C=O** and characterization of **PTC-C=O**. (a) SEC curves for **PTC-C=O** produced at different [**TC-C=O**]_0_:[DBU]_0_:[C_12_H_25_SH]_0_ ratios. (b) *M*_n_–conversion correlation (blue) and *Đ*–conversion correlation (red) of MAEROP of **TC-C=O**. (c) First-order kinetic plot for the polymerization of **TC-C=O** targeting DP 50. (d) MALDI-TOF mass spectrum of **PTC-C=O**, terminal thiol capped by iodoacetamide. (e) Overlay of SEC curves of the precursor polymer **PTC-C=O** and the **PTC-C=O**-**PTC-OAc** copolymer. (f) DOSY NMR spectrum.

Next, the scope of the method was explored, and relatively low reactivities were found when modifying the C=O group to either C=C (**TC-C=C**) or ester groups (**TC-OAc** and **TC-OBz**). Therefore, the polar solvent DMF was used to increase conversions (Table 1, entries 7–10). It was found that the polymerization rate follows **TC-C=O** > **TC-OAc > TC-C=C**, which can be explained by the different steric environments of the enolsulfides in each monomer. X-ray crystallography and DFT (vide infra) studies support a twist boat–chair ground state conformation for **TC-C=O** and boat–boat ground state conformations for **TC-OAc** and **TC-C=C** (see Supplementary Figure S15 for details). The more accessible enolsulfide in **TC-C=O** allows for facile nucleophilic attack by the thiolate anion, whereas **TC-OAc** and **TC-C=C** need to undergo a conformational change to ring-open. The high fidelity of thiol end-groups after polymerization was further confirmed by the copolymerization of **TC-C=O** with **TC-OAc** (Table 1, entry 11). A copolymer was obtained by the sequential addition of **TC-C=O** and **TC-OAc**, as demonstrated by the SEC overlay (Figure 4e) and DOSY NMR characterizations (Figure 4f). A stronger phosphazene base *tert*-butylimino-tri(pyrrolidino)phosphorane (BTPP) was used for the polymerization of **TC-OAc** targeting DP150, yielding the desired **PTC-OAc** with a molecular weight of 92.8 kDa at a gram scale (Table 1, entry 12). The flexibility in altering the scaffolds and the wide range of decorations enable fine-tuning of the properties of the resulting material, as illustrated below.

### Proposed Mechanism

**Figure 5:**
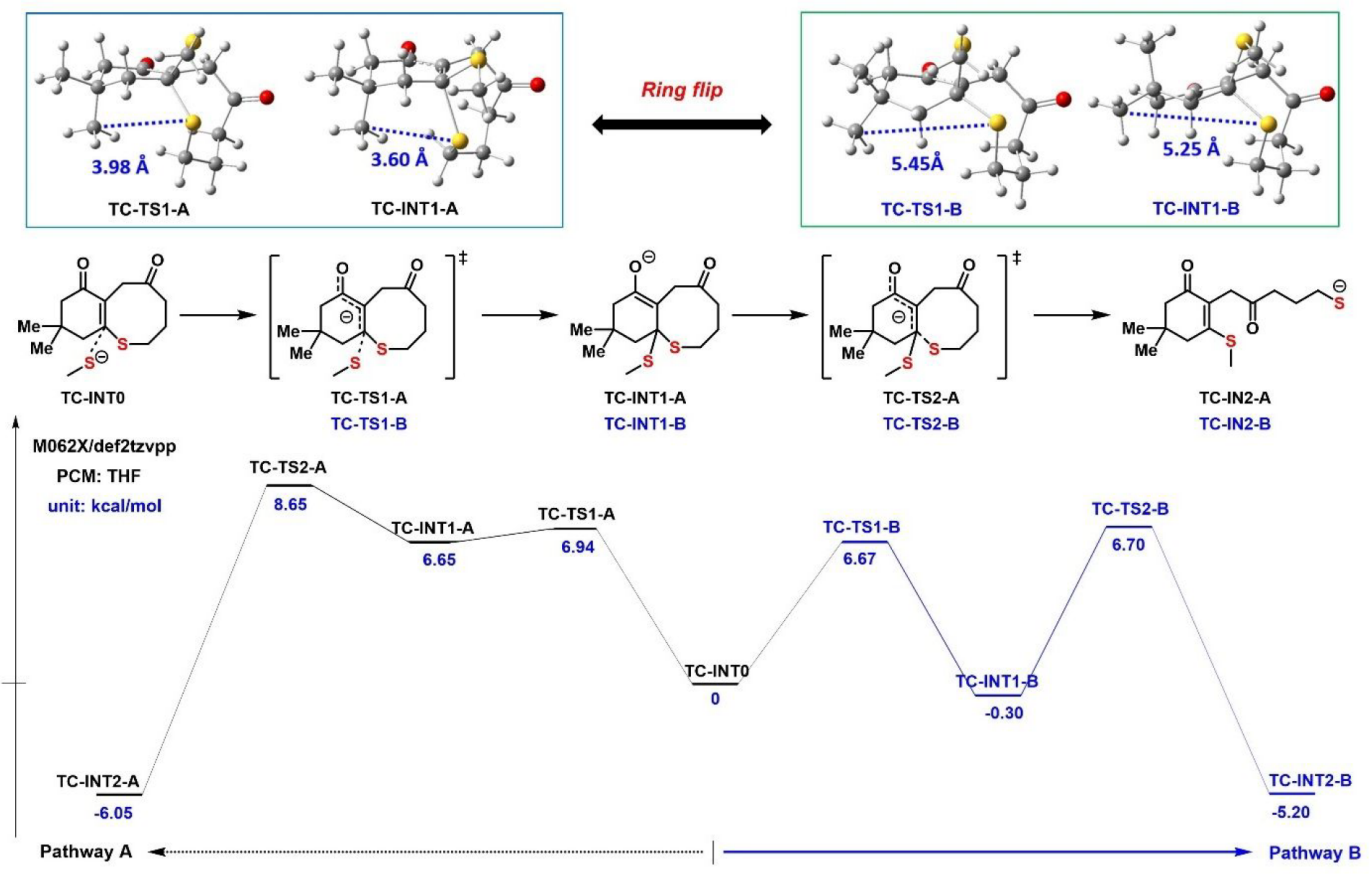
Free-energy profiles for the ring-opening process of **TC-C=O** initiated by the active chain end. PCM: Polarizable continuum model.

To gain insight into the differences in products between **TP-C=O** and **TC-C=O**, as well as changes in reactivity in this series, the free energies of intermediates (**INT**) and transition states (**TS**) in the ring-opening process were calculated with density functional theory (DFT). To simplify the calculation, the chain end was modeled as a dissociated anionic methyl thiolate as the initiator. The free energies of key intermediates and transition states in the chain propagation are summarized in Figure 5. According to the calculation, there are two viable pathways (A and B) for the ring-opening process of **TC-C=O**. Both of these pathways are in agreement with the standard MAE mechanism and differ in the conformations of the bicyclic intermediates. In pathway A, the ring-opening process consists of two elementary steps, nucleophilic attack and ring-opening, with transition states **TC-TS1-A** and **TC-TS2-A** with free energies of 6.94 and 8.65 kcal/mol, respectively. In the first stage, the in-ring C–S bond rotates from equatorial in **TC-INT0** to axial position in **TC-INT1-A**, which will facilitate the further dissociation of thiolate anion via β-elimination. However, as shown in Figure 5, the steric repulsion between the axial geminal dimethyl group and C–S bond leads to the high free energy of **TC-INT1-A**. In pathway B, the geminal dimethyl will experience a ring flip after being attacked by the initiator. This isomerization of the monomer reduces steric repulsion and leads to lower free energies of **TC-TS1-B** and **TC-INT1-B** (6.67 and −0.30 kcal/mol). According to the Eyring equation, the 1.95 kcal/mol difference in energy barrier of **TC-TS2-A** and **TC-TS2-B** (8.65 vs 6.70 kcal/mol) suggests that the rate constants of pathway B are ∼10-fold higher than those of pathway A. Hence, pathway B involving a conformational flip is favored, with dissociation being the rate-determining step of ROP.

Given the different ground state conformations observed between **TC-C=O** and **TC-OAc** in the solid state (Figure 2), DFT calculations were used to determine the difference in energies between the boat–boat and twist boat–chair conformations for these two monomers, as well as **TC-C=C**. The DFT calculations found **TC-C=O** favored the twist boat–chair conformation by 0.82 kcal/mol, while **TC-C-OAc** and **TC-C=C** favored the boat–boat conformation by 1.69 and 3.41 kcal/mol, respectively (Supplementary Figure S15). This is in agreement with our experimental findings that show a slower rate of polymerization for **TC-C-OAc** and **TC-C=C**, and it suggests a ring-flip is likely necessary before nucleophilic attack can occur on the enolsulfide. This would also qualitatively explain the relative rates of polymerization between **TC-C-OAc** and **TC-C=C**, as **TC-C=C** favors the boat–boat conformation more strongly.

To further investigate the effect of the dimethyl groups in this process, the monomer lacking methyl groups, **TC-CHD**, was prepared following the general procedure (see Supporting Information for details). Under conditions optimized for **TC-C=O**, it was found that full conversion was obtained in under 2 h to give an *M*_n_ of 18.7 kDa and *Đ* = 1.40 (Figure 7a). In contrast, **TC-C=O** required over 7 h to reach full conversion. In addition to demonstrating higher propagation rates compared to **TC-C=O**, **TC-CHD** exhibits a more exergonic calculated ring-opening Δ*G* of −6.78 kcal/mol, compared to −5.20 kcal/mol for **TC-C=O** (Figure 7b). The boat–boat and twist boat–chair conformations of **TC-CHD** were both calculated and found to have similar free energy. Both of these conformers possess an enol sulfide that is readily accessible for nucleophilic attack, which supports the faster polymerization rate and the overall role of the dimethyl groups in the parent **TC** series (Supplementary Figure S15).

**Figure 6:**
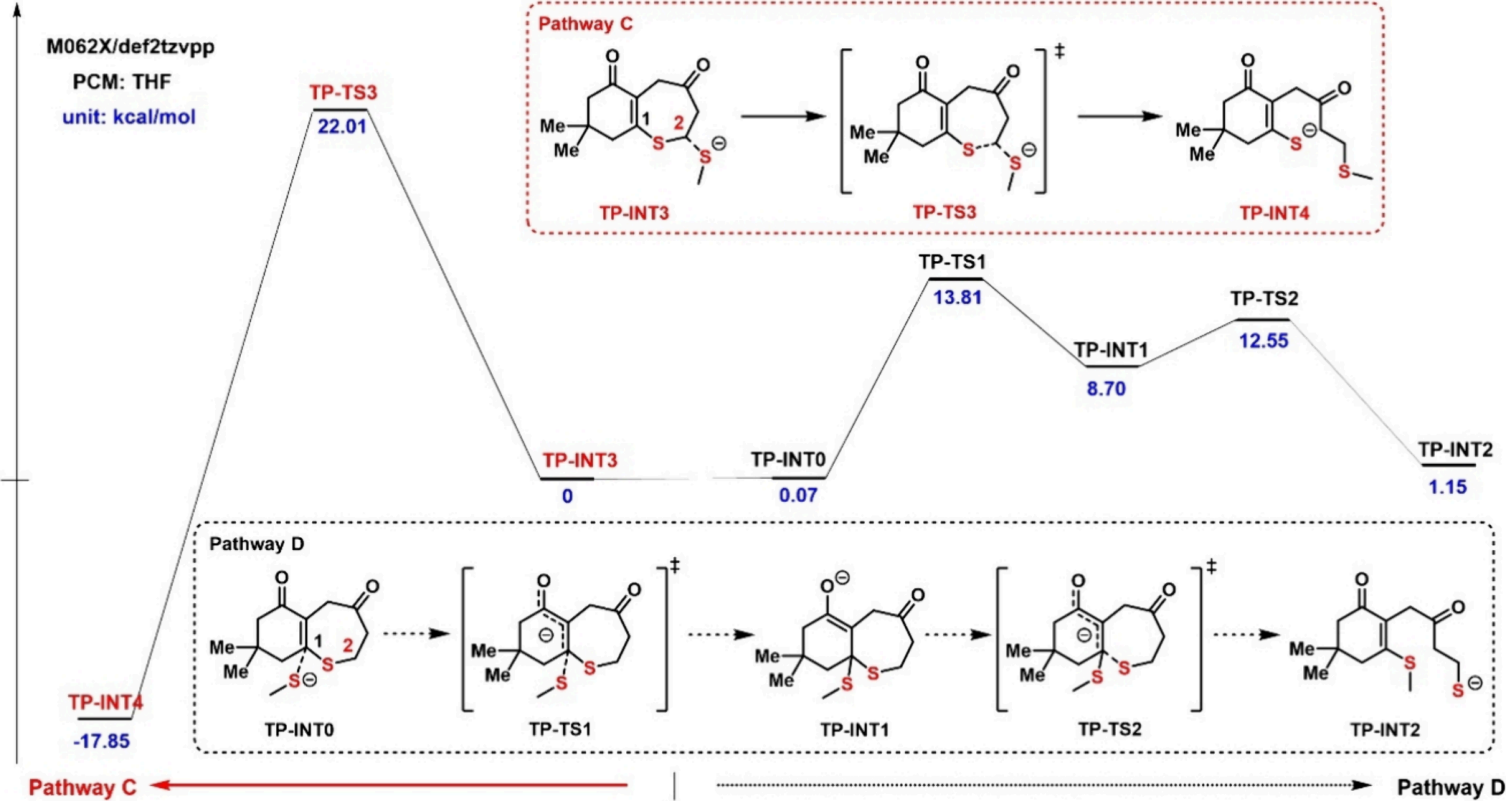
Free-energy profiles for the ring-opening process of **TP-C=O**.

**Figure 7:**
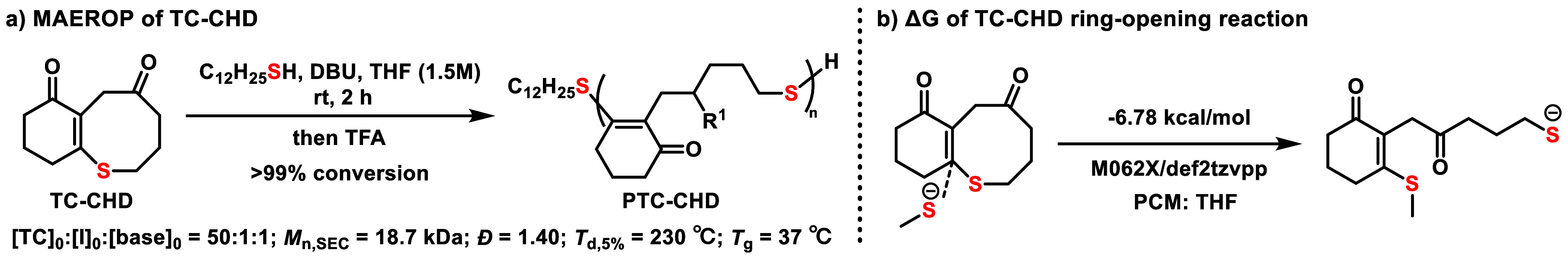
Experimental polymerization of **TC-CHD** and DFT calculations for the ring-opening of **TC-CHD**.

Although **TP-C=O** did not polymerize, it underwent an S_N_2 ring-opening reaction with benzyl thiolate. Rather than the anticipated sp^2^-C–S bond cleavage via the MAE pathway D, the cleavage occurred at the sp^3^-C–S bond through pathway C. Motivated by the unexpected results, we developed a comprehensive picture (Figure 6) of the ROP processes of **TP-C=O** at both the 1- and 2-sites. Similar to **TC-C=O**, the ring-opening reaction at the 1-site of **TP-C=O** involves two elementary steps. In **TP-C=O**, though, the nucleophilic attack was identified as the rate-determining step, with the free energy of **TP-TS1** (13.81 kcal/mol) being higher than that of **TP-TS2** (12.55 kcal/mol). The overall MAE process shows the ring-opening product (**TP-INT2**) with a slightly higher energy than the ring-closed form (**TP-INT0**), which suggests a lack of an enthalpic driving force for the reaction to occur. The S_N_2 ring-opening process, denoted as pathway C, exhibits a notably higher activation energy (22.01 kcal/mol) than pathway D (13.81 kcal/mol), indicating that pathway D is dynamically favored. However, the calculated energy differences (−17.85 kcal/mol) between **TP-INT4** and **TP-INT3** are significantly lower than that between **TP-INT2** and **TP-INT0** (1.15 kcal/mol), indicating pathway C is thermodynamically favored. While the MAE pathway is kinetically viable, the lack of ring-strain combined with the effective irreversibility of the S_N_2 pathway explains the lack of polymerization in the thiepane monomer **TP-C=O**.

### Thermal and Mechanical Properties

**Figure 8:**
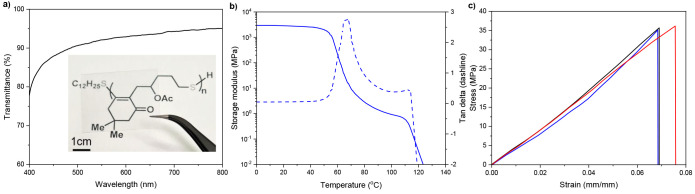
Mechanical properties of **PTC-OAc**. (a) UV–vis spectrum and the photograph of **PTC-OAc** film. (b) DMA storage modulus and tan δ profiles of **PTC-OAc** (*M*_n_: 92.8 kDa; *Đ*: 1.50). (c) Tensile stress–strain curves of **PTC-OAc** (*M*_n_: 92.8 kDa; *Đ*: 1.50).

The thermal properties of **PTC** were measured by thermogravimetric analysis (TGA) and differential scanning calorimetry (DSC). As shown in Table 1 and Figure S10, the resulting polymers presented good thermal stability with a range of thermal decomposition temperatures *T*_d_ (defined as the temperature causing a 5% weight loss) from 230 to 328 °C. Notably, a wide range of glass transition temperature (*T*_g_) from 20 to 58 °C can be achieved by altering the scaffolds and side-chain functional groups. In addition, these polymers were determined to be amorphous, as no observable melting temperature was found in DSC. In Figure 8, a comprehensive characterization of the polymer **PTC-OAc** (*M*_n_: 92.8 kDa; *Đ*: 1.50) is presented, which elucidates its potential for applications requiring transparency, flexibility, and mechanical resilience. The UV–vis spectroscopy analysis reveals a high degree of transmittance across the visible spectrum, indicating the polymer’s excellent transparency, as seen in the photograph of its film (Figure 8a). Dynamic mechanical analysis (DMA) delineates the material’s storage modulus across varying temperatures (Figure 8b). The observed rapid decrease in storage modulus signifies the glass transition region, as corroborated by the peak in the tan δ curve (*T*_g_ = 66 °C). Furthermore, the tensile tests on **PTC-OAc** align with the expected behavior of a glassy polymer, exhibiting a Young’s modulus of 466.4 ± 42.6 MPa, an ultimate tensile strength (σ) of 35.06 ± 1.35 MPa, and a strain at break (ε) of 7.2 ± 0.4% (Figure 8c). The tensile properties of this polymer are comparable to those of commonly used thermoplastics such as polyethylene (PE), ethylene vinyl acetate (EVA), and polyvinyl chloride (PVC). Collectively, these findings encapsulate **PTC-OAc**’s excellent optical transparency coupled with its promising mechanical properties, underscoring its suitability for applications where these properties are critical.

### Chemical Recyclability

**Figure 9:**
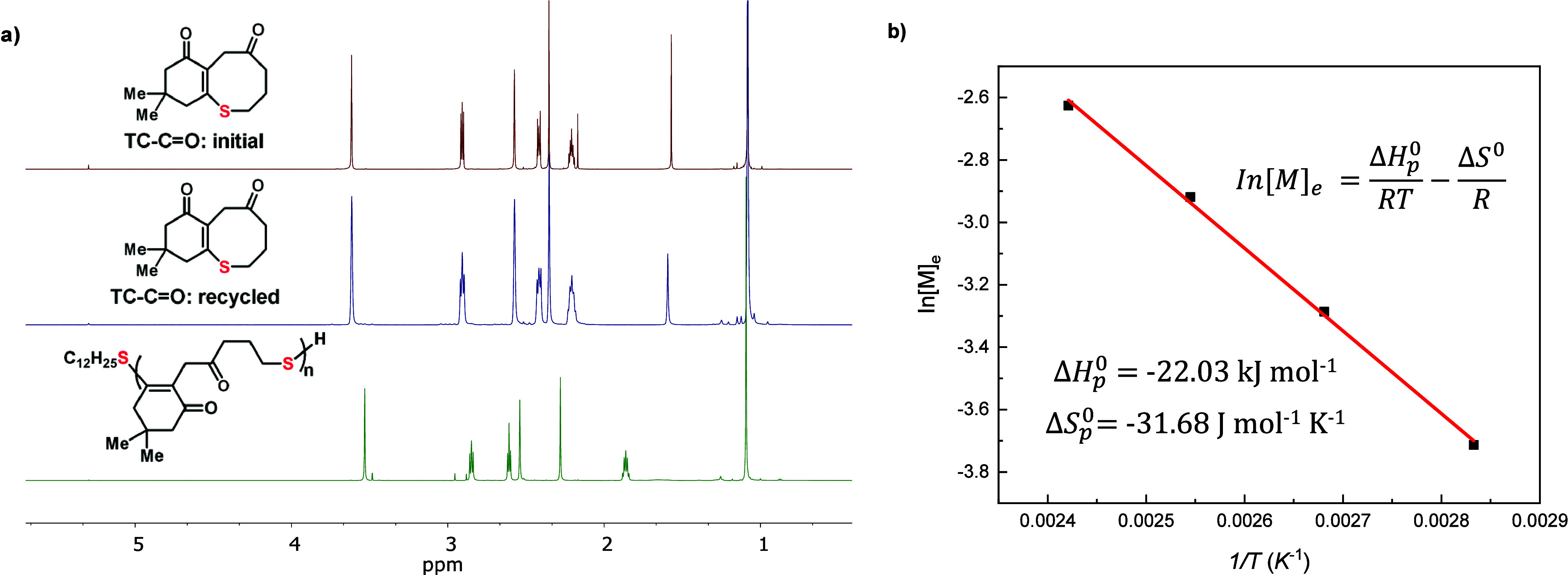
Chemical recyclability of **PTC-C=O** and the thermodynamics of **TC-C=O** polymerization. (a) Overlays of ^1^H NMR spectra of initial and recycled **TC-C=O** and **PTC-C=O** in CDCl_3_. (b) The van’t Hoff plot of **TC-C=O** ([M]_0_ = 0.20 M), the enthalpy change Δ*H*, and entropy change Δ*S* were calculated from the slope and intercept of the plot.

**PTC-C=O** was selected as a representative to test its recyclability to the original monomer. By employing DBU (0.65 equiv relative to the repeat unit) at a concentration of 10 mg/mL and a reaction temperature of 150 °C in DMF, the monomer **TC-C=O** was successfully recovered with a 63% yield (Figure 9a). Size-exclusion chromatography (SEC) analysis of the depolymerization process revealed that the degradation proceeded through a backbone cleavage mechanism (Supplementary Figure S11). Furthermore, the thermodynamic analyses of ROP of **TC-C=O** was performed (Figure 9b). The thermodynamic parameters were extracted by the linear fitting of the plot of ln[M]_e_ against 1/*T* according to the van’t Hoff equation, where the [M]_e_ is the monomer concentration at equilibrium. The changes in enthalpy (Δ*H*_*p*_^0^) and entropy (Δ*S*_*p*_^0^) were determined as follows: Δ*H*_*p*_^0^ = −22.0 kJ mol^–1^ and Δ*S*_*p*_^0^ = −31.7 J mol^–1^ K^–1^, and the ceiling temperature was then calculated from these values as 421 °C. These values provide important thermodynamic insights into the recyclability of **TC-C=O**, and more efforts are ongoing to further improve the recovery yield.

## Conclusions

In conclusion, this research introduces a new chemically recyclable system combining the reversible Michael addition–elimination reaction and ring-opening polymerization in a novel fused bicyclic ring system. The thiocane monomers (**TC**) are easy to synthesize and modify, and their polymerizations and copolymerizations can produce a variety of polythioenones with tunable material properties. The mechanism of this MAEROP was thoroughly investigated and elaborated with DFT calculations, providing fundamental physical organic insights into the process. Furthermore, thermodynamic studies were conducted to determine the changes in enthalpy (Δ*H*_*p*_^0^) and entropy (Δ*S*_*p*_^0^) of the polymerization, which also explains the moderate monomer recovery yield during the depolymerization process. Moving forward, efforts are directed toward skeletal adjustments to further improve the monomer recovery efficiency and the thermomechanical properties.

## Supporting Information Available

Experimental procedure and spectroscopic data for all new compounds (PDF)

## References

[ref1] aNuyken O.; Pask S. D. Ring-Opening Polymerization-An Introductory Review. Polymers 2013, 5, 361–403. 10.3390/polym5020361.

[ref2] aPlummer C. M.; Li L.; Chen Y. Ring-Opening Polymerization for the Goal of Chemically Recyclable Polymers. Macromolecules 2023, 56, 731–750. 10.1021/acs.macromol.2c01694. 36818576 PMC9933900

[ref3] aZhu J.-B.; Watson E. M.; Tang J.; Chen E. Y.-X. A Synthetic Polymer System with Repeatable Chemical Recyclability. Science 2018, 360, 398–403. 10.1126/science.aar5498. 29700260

[ref4] Zhang W.; Dai J.; Wu Y.-C.; Chen J.-X.; Shan S.-Y.; Cai Z.; Zhu J.-B. Highly Reactive Cyclic Carbonates with a Fused Ring toward Functionalizable and Recyclable Polycarbonates. ACS Macro Lett. 2022, 11, 173–178. 10.1021/acsmacrolett.1c00653. 35574765

[ref5] aAbel B. A.; Snyder R. L.; Coates G. W. Chemically Recyclable Thermoplastics from Reversible-Deactivation Polymerization of Cyclic Acetals. Science 2021, 373, 783–789. 10.1126/science.abh0626. 34385394

[ref6] aSathe D.; Zhou J.; Chen H.; Su H.-W.; Xie W.; Hsu T.-G.; Schrage B. R.; Smith T.; Ziegler C. J.; Wang J. Olefin Metathesis-Based Chemically Recyclable Polymers Enabled by Fused-Ring Monomers. Nat. Chem. 2021, 13, 743–750. 10.1038/s41557-021-00748-5. 34294914

[ref7] aLiu Y.; Jia Y.; Wu Q.; Moore J. S. Architecture-Controlled Ring-Opening Polymerization for Dynamic Covalent Poly(Disulfide)S. J. Am. Chem. Soc. 2019, 141 (43), 17075–17080. 10.1021/jacs.9b08957. 31603692

[ref8] aKultys A. Sulfur-Containing Polymers. In Encyclopedia of Polymer Science and Technology; John Wiley & Sons, Inc., 2002.

[ref9] Purohit V. B.; Pieta M.; Pietrasik J.; Plummer C. M. Recent advances in the ring-opening polymerization of sulfur-containing monomers. Polym. Chem. 2022, 13 (34), 4858–4878. 10.1039/D2PY00831A.

[ref10] Mavila S.; Worrell B. T.; Culver H. R.; Goldman T. M.; Wang C.; Lim C.-H.; Domaille D. W.; Pattanayak S.; McBride M. K.; Musgrave C. B.; Bowman C. N. Dynamic and Responsive DNA-like Polymers. J. Am. Chem. Soc. 2018, 140, 13594–13598. 10.1021/jacs.8b09105. 30351134

[ref11] López-Vidal E. M.; Gregory G. L.; Kociok-Köhn G.; Buchard A. Polymers from sugars and CS_2_: synthesis and ring-opening polymerisation of sulfur-containing monomers derived from 2-deoxy-d-ribose and d-xylose. Polym. Chem. 2018, 9, 1577–1582. 10.1039/C8PY00119G.

[ref12] aEvans R. A.; Rizzardo E. Free-Radical Ring-Opening Polymerization of Cyclic Allylic Sulfides. Macromolecules 1996, 29, 6983–6989. 10.1021/ma960573p.

[ref13] Su Y. L.; Yue L.; Tran H.; Xu M. Z.; Engler A.; Ramprasad R.; Qi H. J.; Gutekunst W. R. Chemically Recyclable Polymer System Based on Nucleophilic Aromatic Ring-Opening Polymerization. J. Am. Chem. Soc. 2023, 145, 13950–13956. 10.1021/jacs.3c03455. 37307298 PMC10311534

[ref14] aRowan S. J.; Cantrill S. J.; Cousins G. R. L.; Sanders J. K. M.; Stoddart J. F. Dynamic covalent chemistry. Angew. Chem., Int. Ed. 2002, 41, 898–952. 10.1002/1521-3773(20020315)41:6<898::AID-ANIE898>3.0.CO;2-E. 12491278

[ref15] aNair D. P.; Podgórski M.; Chatani S.; Gong T.; Xi W.; Fenoli C. R.; Bowman C. N. The Thiol-Michael Addition Click Reaction: A Powerful and Widely Used Tool in Materials Chemistry. Chem. Mater. 2014, 26 (1), 724–744. 10.1021/cm402180t.

[ref16] aJoshi G.; Anslyn E. V. Dynamic Thiol Exchange with β-Sulfido-α,β-Unsaturated Carbonyl Compounds and Dithianes. Org. Lett. 2012, 14, 4714–4717. 10.1021/ol301781u. 22934665 PMC3472802

[ref17] aIshibashi J. S. A.; Kalow J. A. Vitrimeric Silicone Elastomers Enabled by Dynamic Meldrum’s Acid-Derived Cross-Links. ACS Macro Lett. 2018, 7, 482–486. 10.1021/acsmacrolett.8b00166. 35619346

[ref18] Lu B.; Li Y.; Wang Y.; Aue D. H.; Luo Y.; Zhang L. [3,3]- Sigmatropic Rearrangement versus Carbene Formation in Gold-Catalyzed Transformations of Alkynyl Aryl Sulfoxides: Mechanistic Studies and Expanded Reaction Scope. J. Am. Chem. Soc. 2013, 135, 8512–8524. 10.1021/ja401343p. 23731178 PMC3704346

[ref19] aHendrickson J. B. Molecular Geometry. V. Evaluation of Functions and Conformations of Medium Rings. J. Am. Chem. Soc. 1967, 89, 7036–7043. 10.1021/ja01002a036.

