# Supplementary material for: Michael Addition–Elimination Ring-Opening Polymerization
Source: J Am Chem Soc. 2024 Jun 21;146(26):18074–82. doi: 10.1021/jacs.4c05054 (PMC11228986; doi:10.1021/jacs.4c05054)
Supplement: Supplementary file 1 — ja4c05054_si_001.pdf [file ja4c05054_si_001.pdf]

Supporting Information

Michael Addition-Elimination Ring-Opening Polymerization

Yong-Liang Su[1], Wei Xiong[1], Liang Yue[2], Mckinley K. Paul[1], Kaitlyn S. Otte[1], John Bacsa[3], H. Jerry Qi[2] and Will R. Gutekunst[1]*

[1]*School of Chemistry and Biochemistry, Georgia Institute of Technology, 901 Atlantic Drive NW, Atlanta, Georgia 30332, United States.*

[2]*School of Mechanical Engineering, Georgia Institute of Technology, Atlanta, Georgia 30332, United States*

[3]*Department of Chemistry, Emory University, Atlanta, Georgia 30322, United States*

* E-mail: *willgute@gatech.edu*

Table of Contents

1. General information S2
2. Synthesis of monomers S3
3. Optimization of the polymerization conditions S6
4. General polymerization procedure and characterization of polymers S8
5. **TP-C=O** ring opening reaction S12
6. Characterizing polymer chain-ends using MALDI-TOF S13
7. TGA and DSC studies of the **PTCs** S13
8. Mechanical properties of the **PTC-OAc** S14
9. Chemical recycling of **PTC-C=O** and **PTC-C=C** S14
10. The stability of **PTC-C=O** in the presence of MeOH and pyrrolidine S16
11. Polymerization thermodynamic studies of **TC-C=O** S16
12. X-ray crystal structure of the monomers **TC-C=O** and **TC-OAc** S18
13. Analysis of conformational and steric effects S21
14. Computational details S21
15. NMR spectrum of products S47
16. References S61

1. General information

General methods. All reactions were carried out under a nitrogen atmosphere with dry solvents using anhydrous conditions unless otherwise stated. Dry, degassed *N*,*N*-dimethylformamide (DMF), acetonitrile (CH_3CN), and tetrahydrofuran (THF) were obtained from a JC Meyer solvent purification system. Dimethyl sulfoxide (DMSO), dimethylacetamide (DMA), *N*-methyl-2-pyrrolidone (NMP), *N*,*N'*-dimethylpropyleneurea (DMPU), nitrobenzene ($PhNO_2$), chloroform ($CHCl_3$) and 1,2-dichloroethane (DCE) were purchased from commercial sources and further dried with activated 4Å molecular sieves and degassed. Unless otherwise stated, all other reagents were purchased at the highest commercial quality and used without further purification. Yields refer to chromatographically and spectroscopically (^{1}H-NMR) homogeneous materials, unless otherwise stated. Reactions were monitored by thin layer chromatography (TLC) carried out on 0.25 mm E. Merck silica gel plates (60F-254) using UV light as the visualizing agent and basic aqueous potassium permanganate ($KMnO_4$), and heat as developing agents. E. Merck silica gel (60, particle size 0.043–0.063 mm) was used for flash column chromatography. NMR spectra were recorded on Bruker Avance 400, 500 or 700 MHz instruments and calibrated using residual undeuterated solvent as an internal reference ($CHCl_3$ @ 7.26 ppm ^{1}H NMR, 77.16 ppm ^{13}C NMR). The following abbreviations (or combinations thereof) were used to explain the multiplicities: s = singlet, d = doublet, t = triplet, q = quartet, m = multiplet, br = broad, comp = composite of magnetically non-equivalent protons. Mass spectra (MS) were recorded on LC/MS (Agilent Technologies 1260 Infinity II/6120 Quadrupole) or a time-of-flight matrix assisted laser desorption/ionization (MALDI-TOF) using a *trans*-2-[3-(4-*tert*-butylphenyl)-2-methyl-2-propenylidene]malononitrile (DCTB) matrix. Polymer samples were analyzed using a Tosoh EcoSEC HLC 8320GPC system with TSKgel SuperHZ-L columns eluting $CHCl_3$ containing 0.25% NEt_3 at a flow rate of 0.45 mL/min. All number-average molecular weights and dispersities were calculated from refractive index chromatograms using PStQuick Mp-M polystyrene standards. Thermogravimetric analyses (TGA) were performed under nitrogen atmosphere on a Pyris 1 TGA (PerkinElmer) at a heating rate of 10 °C/min. Differential scanning calorimetry (DSC) analyses were measured on a DSC 3+ STARe system (Mettler Toledo). The reported data were obtained from the third heating cycle at a heating rate of 10 °C/min. Melting points were measured on a MEL-TEMP II Laboratory Devices (uncorrected).

Note 1: Due to the potential toxicity and health hazards, it is essential to take precautions when working with mercuric triflate. Always work in a well-ventilated fume hood to avoid inhalation of fumes. Wear appropriate personal protective equipment, including gloves, safety goggles, and lab coats. Handle the chemical with care to avoid skin contact, ingestion, or inhalation. Additionally, generate a separate waste stream for mercuric triflate disposal to ensure it is handled and processed safely.

2. Synthesis of monomers

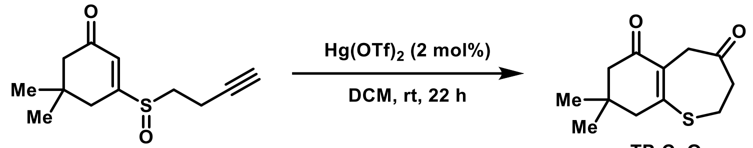

TP-C=O

The monomer **TP-C=O** was prepared according to the reported literature.[1] To an oven-dried flask was added sulfoxide (1.0 g, 4.5 mmol), $Hg(OTf)_2$ (44.9 mg, 2 mol %). and dry DCM (90 mL). The mixture was stirred for 24 h at the room temperature. The reaction mixture was concentrated under vacuum. The residue was purified by column chromatography on silica gel (eluent: hexanes/ethyl acetate = 2:1) to give product **TP-C=O** (pale yellow liquid, 73% yield, 0.73 g).

Note 1: Due to the potential toxicity and health hazards, it is essential to take precautions when working with mercuric triflate. Always work in a well-ventilated fume hood to avoid inhalation of fumes. Wear appropriate personal protective equipment, including gloves, safety goggles, and lab coats. Handle the chemical with care to avoid skin contact, ingestion, or inhalation. Additionally, generate a separate waste stream for mercuric triflate disposal to ensure it is handled and processed safely.

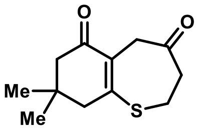

TP-C=O

8,8-Dimethyl-2,3,5,7,8,9-hexahydrobenzo[*b*]thiepine-4,6-dione. **^{1}H NMR** (700 MHz, chloroform-*d*) δ 3.80 (s, 2H), 3.26 – 3.20 (m, 2H), 3.02 – 2.94 (m, 2H), 2.33 (s, 2H), 2.29 (s, 2H), 1.01 (s, 6H). **^{13}C NMR** (176 MHz, chloroform-*d*) δ 205.1, 193.6, 156.6, 124.7, 50.7, 46.5, 46.3, 37.7, 33.3, 27.8, 27.4. Peak overlapping was observed. **MS (m/z)**: calcd for $C_{12}H_{17}O_2S$, $[M+H]^+$: 225.09; found, 225.1.

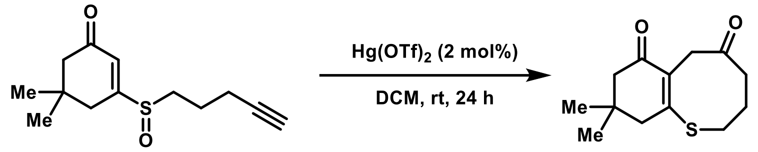

TC-C=O

The monomer **TC-C=O** was prepared according to the reported literature.[1] To an oven-dried flask was added sulfoxide (5.96 g, 25.0 mmol), $Hg(OTf)_2$ (249.4 mg, 2 mol %). and dry DCM (500 mL). The mixture was stirred for 24 h at the room temperature. The reaction mixture was concentrated under vacuum. The residue was purified by column chromatography on silica gel (eluent: hexanes/diethyl ether = 1:1.5) to give product **TC-C=O** (white solid, 92% yield, 5.48 g). The spectral data were in accordance with those reported in the literature.[1]

Note 1: Due to the potential toxicity and health hazards, it is essential to take precautions when working with mercuric triflate. Always work in a well-ventilated fume hood to avoid inhalation of fumes. Wear appropriate personal protective equipment, including gloves, safety goggles, and lab coats. Handle the chemical with care to avoid skin contact, ingestion, or inhalation. Additionally, generate a separate waste stream for mercuric triflate disposal to ensure it is handled and processed safely.

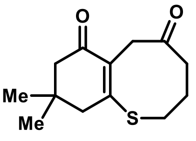

TC-C=O

9,9-Dimethyl-3,4,6,8,9,10-hexahydro-2*H*-benzo[*b*]thiocine-5,7-dione.
^{1}H NMR (500 MHz, chloroform-*d*) δ 3.62 (t, *J* = 1.7 Hz, 2H), 2.96 – 2.86 (m, 2H), 2.58 (t, *J* = 1.6 Hz, 2H), 2.44 – 2.39 (m, 2H), 2.35 (s, 2H), 2.25 – 2.18 (m, 2H), 1.08 (s, 6H). **^{13}C NMR** (176 MHz, chloroform-*d*) δ 210.6, 196.2, 154.5, 137.2, 50.6, 48.6, 42.7, 39.2, 33.7, 33.3, 30.1, 28.1. Peak overlapping was observed. **MS (m/z)**: calcd for $C_{13}H_{19}O_2S$, $[M+H]^+$: 239.11; found, 239.2. The spectral data were in accordance with those reported in the literature.[1]

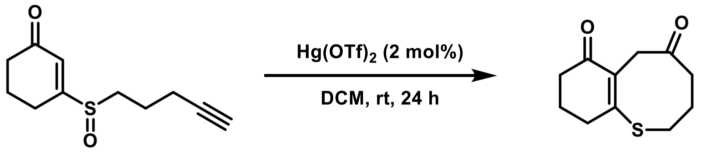

TC-CHD

The monomer **TC-CHD** was prepared according to the reported literature.[1] To an oven-dried flask was added sulfoxide (0.34 g, 1.6 mmol), $Hg(OTf)_2$ (249.4 mg, 2 mol %). and dry DCM (30 mL). The mixture was stirred for 24 h at the room temperature. The reaction mixture was concentrated under vacuum. The residue was purified by column chromatography on silica gel (eluent: DCM/diethyl ether = 10:1) to give product **TC-CHD** (colorless oil, 36% yield, 0.12 g), and 0.16 g of the starting material was recovered.

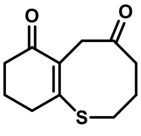

TC-CHD

3,4,6,8,9,10-Hexahydro-2*H*-benzo[*b*]thiocine-5,7-dione.
^{1}H NMR (700 MHz, chloroform-*d*) δ 3.61 (t, *J* = 1.6 Hz, 2H), 2.97 – 2.94 (m, 2H), 2.69 (ddd, *J* = 6.0, 4.3, 1.6 Hz, 2H), 2.49 (dd, *J* = 7.4, 6.0 Hz, 2H), 2.46 – 2.41 (m, 2H), 2.20 (tt, *J* = 8.1, 5.3 Hz, 2H), 2.07 – 2.01 (m, 2H). **^{13}C NMR** (176 MHz, chloroform-*d*) δ 210.6, 195.9, 157.3, 137.6, 42.9, 39.1, 37.2, 34.6, 34.1, 29.8, 22.7. **MS (m/z)**: calcd for $C_{11}H_{15}O_2S$, $[M+H]^+$: 211.08; found, 211.1.

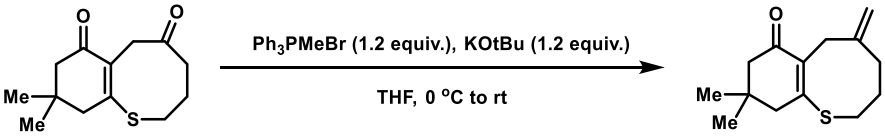

TC-C=C

To a flask were added PPh_3MeBr (0.64 g, 1.8 mmol, 1.2 equiv.), anhydrous THF (20 mL) and a stirring bar, and the system was cooled to 0 °C. Then ^{t}BuOK (0.20 g, 1.8 mmol, 1.2 equiv.) was added in portions and the mixture was stirred for 30 min. The solution of **TC-C=O** (0.36 g, 1.5 mmol, 1.0 equiv.) in anhydrous THF (10 mL) was added into the reaction mixture dropwise. After stirring at rt for 6 h, the reaction was quenched with H_2O (30 mL) and the mixture was extracted with DCM (3×30 mL). The combined organic layers were washed with brine, dried over anhydrous Na_2SO_4 and concentrated *in vacuo*. The crude mixture was purified by column chromatography on silica gel (eluent: hexanes/ethyl acetate = 10:1) to give product **TC-C=C** (white solid, 71% yield, 0.25 g).

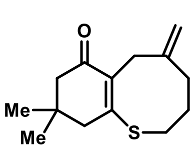

TC-C=C

9,9-Dimethyl-5-methylene-2,3,4,5,6,8,9,10-octahydro-7*H*-benzo[*b*]thiocin-7-one.
^{1}H NMR (400 MHz, chloroform-*d*) δ 4.93 – 4.92 (m, 1H), 4.82 – 4.80 (m, 1H), 3.54 (s, 2H), 3.33 – 3.22 (m, 2H), 2.37 (s, 2H), 2.33 – 2.30 (m, 2H), 2.25 (s, 2H), 2.05 – 1.99 (m, 2H), 1.01 (s, 6H). **^{13}C NMR** (176 MHz, chloroform-*d*) δ 195.3,

157.4, 147.2, 130.3, 113.5, 51.0, 47.0, 34.3, 33.3, 32.5, 32.3, 32.0, 27.9. Peak overlapping was observed. **MS (m/z)**: calcd for $C_{14}H_{21}OS$, $[M+H]^+$: 237.13; found, 237.2.

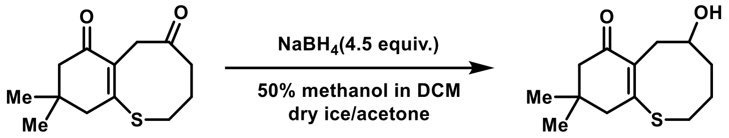

To a flask were added **TC-C=O** (3.34 g, 14 mmol), DCM (100 mL), MeOH (100 mL) and a stirring bar, and the system was cooled in a dry ice-acetone bath. Then $NaBH_4$ (2.38 g, 63.0 mmol, 4.5 equiv.) was added in portions and the mixture was stirred for 2 h. The reaction was quenched with acetone 10 mL) and the mixture was extracted with DCM (3×100 mL). The combined organic layers were washed with brine, dried over anhydrous Na_2SO_4 and concentrated *in vacuo*. The crude mixture was purified by column chromatography on silica gel (eluent: hexanes/ethyl acetate = 2:1) to give product **TC-OH** (white solid, 96% yield, 3.23 g).

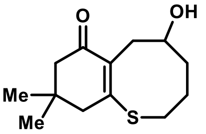

TC-OH

5-Hydroxy-9,9-dimethyl-2,3,4,5,6,8,9,10-octahydro-7*H*-benzo[*b*]thiocin-7-one.

^{1}H NMR (700 MHz, chloroform-*d*) δ 4.10 – 4.02 (m, 1H), 3.56 – 3.47 (m, 1H), 3.03 – 2.88 (m, 3H), 2.42 (d, *J* = 17.0 Hz, 1H), 2.36 (d, *J* = 17.0 Hz, 1H), 2.30 (s, 2H), 2.13 – 2.04 (m, 1H), 1.96 – 1.88 (m, 1H), 1.87 – 1.83 (m, 1H), 1.74 – 1.66 (m, 1H), 1.05 (s, 3H), 1.03 (s, 3H). **^{13}C NMR** (176 MHz, chloroform-*d*) δ 198.7, 160.1, 128.6, 72.0, 50.7, 46.9, 34.0, 33.6, 31.4, 31.0, 28.1, 27.8, 26.1. **MS (m/z)**: calcd for $C_{13}H_{21}O_2S$, $[M+H]^+$: 241.13; found, 241.1.

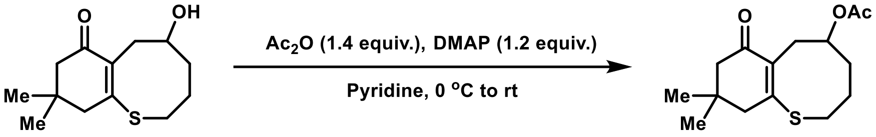

To a solution of the alcohol substrate **TC-OH** (1.92 g, 8 mmol) in pyridine (50 mL) were added Ac_2O (1.14 g, 11.2 mmol, 1.4 equiv.) and DMAP (1.17 g, 9.6 mmol, 1.2 equiv.) under 0 °C. After stirring at rt for 1.5 h. The volatile components were evacuated under reduced pressure. Subsequently, 50 mL H_2O was added, followed by extraction with EtOAc (3×60 mL). The combined organic layers were washed with brine, dried over anhydrous Na_2SO_4 and concentrated *in vacuo*. The crude mixture was purified by column chromatography on silica gel (eluent: hexanes/ethyl acetate = 10:1) to give product **TC-OAc** (white solid, 80% yield, 1.81 g).

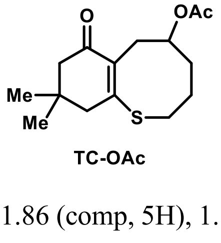

TC-OAc

9,9-Dimethyl-7-oxo-3,4,5,6,7,8,9,10-octahydro-2*H*-benzo[*b*]thiocin-5-yl acetate.

^{1}H NMR (500 MHz, chloroform-*d*) δ 5.04 (tdd, *J* = 7.8, 4.7, 2.9 Hz, 1H), 3.32 – 3.21 (m, 2H), 3.09 (dd, *J* = 14.5, 7.5 Hz, 1H), 2.96 (dd, *J* = 14.5, 4.7 Hz, 1H), 2.44 – 2.31 (m, 2H), 2.30 – 2.19 (m, 2H), 2.15 – 2.08 (m, 1H), 2.03 – 1.86 (comp, 5H), 1.82 – 1.74 (m, 1H), 1.04 (s, 3H), 1.02 (s, 3H). **^{13}C NMR** (176 MHz, chloroform-*d*) δ 195.3, 170.4, 158.9, 127.6, 73.9, 50.8, 46.9, 33.5, 33.3, 27.9, 27.7, 27.3, 26.7, 25.9, 21.5. **MS (m/z)**: calcd for $C_{15}H_{23}O_3S$, $[M+H]^+$: 283.14; found, 283.2.

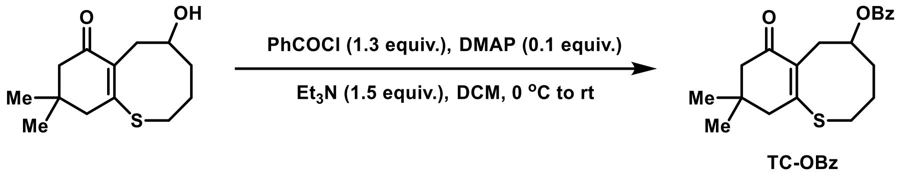

To a solution of the alcohol substrate **TC-OH** (0.36 g, 1.5 mmol) in anhydrous DCM (20 mL) were added DMAP (18.3 mg, 0.15 mmol, 0.1 equiv.) and Et_3N (0.23 g, 2.25 mmol, 1.5 equiv.) under 0 °C. Then, benzoyl chloride (0.27 g, 1.95 mmol, 1.3 equiv.) was added dropwise. After stirring at rt for 16 h, the reaction mixture was quenched by H_2O (30 mL), then extracted with EtOAc (3×40 mL). The combined organic layers were washed with brine, dried over anhydrous Na_2SO_4 and concentrated *in vacuo*. The crude mixture was purified by column chromatography on silica gel (eluent: hexanes/ethyl acetate = 5:1) to give product **TC-OBz** (white solid, 81% yield, 0.42 g).

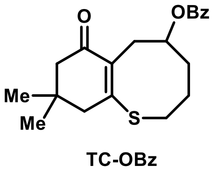

9,9-Dimethyl-7-oxo-3,4,5,6,7,8,9,10-octahydro-2*H*-benzo[*b*]thiocin-5-yl benzoate.
^{1}H NMR (700 MHz, chloroform-*d*) δ 8.07 – 7.92 (comp, 2H), 7.57 – 7.50 (m, 1H), 7.45 – 7.38 (comp, 2H), 5.35 – 5.26 (m, 1H), 3.45 – 3.35 (m, 1H), 3.30 – 3.20 (m, 2H), 3.15 (dd, J = 14.4, 5.0 Hz, 1H), 2.41 (s, 2H), 2.33 – 2.24 (m, 2H), 2.23 – 2.16 (m, 1H), 2.15 – 2.09 (m, 1H), 2.02 – 1.92 (m, 2H), 1.07 (s, 3H), 1.05 (s, 3H). **^{13}C NMR** (176 MHz, chloroform-*d*) δ 195.2, 165.8, 159.0, 132.8, 130.9, 129.7, 128.4, 127.7, 74.6, 50.9, 47.0, 33.7, 33.3, 28.1, 27.6, 27.4, 27.0, 26.1. **MS (m/z)**: calcd for $C_{20}H_{25}O_3S$, $[M+H]^+$: 345.15; found, 345.2.

3. Optimization of the polymerization conditions

Table S1. Investigation of solvents for the polymerization[a]

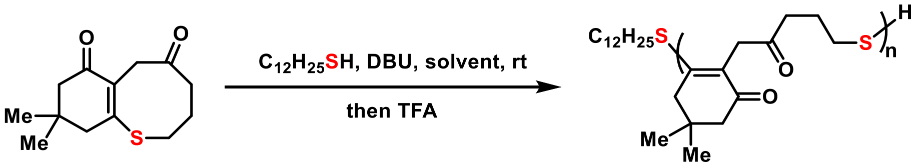

entry	solvent	concentration (M)	time (min)	conversion (%)[b]	$M_{n,SEC}$ (kDa)[c]	$Đ^c$
1	DMF	1.5	15	95	26.0	1.49
2	DMSO	1.5	5	>99	32.3	1.69
3	CH_3CN	1.5	15	99	31.0	1.62
4	$PhCF_3$	1.0	180	65	12.1	1.21
5	THF	1.5	180	84	17.6	1.29
6	1,4-Dioxane	1.5	1380	95	21.5	1.41
7	DCM	1.5	1320	0	-	-
8	THF/1,4-Dioxane (1/1)	1.5	420	92	18.8	1.20

9[d]	THF/1,4-Dioxane (1/1)	1.5	90	91	11.4	1.22

[a][M]$_0$/[I]$_0$/[base]$_0$ = 50/1/1. [b]Conversions were determined by ^{1}H NMR spectroscopic analysis of the reaction mixture. [c]Molecular weights ($M_{n,SEC}$)and dispersities ($Đ$)were determined by size-exclusion chromatography. [d][M]$_0$/[I]$_0$/[base]$_0$ = 25/1/1, under air conditions, a shoulder peak was observed in the GPC trace, which may be due to disulfide formation.

Table S2. Investigation of temperature and concentration for the polymerization

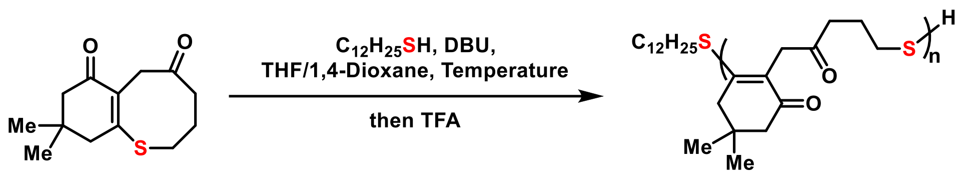

entry	temperature (°C)	concentration (M)	time (min)	conversion (%)[c]	$M_{n,SEC}$ (kDa)[d]	$Đ$[d]
1[a]	rt	1.0	1380	90	17.2	1.21
2[a]	rt	1.0	2520	98	20.2	1.32
3[a]	35	2.0	120	94	17.9	1.25
4[b]	rt	1.5	1440	87	34.4	1.43
5[b]	35	1.0	2730	94	35.3	1.51
6[b]	35	1.5	1320	83	31.5	1.47
7[b]	35	2	360	87	31.2	1.44

[a][M]$_0$/[I]$_0$/[base]$_0$ = 50/1/1. [b][M]$_0$/[I]$_0$/[base]$_0$ = 100/1/1. [c]Conversions were determined by ^{1}H NMR spectroscopic analysis of the reaction mixture. [d]Molecular weights ($M_{n,SEC}$)and dispersities ($Đ$)were determined by size-exclusion chromatography.

Table S3. Investigation of initiators for the polymerization[a]

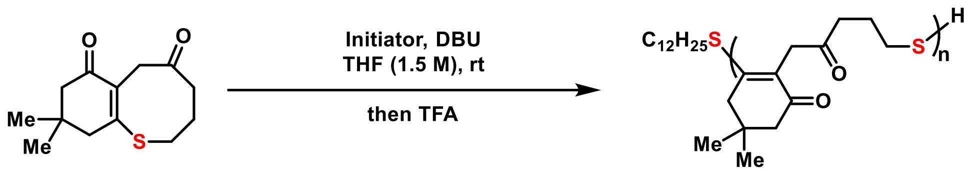

entry	Initiator	pK_a^{DMSO}	time (min)	conversion (%)[b]	$M_{n,SEC}$ (kDa)[c]	$Đ$[c]
1	BnSH	15.4	1290	>99	24.9	1.68
2	PhSH	10.3	1440	>99	27.9	1.72
3	without RSH	-	1320	0	-	-
4[d]	BnOH	-	1080	<5	-	-
5[e]	BnOH	-	2700	5	7.9	1.38
6[d]	pyrrolidine	44	1080	<5	-	-
7[e]	pyrrolidine	44	2700	15	9.4	2.32

[a][M]$_0$/[I]$_0$/[base]$_0$ = 50/1/1. [b]Conversions were determined by ^{1}H NMR spectroscopic analysis of the reaction mixture. [c]Molecular weights ($M_{n,SEC}$)and dispersities ($Đ$)were determined by size-exclusion chromatography.

[d][M]$_0$/[I]$_0$/[base]$_0$ = 25/1/1, THF/1,4-Dioxane (1/1) was used as the solvent. [e][M]$_0$/[I]$_0$/[base]$_0$ = 25/1/1, DMF was used as the solvent.

Table S4. Investigation of bases for the polymerization[a]

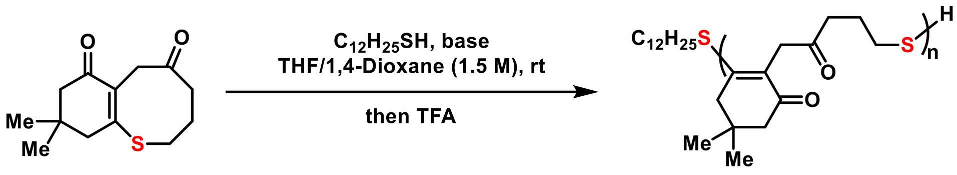

entry	base	$pK_a^{CH_3CN}$	time (min)	conversion (%)[b]	$M_{n,SEC}$ (kDa)[c]	$Đ$[c]
1	Et_3N	18.46	1320	0	-	-
2	DMAP	17.95	1200	0	-	-
3	TBD	26.03	30	>99	18.3	1.97

[a][M]$_0$/[I]$_0$/[base]$_0$ = 50/1/1. [b]Conversions were determined by ^{1}H NMR spectroscopic analysis of the reaction mixture. [c]Molecular weights ($M_{n,SEC}$)and dispersities ($Đ$)were determined by size-exclusion chromatography.

4. General polymerization procedure and characterization of polymers

Preparation of stock solution: The desired amounts of 1-dodencanethiol and DBU were added into an oven-dried 2 mL vial under N_2. Dry, degassed THF and 1,4-dioxane were then added to make a stock solution.

To an oven-dried microwave vial equipped with a magnetic stir bar was added the thiocane monomer **TC** (0.2 mmol). Following the evacuation and triple backfilling with N_2, the vial was charged with dry, degassed THF and 1,4-dioxane, along with the initiator stock solution. After stirring for the indicated time, the reaction was quenched by three drops of trifluoracetic acid. An aliquot of the reaction mixture was taken for ^{1}H NMR to determine the conversion of the monomer. Another aliquot of the reaction mixture was taken for SEC analysis. The resulting polymer was precipitated from cold MeOH. The purified polymer was then characterized using SEC, ^{1}H-NMR, ^{13}C-NMR, TGA and DSC.

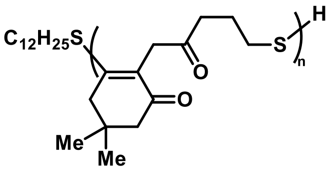

PTC-C=O

^{1}H NMR (700 MHz, chloroform-*d*) δ 3.53 (s, 2H), 2.85 (t, J = 7.6 Hz, 2H), 2.61 (t, J = 6.7 Hz, 2H), 2.54 (s, 2H), 2.28 (s, 2H), 1.86 (tt, J = 7.6, 6.7 Hz, 2H), 1.09 (s, 6H). **^{13}C NMR** (176 MHz, chloroform-*d*) δ 206.2, 194.6, 159.4, 128.0, 50.3, 42.8, 40.9, 40.6, 33.7, 30.4, 28.3, 24.3. Peak overlapping was observed.

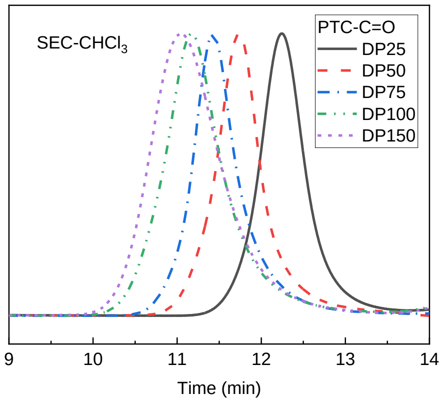

Figure S1. SEC trace for **PTC-C=O** targeting different DP.

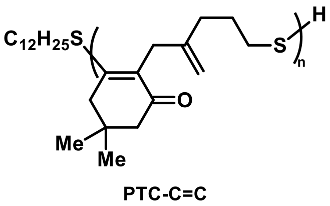

^{1}H NMR (700 MHz, chloroform-*d*) δ 4.71 (d, *J* = 1.3 Hz, 1H), 4.60 (d, *J* = 1.3 Hz, 2H), 3.14 (s, 2H), 2.86 (t, *J* = 7.5 Hz, 2H), 2.54 (s, 2H), 2.28 (s, 2H), 2.16 (t, *J* = 7.2 Hz, 2H), 1.83 (tt, *J* = 7.5, 7.2 Hz, 2H), 1.08 (s, 6H). **^{13}C NMR** (176 MHz, chloroform-*d*) δ 194.6, 158.6, 144.7, 131.1, 110.2, 50.7, 43.1, 35.7, 33.5, 32.5, 30.4, 28.5, 28.1. Peak overlapping was observed.

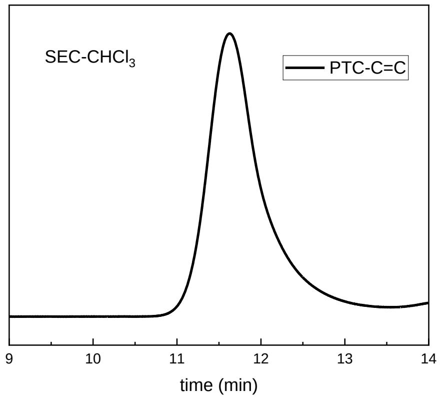

Figure S2. SEC trace of polymer **PTC-C=C**.

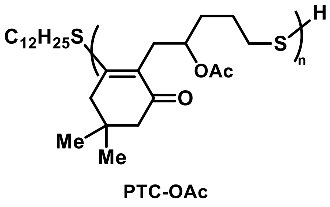

^{1}H NMR (700 MHz, chloroform-*d*) δ 5.06 – 5.02 (m, 1H), 2.89 – 2.77 (m, 2H), 2.73 – 2.66 (m, 2H), 2.50 – 2.38 (m, 2H), 2.27 – 2.20 (m, 2H), 1.97 (s, 3H), 1.72 – 1.58 (comp, 4H), 1.03 (s, 6H). **^{13}C NMR** (176 MHz, chloroform-*d*) δ 194.9, 170.7, 158.2, 130.1, 72.3, 50.6, 43.2, 34.0, 33.3, 31.9, 30.7, 28.3, 28.2, 26.3, 21.5.

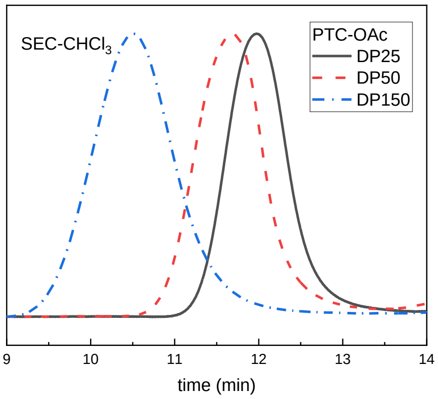

Figure S3. SEC trace of polymer **PTC-OAc** targeting different DP.

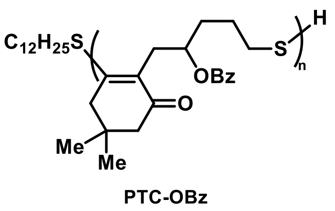

^{1}H NMR (700 MHz, chloroform-*d*) δ 8.01 – 7.94 (comp, 2H), 7.51 – 7.45 (m, 1H), 7.40 – 7.33 (comp, 2H), 5.32 – 5.26 (m, 1H), 2.88 – 2.65 (m, 4H), 2.37 – 2.23 (m, 2H), 2.21 – 2.09 (m, 2H), 1.81 – 1.70 (m, 2H), 1.68 – 1.57 (m, 2H), 0.90 – 0.87 (comp, 6H). **^{13}C NMR** (176 MHz, chloroform-*d*) δ 194.8, 166.2, 158.5, 133.0, 130.5, 129.9, 129.5, 128.4, 72.8, 50.6, 43.1, 34.1, 33.2, 31.7, 30.7, 28.2, 26.1.

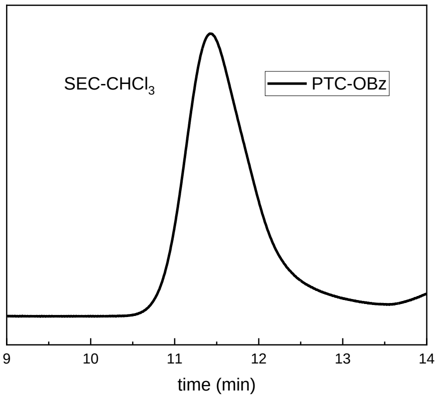

Figure S4. SEC trace of polymer **PTC-OBz**.

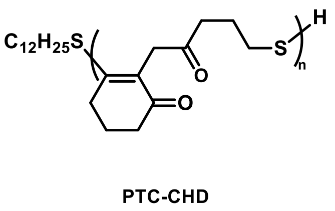

^{1}H NMR (700 MHz, chloroform-*d*) δ 3.52 (s, 2H), 2.86 (t, *J* = 6.2 Hz, 2H), 2.69 (t, *J* = 6.2 Hz, 2H), 2.61 (t, *J* = 6.6 Hz, 2H), 2.42 (t, *J* = 6.6 Hz, 2H), 2.06 (p, *J* = 6.2 Hz, 2H), 1.87 (p, *J* = 6.6 Hz, 2H). **^{13}C NMR** (176 MHz, chloroform-*d*) δ 206.3, 194.4, 162.0, 128.8, 41.1, 40.6, 36.7, 30.4, 29.2, 24.3, 22.6.

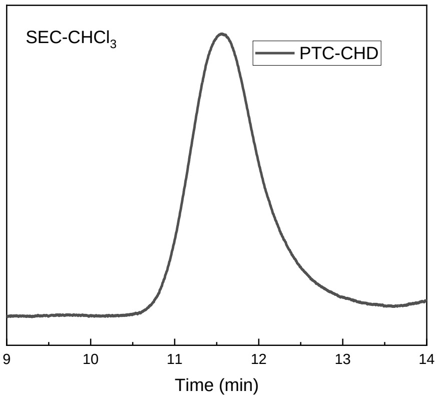

Figure S5. SEC trace of polymer **PTC-CHD**.

Copolymerization of monomers TC-C=O and TC-OAc

Following the general polymerization procedure described above, the polymerization reaction of **TC-C=O** targeting DP 25 was set up. After stirring at room temperature for 1.5 h, the co-solvents THF and 1,4-dioxane were removed through evacuation. A solution of **TC-OAc** in DMF was added to the reaction mixture. After stirring for an additional 16 h, the reaction was quenched by three drops of trifluoracetic acid. An aliquot of the reaction mixture was taken for ^{1}H-NMR to determine the conversion of the monomers. Another aliquot of the reaction mixture was taken for SEC analysis. The resulting polymer was precipitated from cold MeOH. The purified polymer was then characterized using SEC, ^{1}H-NMR, ^{13}C-NMR.

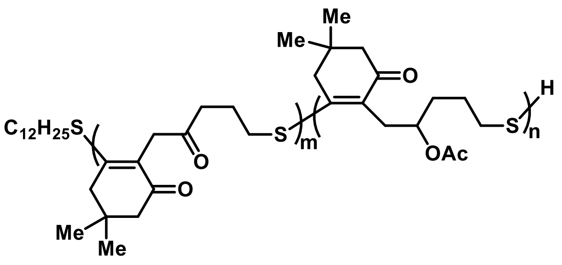

PTC-C=O-PTC-OAc copolymer

^{1}H NMR (700 MHz, chloroform-*d*) δ 5.08 – 4.98 (m, 1H), 3.53 (s, 3H), 2.90 – 2.75 (comp, 5H), 2.70 (t, *J* = 5.0 Hz, 2H), 2.61 (dt, *J* = 9.1, 6.6 Hz, 3H), 2.54 (s, 2H), 2.53 – 2.38 (comp, 3H), 2.28 (s, 3H), 2.28 – 2.19 (m, 2H), 1.97 (s, 3H), 1.86 (p, *J* = 6.8 Hz, 3H), 1.70 – 1.60 (comp, 4H), 1.09 (comp, 9H), 1.03 (s, 6H).

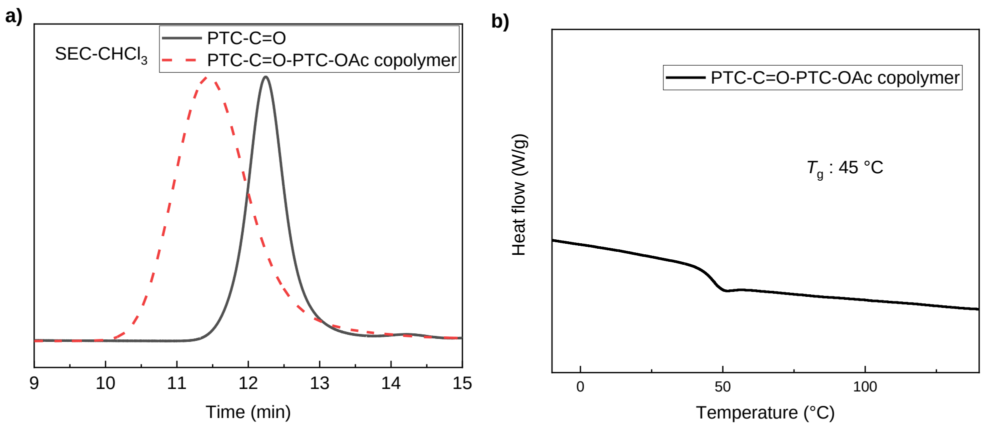

Figure S6. SEC trace and DSC curve of the **PTC-C=O-PTC-OAc** copolymer.

5. TP-C=O ring opening reaction

Under nitrogen atmosphere, to an oven-dried NMR tube were added **TP-C=O** (1.0 equiv.), benzyl mercaptan (1.0 equiv.), K_2CO_3 (1.5 equiv.) and dry d_6-DMSO. The reaction was monitored by ^{1}H NMR spectroscopy. After 18.5 h, the reaction was quenched with TFA. The crude mixture was purified by column chromatography on silica gel (eluent: hexanes/ethyl acetate = 5:1) to give product **TP-A**.

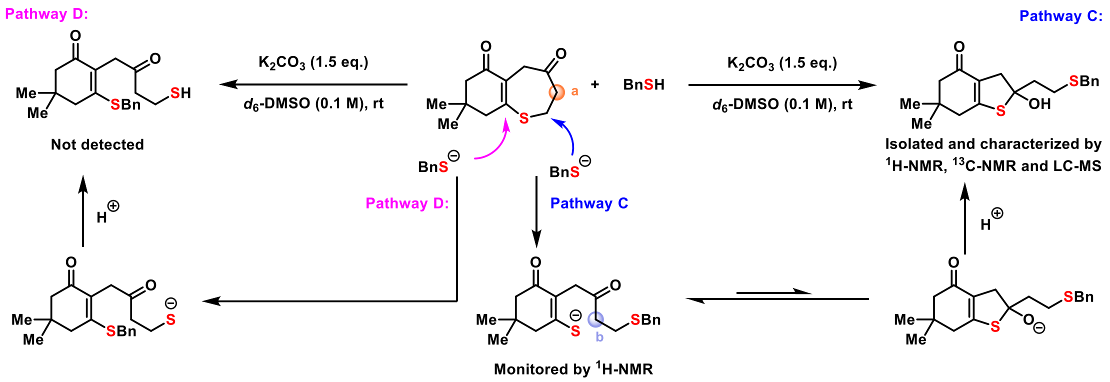

Figure S7. Ring-opening reaction of **TP-C=O**.

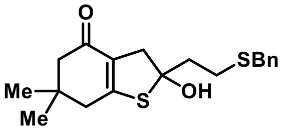

TP-A

^{1}H NMR (500 MHz, chloroform-*d*) δ 7.35 – 7.29 (comp, 4H), 7.27 – 7.23 (m, 1H), 3.76 (s, 2H), 3.70 – 3.46 (br, 1H), 3.20 – 3.12 (m, 1H), 3.06 – 2.99 (m, 1H), 2.71 – 2.58 (m, 2H), 2.51 – 2.41 (m, 1H), 2.38 – 2.32 (m, 1H), 2.30 – 2.20 (comp, 4H), 1.10 (s, 3H), 1.05 (s, 3H). **^{1}H NMR** (700 MHz, DMSO-d_6) δ 7.34 – 7.29 (comp, 4H), 7.25 – 7.21 (m, 1H), 6.89 (s, 1H), 3.74 (s, 2H), 2.95 – 2.83 (m, 2H), 2.57 – 2.52 (m, 1H), 2.47 – 2.42 (m, 1H), 2.40 – 2.33 (m, 2H), 2.20 – 2.07 (comp, 4H), 1.03 (s, 3H), 0.97 (s, 3H).**^{13}C NMR** (176 MHz, DMSO-d_6) δ 191.5, 161.2, 138.6, 128.9, 128.4, 127.8, 126.8, 98.1, 50.2, 45.6, 42.7, 35.1, 34.7, 28.1, 27.5, 26.4. Peak overlapping was observed. . **MS (m/z)**: calcd for $C_{19}H_{25}O_2S_2$, $[M+H]^+$: 349.13; found, 349.1.

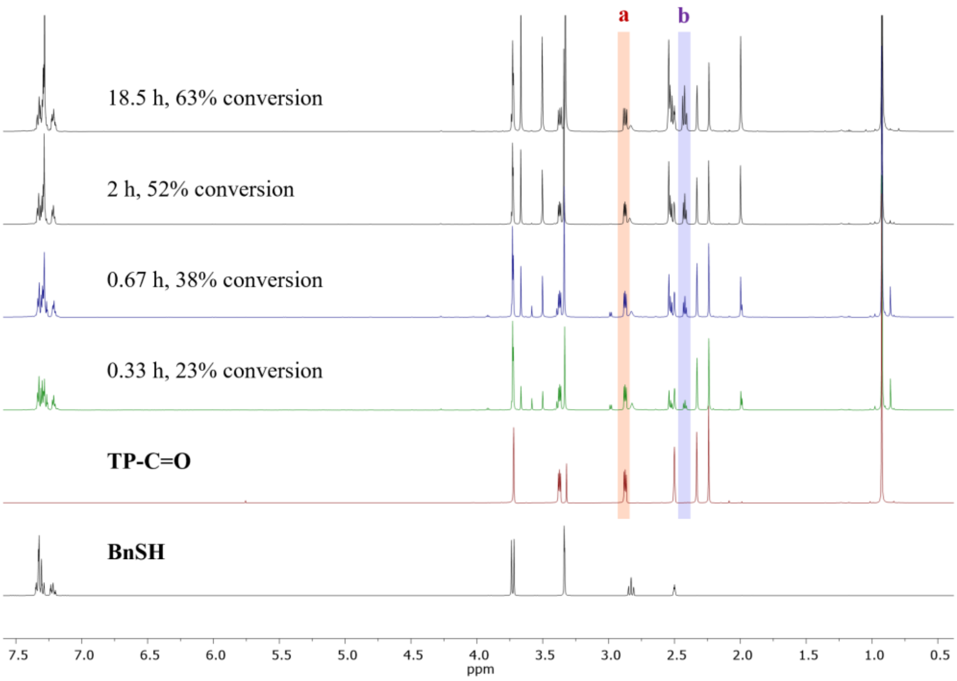

Figure S8. Overlay of ^{1}H-NMR spectra of ring-opening reaction between **TP-C=O** and benzyl mercaptan.

6. Characterizing polymer chain-ends using MALDI-TOF

Polymerization of TC-C=O targeting DP25:

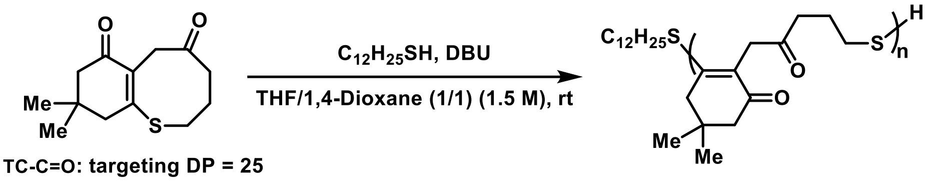

Preparation of stock solution: 1-dodencanethiol (20.24 mg, 24.0 μL, 0.1 mmol) and DBU (15.22 mg, 15.0 μL, 0.1 mmol) were added into an oven-dried 2 mL vial under N_2. Then dry THF/1,4-Dioxane (1/1, 211 μL) was added to make a stock solution.

To an oven-dried microwave vial equipped with a magnetic stir bar was added the **TC-C=O** monomer (0.2 mmol). After evacuation and backfilling with N_2 three times, dry THF/1,4-Dioxane (1/1, 63 μL) was added followed by the addition of initiator stock solution (20 μL). The total volume is around 133 μL and the initiating concentration of the monomer $[M]_0$ is around 1.5 M. After stirring for 1.5 h, the reaction was quenched with iodoacetamide (80 μL, 1 M). A sample of the crude material was taken for ^{1}H NMR to determine the conversion of the monomer. Another small amount of the crude material was taken for SEC analysis. The product was purified by precipitating from cold methanol (10 mL), then analyzed by matrix-assisted laser desorption/ionization-time-of-flight (MALDI-TOF) mass spectrometry.

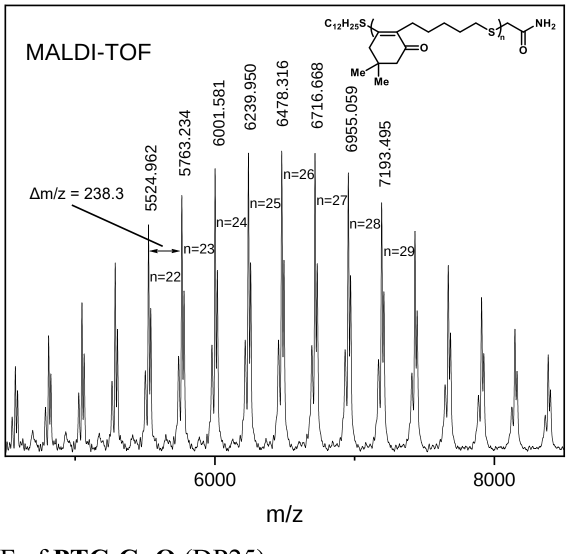

Figure S9. MALDI-TOF of **PTC-C=O** (DP25).

7. TGA and DSC studies of the PTCs

TGA of purified **PTCs** were obtained in a nitrogen atmosphere at a heating rate of 10 °C/min. The $T_{d,5\%}$ (temperature causing a 5% weight loss) of each **PTC** was listed in Figure S9a.

DSC analysis of purified **PTCs** were performed (-20 °C to 250 °C, heating rate: 10 °C/min, cooling

rate: 10 °C/min). The glass transition temperature (T_g) of each **PTC** was listed in Figure S9b.

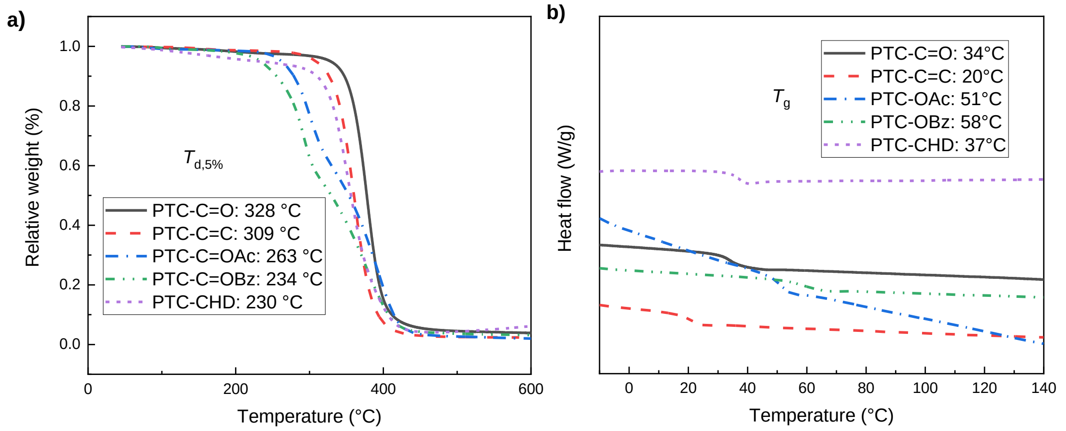

Figure S10. Thermal properties of **PTCs** a, TGA curves. b, DSC curves.

8. Mechanical properties of the PTC-OAc

PTC-OAc (M_n: 92.8 kDa, and $Đ$: 1.50) was prepared according to the general polymerization procedure. The polymer was compressed into 0.5mm films at 120 ℃ between two teflon sheets. Then, the sample was cut into strips (5mm x 30mm) for testing. Their thermomechanical properties were measured on a dynamic mechanical analysis tester (Q800, TA Instruments, New Castle, DE) with a frequency of 1 Hz in a tension mode with a temperature ramp 10 °C/min. The uniaxial tension tests were performed with a universal test machine (Insight 10, MTS Systems Corp., Eden Prairie, MN, USA) with a cross-head speed of 5 mm/min.

9. Chemical recycling of PTC-C=O and PTC-C=C

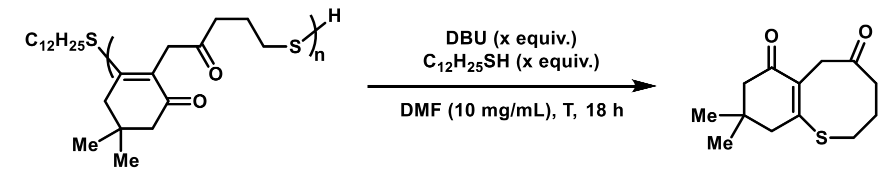

PTC-C=O (DP50, M_n 18.8 kDa, $Đ$ 1.20)

To a solution of **PTC-C=O** (DP50, 3.7 mg, 10^{-4} mmol, 1.0 equiv.; repeat unit: 1.54 × 10^{-2} mmol) in DMF was added the stock solution of DBU and $C_{12}H_{25}SH$ in DMF. After stirring for 18 h under the specified temperature, the reaction was quenched by TFA. The solvent was removed by evaporation and the residue was analyzed by ^{1}H NMR spectroscopy and SEC. The crude mixture from the conditions of 0.65 equiv. DBU and $C_{12}H_{25}SH$ under 150 °C was purified by column chromatography on silica gel (eluent: hexanes/ethyl acetate = 10:1) to give product **TC-C=O** (56% yield, 2.1 mg).

Table S5. Investigation of the reaction temperature and the amount of DBU and $C_{12}H_{25}SH$ for the depolymerization[a]

entry	temperature (℃)	DBU	$C_{12}H_{25}SH$	conversion (%)[b]
1	60	0.19 equiv.	0.19 equiv.	16%

2	90	0.19 equiv.	0.19 equiv.	30%
3	120	0.19 equiv.	0.19 equiv.	43%
4	150	0.13 equiv.	0.13 equiv.	52%
5	150	0.33 equiv.	0.33 equiv.	53%
6	150	0.65 equiv.	0.65 equiv.	57% (56%)[c]
7	150	0.98 equiv.	0.98 equiv.	47%
8	150	0.65 equiv.	0.65 equiv.[d]	54%
9	150	0.65 equiv.	0 equiv.	66% (63%)[c]

[a]The amount of DBU and $C_{12}H_{25}SH$ was relative to the repeat unit. [b]The conversion was determined by ^{1}H NMR spectroscopic analysis of the reaction mixture. [c]Isolated yield of recovered monomer in parentheses. [d]Pyrrolidine was used instead of $C_{12}H_{25}SH$.

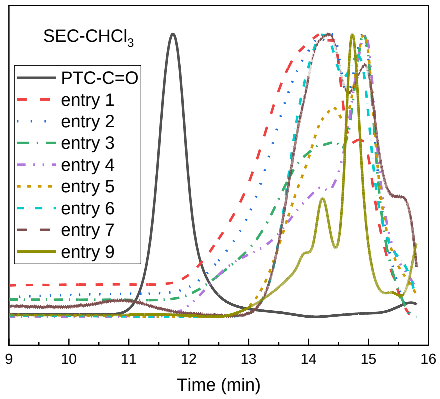

Figure S11. SEC curves for depolymerization of **PTC-C=O** under the conditions in Table S5.

The ESI analysis of the depolymerization reaction mixture (Table S5, entry 9) identified cyclic oligomers ranging from n = 2 to n = 5 (Figure S12).

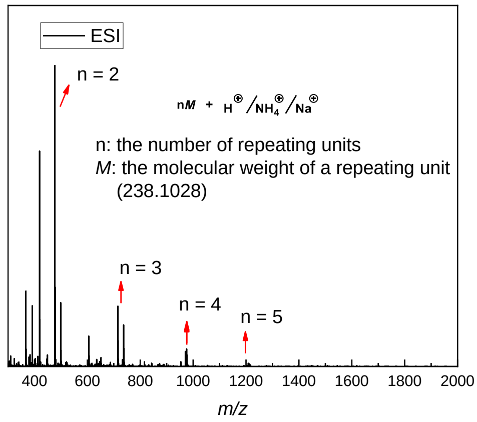

Figure S12. ESI analysis of the depolymerization of **PTC-C=O** under the conditions specified in entry 8 of Table S5.

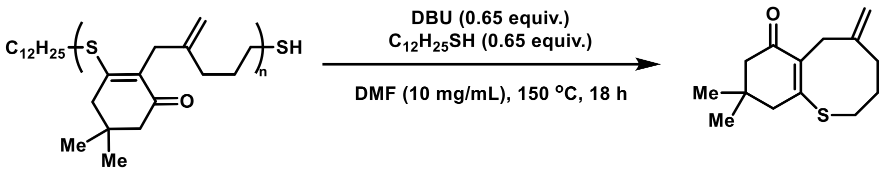

To a solution of **PTC-C=C** (DP50, 3.48 mg, 10^{-4} mmol, 1.0 equiv.; repeat unit: 1.45 × 10^{-2} mmol) in DMF was added the stock solution of DBU and $C_{12}H_{25}SH$ in DMF. After stirring for 18 h under 150 °C, the reaction was quenched by TFA. The solvent was removed by evaporation and the residue was analyzed by ^{1}H NMR spectroscopy and SEC. The crude mixture was purified by column chromatography on silica gel (eluent: hexanes/ethyl acetate = 10:1) to give product **TC-C=C** (42% yield, 1.48 mg).

10. The stability of PTC-C=O in the presence of MeOH and pyrrolidine

Table S6. Investigation of the stability of **PTC-C=O**

entry	solvent	nucleophile	time (h)
1[a]	MeOH	-	18
2	DCM	Pyrrolidine (10 equiv. relative to repeat unit)	18

[a]The polymer was suspended in MeOH but did not dissolve.

To a solution of **PTC-C=O** (DP50, 3.7 mg) in the specified solvent (10 mg/mL), the nucleophile was added. After stirring for 18 h at room temperature, the reaction was quenched by TFA. The solvent was then removed by evaporation and the residue was analyzed by ^{1}H NMR spectroscopy and SEC.

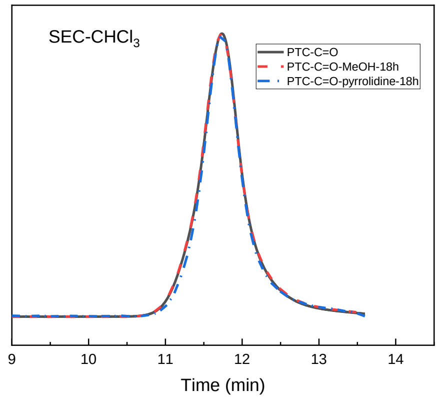

Figure S13. SEC curves for the original **PTC-C=O** and after treatment with MeOH and pyrrolidine**.**

11. Polymerization thermodynamic studies of TC-C=O

A representative procedure for the polymerization thermodynamic studies is shown as follows.

Preparation of stock solution: 1-dodencanethiol (20.24 mg, 24.0 µL, 0.1 mmol) and DBU (15.22 mg, 15.0 µL, 0.1 mmol) were added into an oven-dried 2 mL vial under N_2. Then dry DMF (211 µL) was added to make a stock solution.

To four oven-dried microwave vials equipped with magnetic stir bars were added the **TC-C**=O monomer (0.2 mmol in each vial). After evacuation and backfilling with N_2 three times, 940 µL

DMF was added into each vial. The vials were then placed into a preheated oil bath at different temperatures, including 80 °C, 100 °C, 120 °C and 140 °C. The initiator stock solution (20 µL) was added to the monomer solution. According to the kinetic studies, all polymerizations at 80 °C reached the equilibrium by 4 h, polymerizations at higher temperatures should require shorter time. After stirring for 4 h, the reaction was quenched by three drops of trifluoracetic acid and cooled to room temperature. The monomer concentration at the equilibrium was determined by ^{1}H NMR.

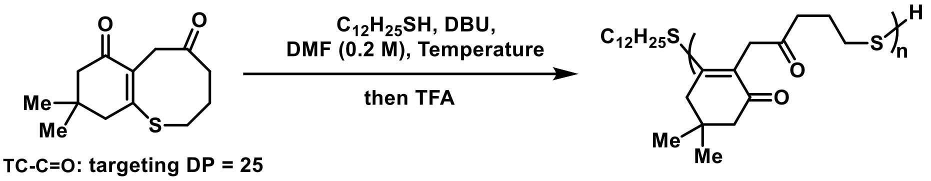

entry	temp. (°C)	temp. (K)	1/T (K^{-1})	conversion (%)	$[M]_0$	$[M]_e$	In $[M]_e$
1	80 °C	353	0.002833	87.8%	0.20 M	0.0244 M	-3.7132
2	100 °C	373	0.002681	81.3%	0.20 M	0.0374 M	-3.2861
3	120 °C	393	0.002545	73%	0.20 M	0.0540M	-2.9188
4	140 °C	413	0.002421	63.8%	0.20 M	0.0724 M	-2.6255

The thermodynamic parameters can be extracted by the linear fitting of the plot of $\ln[M]_e$ against *1/T* according to the following equation:

$$In[M]_e = \frac{\Delta H_p^0}{RT} - \frac{\Delta S_p^0}{R}$$

Here $[M]_e$ is the monomer concentration at thermodynamic equilibrium in mol L^{-1}, T is the reaction temperature in K^{-1}, ΔH_p^0 is the standard enthalpy change of polymerization in kJ mol^{-1}, ΔS_p^0 is the standard entropy change of polymerization in J mol^{-1} K^{-1}, and R is the gas constant (8.314 J mol^{-1} K^{-1}).

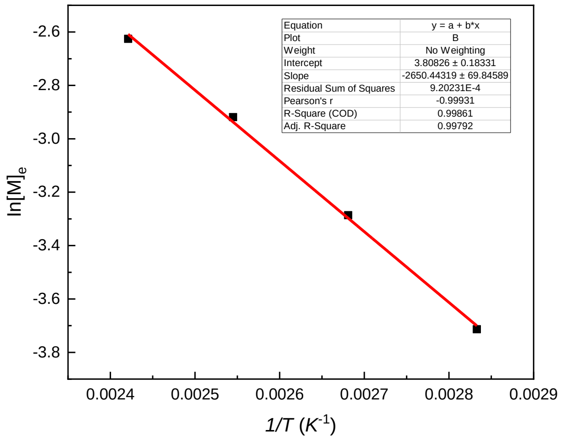

Figure S14. The van ‘t Hoff plot of **TC-C=O**.

$$\Delta H_p^0 = -2650 \times 8.314 \text{ J mol}^{-1} = -22.03 \text{ kJ mol}^{-1}$$

$$\Delta S_p^0 = -3.81 \times 8.314 \text{ J mol}^{-1} \text{ K}^{-1} = -31.68 \text{ J mol}^{-1} \text{ K}^{-1}$$

$$T_c = -22030 \text{ kJ mol}^{-1} \div (-31.68 \text{ J mol}^{-1} \text{ K}^{-1}) = 695.4 \text{ K}$$

12. X-ray crystal structure of the monomers TC-C=O and TC-OAc

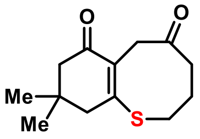

TC-C=O

≡

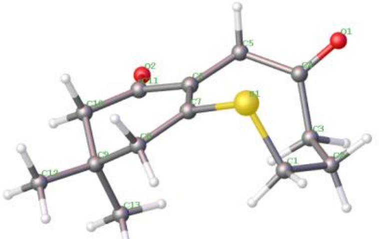

X-Ray of TC-C=O
CCDC: 2314461

Experimental. Single crystals of $C_{13}H_{18}O_2S$ **TC-C=O** were prepared by slow evaporation of hexane/ethyl acetate solution. Single colorless block-shaped crystals of **TC-C=O** were chosen from the sample. A suitable crystal with dimensions 0.42 × 0.29 × 0.22 mm^3 was selected and mounted on a loop with paratone on a XtaLAB Synergy-S diffractometer. The crystal was kept at a steady T = 100(2) K during data collection. The structure was solved with the ShelXT 2018/2 (Sheldrick, 2018) solution program using dual methods and by using Olex2 1.5-alpha (Dolomanov et al., 2009) as the graphical interface. The model was refined with olex2.refine 1.5-alpha (Bourhis et al., 2015) using full matrix least squares minimisation on $\boldsymbol{F}^2$.

Crystal Data. $C_{13}H_{18}O_2S$, M_r = 238.353, triclinic, P-1 (No. 2), a = 7.0926(4) Å, b = 9.4062(5) Å, c = 9.6924(6) Å, α = 109.498(5)°, β = 93.863(5)°, γ = 94.139(5)°, V = 605.07(6) Å^3, T = 100(2) K, Z = 2, Z' = 1, μ (Mo K_α) = 0.250, 16054 reflections measured, 4178 unique (R_{int} = 0.0308) which were used in all calculations. The final wR_2 was 0.0777 (all data) and R_1 was 0.0430 (I≥2 σ(I)).

Table S7: Crystallographic data and structure refinement for **TC-C=O**.

**Compound**	**TC-C=O**
Formula	$C_{13}H_{18}O_2S$
$D_{calc.}$/ g cm^{-3}	1.308
μ/mm^{-1}	0.250
Formula Weight	238.353
Color	colorless
Shape	block-shaped
Size/mm^3	0.42×0.29×0.22
T/K	100(2)
Crystal System	triclinic
Space Group	P-1
a/Å	7.0926(4)
b/Å	9.4062(5)
c/Å	9.6924(6)
α/°	109.498(5)
β/°	93.863(5)

$\gamma/°$	94.139(5)
V/Å^3	605.07(6)
Z	2
Z'	1
Wavelength/Å	0.71073
Radiation type	Mo K_{α}
$\Theta_{min}/°$	3.48
$\Theta_{max}/°$	33.55
Measured Refl's.	16054
Indep't Refl's	4178
Refl's I≥2 σ(I)	4045
R_{int}	0.0308
Parameters	307
Restraints	282
Largest Peak	0.3286
Deepest Hole	-0.3333
GooF	1.0295
wR_2 (all data)	0.0777
wR_2	0.0770
R_1 (all data)	0.0447
R_1	0.0430

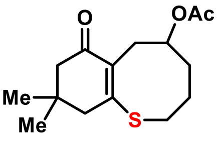

TC-OAc

≡

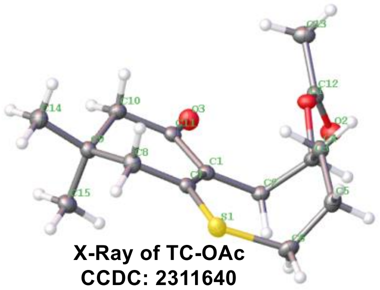

X-Ray of TC-OAc
CCDC: 2311640

Experimental. Single crystals of $C_{15}H_{22}O_3S$ **TC-OAc** were prepared by slow evaporation of hexane/ethyl acetate solution. Single colorless needle-shaped crystals of **TC-OAc** were chosen from the sample. A suitable crystal was selected and mounted on nylon loop with NVH oil on a Bruker D8 Venture diffractometer. The crystal was kept at 100(2) K during data collection. Using Olex2[2], the structure was solved with the SHELXT[3] structure solution program using Intrinsic Phasing and refined with the SHELXL[4] refinement package using Least Squares minimisation.

Crystal Data. $C_{15}H_{22}O_3S$, M_r = 282.38, triclinic, space group P-1 (No. 2), a = 6.2325(15) Å, b = 8.939(2) Å, c = 13.657(3) Å, α = 78.542(10)°, β = 84.029(8)°, γ = 84.356(8)°, V = 739.3(3) Å^3, T = 100.00 K, μ (Mo K_{α}) = 0.221 mm^{-1}, 24476 reflections measured, 3038 unique (R_{int} = 0.0426) which were used in all calculations. The final wR_2 was 0.0778 (all data) and R_1 was 0.0327 (I>2 σ(I)).

Table S8: Crystallographic data and structure refinement for **TC-OAc**.

Compound	TC-OAc
Formula	$C_{15}H_{22}O_3S$
$D_{calc.}$/ g cm^{-3}	1.268
μ/mm^{-1}	0.221
Formula Weight	282.38
Color	colorless
Shape	needle-shaped
Size/mm^3	0.199×0.162×0.14
T/K	100.00
Crystal System	triclinic
Space Group	P-1
a/Å	6.2325(15)
b/Å	8.939(2)
c/Å	13.657(3)
α/°	78.542(10)
β/°	84.029(8)
γ/°	84.356(8)
V/Å^3	739.3(3)
Z	2
Radiation type	Mo K_a
Θ_{min}/°	5.066
Θ_{max}/°	52.916
Measured Refl's.	24476
Indep't Refl's	3038
R_{int}	0.0426
Parameters	175
Restraints	0
Largest Peak	0.36
Deepest Hole	-0.26
GooF	1.063
wR_2 (all data)	0.0778
wR_2	0.0750
R_1 (all data)	0.0366
R_1	0.0327

13. Analysis of conformational and steric effects

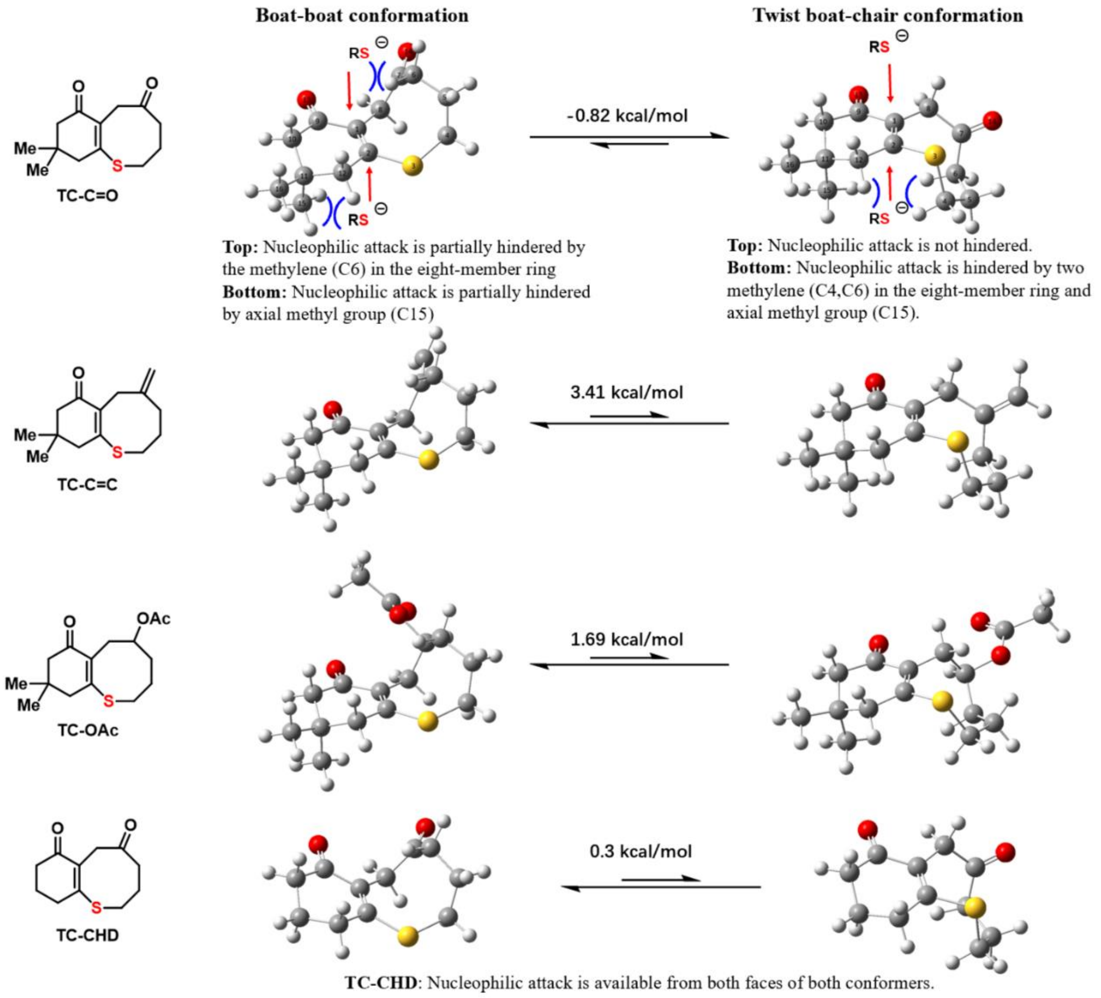

Figure S15. Analysis of conformational and steric effects.

The energy differences between the boat-boat conformations and twist boat-chair conformations of the **TC-C=O**, **TC-OAc**, **TC-C=C**, and **TC-CHD** monomers were calculated. The **TC-C=O** in the twist boat-chair conformation exhibits relatively lower energy compared to the boat-boat conformation. Conversely, both **TC-C=C** and **TC-OAc** show lower energy in the boat-boat conformation**.** These findings are consistent with single-crystal X-ray analysis. In monomers with two methyl groups, the twist boat-chair conformation provides an open space that facilitates nucleophilic attack from the top face, while the boat-boat conformation is partially hindered from both the top and bottom faces. However, both conformations in **TC-CHD** provide an easily accessible environment for nucleophilic attack. These analyses provide a rational explanation of the polymerization rate order **TC-CHD** > **TC-C=O** > **TC-OAc** > **TC-C=C**.

14. Computational details

Computational Details.

All computational calculations were conducted using the density functional theory (DFT) implemented in the Gaussian 09 software package.[5] Geometry optimizations and frequency calculations of intermediates (INT) and

transition states (TS) were carried out employing the B3LYP functional and the 6-31G* basis set.[6-8] To achieve high-precision Gibbs free energies, single-point calculations were subsequently performed using the M06-2X functional and the def2TZVPP basis set, incorporating the SMD continuum solvent model for THF.[9-10] All of the energies reported are free energies in the unit of kcal/mol.

TC-C=O-INT0

Standard orientation:

Center Number	Atomic Number	Atomic Type	Coordinates (Angstroms) X	Y	Z
1	6	0	-2.436527	-1.136213	-0.949480
2	6	0	-2.457453	-1.060887	0.588932
3	6	0	-1.512789	0.084317	1.019961
4	6	0	-0.206719	0.165876	0.256554
5	6	0	0.042287	-0.529270	-0.892045
6	6	0	-1.035965	-1.305507	-1.523908
7	16	0	1.081511	1.135283	1.053544
8	6	0	1.762186	-0.178085	2.197502
9	6	0	2.914260	-1.009416	1.623136
10	6	0	2.599690	-1.734447	0.298571
11	6	0	2.630375	-0.778889	-0.902105
12	6	0	1.343717	-0.443980	-1.645596
13	6	0	-2.006360	-2.405413	1.193934
14	6	0	-3.882732	-0.751151	1.079097
15	8	0	3.708632	-0.359235	-1.291460
16	1	0	-3.061754	-1.953887	-1.327509
17	1	0	-2.834168	-0.193085	-1.353361
18	1	0	-1.301379	0.002384	2.096188
19	1	0	-2.000123	1.055363	0.868371
20	1	0	2.106678	0.358296	3.090141
21	1	0	0.935211	-0.826665	2.513070
22	1	0	3.210236	-1.756122	2.376803
23	1	0	3.788239	-0.368302	1.453306
24	1	0	3.389769	-2.472558	0.113965
25	1	0	1.643431	-2.264380	0.366140
26	1	0	1.467516	0.564795	-2.059531
27	1	0	1.276691	-1.140655	-2.493498
28	1	0	-0.981101	-2.656238	0.901570
29	1	0	-2.043837	-2.370097	2.290707
30	1	0	-2.658159	-3.224237	0.861868
31	1	0	-3.908491	-0.650018	2.172509
32	1	0	-4.251532	0.186370	0.647342
33	1	0	-4.580124	-1.552836	0.800874

34	16	0	-0.804996	2.894452	-0.952398
35	6	0	-0.472957	4.383516	0.074754
36	1	0	-1.401973	4.876188	0.397237
37	1	0	0.089688	4.118119	0.980979
38	1	0	0.122229	5.128948	-0.471779
39	8	0	-0.848301	-2.033246	-2.503486

##

TC-C=O-INT1

Standard orientation:

Center Number	Atomic Number	Atomic Type	Coordinates (Angstroms) X	Y	Z
1	6	0	-2.119574	-1.622842	-0.810797
2	6	0	-2.633561	-0.570324	0.191901
3	6	0	-1.900207	0.765583	-0.071410
4	6	0	-0.372975	0.724894	-0.268459
5	6	0	0.137835	-0.420595	-0.985200
6	6	0	-0.619113	-1.611404	-1.159987
7	16	0	0.516587	0.927746	1.667056
8	6	0	1.032376	-0.790870	2.023161
9	6	0	2.507708	-1.140427	1.770842
10	6	0	2.796039	-1.586400	0.323024
11	6	0	2.727730	-0.449017	-0.697686
12	6	0	1.520957	-0.408222	-1.619798
13	6	0	-2.485382	-1.070403	1.641961
14	6	0	-4.140242	-0.326371	-0.046116
15	8	0	3.661808	0.333754	-0.795605
16	1	0	-2.371855	-2.637247	-0.475134
17	1	0	-2.641365	-1.492022	-1.772192
18	1	0	-2.156108	1.479721	0.720053
19	1	0	-2.306259	1.171876	-1.008369
20	1	0	0.774211	-0.980781	3.071872
21	1	0	0.414230	-1.452559	1.403689
22	1	0	2.783795	-1.971823	2.438934
23	1	0	3.155530	-0.293979	2.027131
24	1	0	3.821497	-1.976174	0.277112
25	1	0	2.105078	-2.387909	0.036047
26	1	0	1.674121	0.443699	-2.292066
27	1	0	1.559483	-1.333659	-2.214850
28	1	0	-1.468347	-1.381425	1.876240

29	1	0	-2.763857	-0.288309	2.359106
30	1	0	-3.144491	-1.934443	1.806693
31	1	0	-4.540279	0.415545	0.659173
32	1	0	-4.329759	0.040210	-1.062747
33	1	0	-4.711210	-1.255107	0.086848
34	16	0	0.150028	2.316831	-1.106963
35	6	0	-0.318568	3.632517	0.071302
36	1	0	-0.057071	4.577252	-0.416982
37	1	0	-1.390921	3.639645	0.290849
38	1	0	0.246821	3.547802	1.002674
39	8	0	-0.176459	-2.658784	-1.698397

###

TC-C=O-INT2

Standard orientation:

Center Number	Atomic Number	Atomic Type	Coordinates (Angstroms) X	Y	Z
1	6	0	-3.125941	-0.592386	0.505606
2	6	0	-2.647046	0.852174	0.279182
3	6	0	-1.694492	0.860506	-0.941060
4	6	0	-0.717703	-0.291354	-0.997800
5	6	0	-0.795112	-1.393003	-0.193969
6	6	0	-1.980361	-1.584521	0.662900
7	16	0	1.790696	2.557752	0.823497
8	6	0	3.139685	1.441196	1.423116
9	6	0	3.174153	0.041333	0.779771
10	6	0	1.914884	-0.778607	1.092620
11	6	0	1.716430	-1.993177	0.203956
12	6	0	0.277107	-2.456431	-0.118783
13	6	0	-1.933950	1.379052	1.540059
14	6	0	-3.851744	1.760169	-0.025300
15	8	0	2.650268	-2.664238	-0.204768
16	1	0	-3.764339	-0.674255	1.392417
17	1	0	-3.728783	-0.919968	-0.357187
18	1	0	-1.124773	1.796081	-0.939239
19	1	0	-2.285567	0.851645	-1.871797
20	1	0	4.115226	1.915084	1.239100
21	1	0	3.062170	1.321751	2.516257
22	1	0	4.067477	-0.513263	1.106943
23	1	0	3.258018	0.157786	-0.307593

24	1	0	1.951687	-1.150206	2.131289
25	1	0	1.054651	-0.107688	1.035699
26	1	0	0.346741	-3.055645	-1.034929
27	1	0	-0.023922	-3.147104	0.680731
28	1	0	-1.041137	0.800537	1.788661
29	1	0	-1.589250	2.408107	1.399626
30	1	0	-2.618602	1.350799	2.398742
31	1	0	-3.523828	2.787084	-0.229929
32	1	0	-4.415449	1.404688	-0.898102
33	1	0	-4.539450	1.794024	0.829234
34	16	0	0.525841	-0.268490	-2.256623
35	6	0	0.896339	1.501405	-2.538894
36	1	0	1.729471	1.499058	-3.248649
37	1	0	0.051723	2.031853	-2.987579
38	1	0	1.223392	1.958161	-1.585532
39	8	0	-2.088211	-2.537620	1.435196

##

TC-C=O-INT1-2

Standard orientation:

Center Number	Atomic Number	Atomic Type	Coordinates (Angstroms) X	Y	Z
1	6	0	-1.684879	-2.089743	0.085663
2	6	0	-2.558867	-0.846791	0.322342
3	6	0	-1.627465	0.254383	0.878348
4	6	0	-0.399131	0.595272	0.009101
5	6	0	0.151192	-0.526223	-0.749668
6	6	0	-0.384218	-1.834337	-0.695967
7	16	0	0.947887	1.401123	1.228080
8	6	0	1.539311	-0.107377	2.095848
9	6	0	2.910417	-0.628396	1.643217
10	6	0	2.932688	-1.301867	0.256424
11	6	0	2.731037	-0.317192	-0.900482
12	6	0	1.384130	-0.335723	-1.609484
13	6	0	-3.635408	-1.150520	1.382745
14	6	0	-3.266939	-0.435553	-0.982103
15	8	0	3.661850	0.385760	-1.269078
16	1	0	-1.418433	-2.540979	1.055689
17	1	0	-2.237035	-2.864721	-0.462440
18	1	0	-1.266188	-0.102706	1.851755

19	1	0	-2.197172	1.167155	1.090732
20	1	0	1.569498	0.130354	3.166065
21	1	0	0.783954	-0.888620	1.947894
22	1	0	3.251358	-1.364412	2.389361
23	1	0	3.640923	0.190668	1.650570
24	1	0	3.928891	-1.740508	0.113015
25	1	0	2.187832	-2.102218	0.198495
26	1	0	1.346278	0.560880	-2.237572
27	1	0	1.422293	-1.220240	-2.265352
28	1	0	-3.182849	-1.442583	2.339472
29	1	0	-4.274752	-0.275656	1.567313
30	1	0	-4.284006	-1.974511	1.055892
31	1	0	-3.840117	0.490890	-0.850432
32	1	0	-2.549018	-0.270918	-1.788036
33	1	0	-3.964029	-1.225827	-1.294671
34	16	0	-0.800847	2.036925	-1.183726
35	6	0	-1.114283	3.501923	-0.125965
36	1	0	-1.493845	4.283940	-0.793042
37	1	0	-1.871963	3.302308	0.639063
38	1	0	-0.203009	3.860322	0.359072
39	8	0	0.142305	-2.841825	-1.243750

##

TC-C=O-INT2-2

Standard orientation:

Center Number	Atomic Number	Atomic Type	Coordinates (Angstroms) X	Y	Z
1	6	0	-1.854457	-1.040976	1.333910
2	6	0	-2.650286	-0.364868	0.204055
3	6	0	-1.660970	0.456952	-0.651404
4	6	0	-0.400775	-0.276362	-1.025629
5	6	0	0.112408	-1.313270	-0.302035
6	6	0	-0.639631	-1.825849	0.862767
7	16	0	0.227775	3.123522	1.172529
8	6	0	1.159681	1.706138	1.900743
9	6	0	2.141034	1.034958	0.916987
10	6	0	2.536666	-0.432061	1.291840
11	6	0	2.704790	-1.287729	0.050401
12	6	0	1.453480	-1.962130	-0.566165
13	6	0	-3.694327	0.591939	0.804673

14	6	0	-3.362525	-1.429125	-0.654634
15	8	0	3.794031	-1.491268	-0.460661
16	1	0	-1.474331	-0.262792	2.012734
17	1	0	-2.481002	-1.714483	1.930693
18	1	0	-1.338308	1.362379	-0.090950
19	1	0	-2.173768	0.810834	-1.554379
20	1	0	1.712844	2.029966	2.795454
21	1	0	0.454728	0.937017	2.253323
22	1	0	3.049779	1.640253	0.814710
23	1	0	1.677793	1.040882	-0.069739
24	1	0	3.480343	-0.457905	1.847456
25	1	0	1.755830	-0.868622	1.926548
26	1	0	1.663038	-2.101713	-1.630325
27	1	0	1.420445	-2.959332	-0.106762
28	1	0	-3.207050	1.374236	1.397153
29	1	0	-4.274665	1.083078	0.012377
30	1	0	-4.399636	0.050442	1.449295
31	1	0	-3.944488	-0.955758	-1.455709
32	1	0	-2.649767	-2.117759	-1.122034
33	1	0	-4.054548	-2.024413	-0.044554
34	16	0	0.483089	0.297709	-2.458114
35	6	0	0.031504	2.075383	-2.546717
36	1	0	0.747218	2.512930	-3.248815
37	1	0	-0.981531	2.222840	-2.930298
38	1	0	0.151545	2.539596	-1.555533
39	8	0	-0.265010	-2.818655	1.488906

##

TC-C=O-TS1

Standard orientation:

Center Number	Atomic Number	Atomic Type	Coordinates (Angstroms) X	Y	Z
1	6	0	-2.153904	-1.549858	-0.936403
2	6	0	-2.540699	-0.773332	0.331508
3	6	0	-1.874845	0.622465	0.256919
4	6	0	-0.379327	0.708700	-0.148470
5	6	0	0.122752	-0.394341	-0.957229
6	6	0	-0.658870	-1.525536	-1.301106
7	16	0	0.761811	1.107837	1.447709
8	6	0	1.223365	-0.529089	2.140492

9	6	0	2.613248	-1.054886	1.762025
10	6	0	2.715484	-1.585277	0.319496
11	6	0	2.723117	-0.467024	-0.725619
12	6	0	1.494639	-0.312845	-1.602045
13	6	0	-2.160989	-1.588219	1.582494
14	6	0	-4.068059	-0.551078	0.374604
15	8	0	3.733371	0.206296	-0.877417
16	1	0	-2.456846	-2.602360	-0.861360
17	1	0	-2.700576	-1.132677	-1.797243
18	1	0	-2.029938	1.156275	1.201257
19	1	0	-2.428726	1.175844	-0.511742
20	1	0	1.147361	-0.426846	3.230004
21	1	0	0.471801	-1.254744	1.826729
22	1	0	2.859434	-1.876966	2.453714
23	1	0	3.371401	-0.275705	1.909203
24	1	0	3.673024	-2.111459	0.212419
25	1	0	1.904141	-2.291631	0.112526
26	1	0	1.645981	0.606130	-2.181487
27	1	0	1.522038	-1.167199	-2.298310
28	1	0	-1.160652	-2.014842	1.491982
29	1	0	-2.195740	-0.976862	2.493226
30	1	0	-2.857343	-2.427950	1.711923
31	1	0	-4.368477	-0.007547	1.281790
32	1	0	-4.412763	0.026894	-0.491817
33	1	0	-4.600410	-1.511530	0.369557
34	16	0	-0.181101	2.366502	-1.127507
35	6	0	-0.739805	3.674367	0.029150
36	1	0	-0.731614	4.611103	-0.539059
37	1	0	-1.758570	3.497877	0.389971
38	1	0	-0.066621	3.776010	0.884194
39	8	0	-0.235234	-2.506985	-1.968283

##

TC-C=O-TS2

Standard orientation:

Center Number	Atomic Number	Atomic Type	Coordinates (Angstroms) X	Y	Z
1	6	0	-2.148919	-1.608618	-0.804474
2	6	0	-2.646167	-0.543387	0.192310
3	6	0	-1.905292	0.785477	-0.087560

4	6	0	-0.388030	0.724657	-0.319958
5	6	0	0.119159	-0.430182	-0.996614
6	6	0	-0.646607	-1.626559	-1.138448
7	16	0	0.531973	0.933092	1.724384
8	6	0	1.055858	-0.789578	2.037403
9	6	0	2.530655	-1.130028	1.762467
10	6	0	2.799248	-1.583729	0.312821
11	6	0	2.712196	-0.458968	-0.719703
12	6	0	1.499536	-0.440023	-1.637678
13	6	0	-2.483131	-1.033195	1.643878
14	6	0	-4.153336	-0.293153	-0.033571
15	8	0	3.639499	0.327770	-0.840814
16	1	0	-2.425178	-2.616867	-0.469822
17	1	0	-2.658878	-1.466147	-1.770740
18	1	0	-2.133066	1.500454	0.710774
19	1	0	-2.328702	1.200314	-1.014309
20	1	0	0.809941	-1.010611	3.083303
21	1	0	0.435211	-1.445035	1.411323
22	1	0	2.827392	-1.956186	2.428374
23	1	0	3.174821	-0.276423	2.002717
24	1	0	3.825664	-1.969522	0.253709
25	1	0	2.108794	-2.391897	0.043643
26	1	0	1.649462	0.395143	-2.330965
27	1	0	1.535979	-1.378711	-2.210841
28	1	0	-1.461500	-1.334326	1.870278
29	1	0	-2.758649	-0.247546	2.357824
30	1	0	-3.136392	-1.899676	1.819356
31	1	0	-4.540405	0.460594	0.666135
32	1	0	-4.351563	0.061406	-1.052896
33	1	0	-4.729121	-1.216016	0.118035
34	16	0	0.178723	2.296209	-1.127093
35	6	0	-0.280435	3.611363	0.052785
36	1	0	0.016954	4.552817	-0.420943
37	1	0	-1.356431	3.644921	0.250001
38	1	0	0.264543	3.497755	0.992902
39	8	0	-0.205551	-2.687484	-1.643744

##

TC-C=O-TS1-2

Standard orientation:

Center Atomic Atomic Coordinates (Angstroms)

Number	Number	Type	X	Y	Z
1	6	0	-2.038513	-1.766146	-0.551351
2	6	0	-2.491205	-0.838965	0.606979
3	6	0	-1.380576	0.181318	1.002910
4	6	0	-0.292355	0.493888	-0.034768
5	6	0	0.251096	-0.672490	-0.717011
6	6	0	-0.543556	-1.829850	-0.911107
7	16	0	1.015703	1.598879	0.781485
8	6	0	1.759983	0.528334	2.092094
9	6	0	3.084359	-0.153826	1.730268
10	6	0	2.983143	-1.183531	0.590734
11	6	0	2.855368	-0.499775	-0.774227
12	6	0	1.535603	-0.608057	-1.512769
13	6	0	-2.798260	-1.698707	1.851956
14	6	0	-3.779684	-0.094176	0.203216
15	8	0	3.833410	0.061122	-1.251542
16	1	0	-2.371337	-2.796095	-0.372498
17	1	0	-2.536041	-1.447590	-1.478661
18	1	0	-0.859200	-0.229143	1.875422
19	1	0	-1.842731	1.113844	1.348450
20	1	0	1.908700	1.183999	2.959023
21	1	0	1.022744	-0.226457	2.384260
22	1	0	3.458647	-0.653839	2.638156
23	1	0	3.832515	0.598584	1.450507
24	1	0	3.913943	-1.764360	0.564067
25	1	0	2.142446	-1.864047	0.760027
26	1	0	1.541422	0.189881	-2.268517
27	1	0	1.577318	-1.570510	-2.050601
28	1	0	-1.930864	-2.313354	2.123579
29	1	0	-3.053884	-1.073157	2.719063
30	1	0	-3.643029	-2.374900	1.664509
31	1	0	-4.170032	0.508539	1.036183
32	1	0	-3.585645	0.572815	-0.642735
33	1	0	-4.563763	-0.805926	-0.090418
34	16	0	-1.024189	1.770599	-1.401114
35	6	0	-1.624195	3.280667	-0.537394
36	1	0	-2.050186	3.940983	-1.302249
37	1	0	-2.408854	3.050348	0.191810
38	1	0	-0.810579	3.806911	-0.031497
39	8	0	-0.153792	-2.884907	-1.473582

##

TC-C=O-TS2-2

Standard orientation:

Center Number	Atomic Number	Atomic Type	Coordinates (Angstroms) X	Y	Z
1	6	0	-1.869800	-1.933318	0.267658
2	6	0	-2.705877	-0.643092	0.320562
3	6	0	-1.731541	0.492368	0.716526
4	6	0	-0.537753	0.626344	-0.223645
5	6	0	0.001201	-0.530109	-0.801222
6	6	0	-0.576530	-1.831859	-0.552892
7	16	0	1.207029	1.375676	1.607831
8	6	0	1.618530	-0.338983	2.088760
9	6	0	2.935497	-0.891844	1.513170
10	6	0	2.835670	-1.484841	0.091320
11	6	0	2.581535	-0.474853	-1.028305
12	6	0	1.210761	-0.492397	-1.714702
13	6	0	-3.795966	-0.764465	1.400650
14	6	0	-3.382790	-0.374248	-1.036423
15	8	0	3.489241	0.222605	-1.452065
16	1	0	-1.590364	-2.222396	1.292539
17	1	0	-2.442974	-2.774355	-0.143530
18	1	0	-1.354925	0.285285	1.723057
19	1	0	-2.272931	1.443048	0.782946
20	1	0	1.662493	-0.387930	3.185194
21	1	0	0.794388	-1.004950	1.781014
22	1	0	3.297731	-1.700346	2.170338
23	1	0	3.697919	-0.104320	1.522466
24	1	0	3.803063	-1.947001	-0.148694
25	1	0	2.071453	-2.268340	0.062895
26	1	0	1.198518	0.352783	-2.405851
27	1	0	1.195043	-1.423121	-2.300459
28	1	0	-3.357102	-0.935734	2.391612
29	1	0	-4.402660	0.149532	1.455991
30	1	0	-4.472475	-1.602101	1.183180
31	1	0	-3.946176	0.567376	-1.013649
32	1	0	-2.647473	-0.303300	-1.842441
33	1	0	-4.084436	-1.182638	-1.283623
34	16	0	-0.502979	2.105720	-1.267731
35	6	0	-0.554601	3.518147	-0.116816
36	1	0	-0.697327	4.406946	-0.740901
37	1	0	-1.384747	3.443752	0.591065

38	1	0	0.385860	3.591881	0.431529
39	8	0	-0.100204	-2.891044	-1.008449

##

TP-C=O-INT0

Standard orientation:

Center Number	Atomic Number	Atomic Type	Coordinates (Angstroms) X	Y	Z
1	6	0	3.331660	-0.809063	-0.298571
2	6	0	2.516277	0.026961	-1.285843
3	16	0	1.052467	-0.870527	-1.946019
4	6	0	-0.184369	-0.590529	-0.690287
5	6	0	0.034958	-0.546139	0.647338
6	6	0	1.356035	-0.671242	1.384566
7	6	0	2.467872	-1.494617	0.756657
8	8	0	2.717170	-2.630400	1.124424
9	6	0	-1.535414	-0.327305	-1.321681
10	6	0	-2.744283	-0.489976	-0.379797
11	6	0	-2.396031	0.240474	0.930025
12	6	0	-1.110076	-0.262763	1.559475
13	8	0	-1.021973	-0.417777	2.773948
14	6	0	-3.035881	-1.980630	-0.116038
15	6	0	-3.980925	0.157852	-1.025401
16	1	0	4.036839	-0.145290	0.228117
17	1	0	3.918173	-1.586299	-0.799736
18	1	0	2.134215	0.969431	-0.856478
19	1	0	3.116299	0.275536	-2.167882
20	1	0	1.706821	0.363900	1.532515
21	1	0	1.137997	-1.099598	2.365972
22	1	0	-1.490780	0.712464	-1.678835
23	1	0	-1.661588	-0.961500	-2.211510
24	1	0	-3.196837	0.153341	1.673486
25	1	0	-2.224517	1.306617	0.717512
26	1	0	-3.312827	-2.493381	-1.046735
27	1	0	-3.867283	-2.096609	0.591176
28	1	0	-2.164705	-2.494960	0.302640
29	1	0	-4.218583	-0.317007	-1.987128
30	1	0	-4.860326	0.056252	-0.375990
31	1	0	-3.815107	1.226031	-1.206727
32	16	0	0.495917	2.700353	0.131633

33	6	0	0.873164	4.501819	0.194783
34	1	0	0.700749	4.993742	-0.774439
35	1	0	0.247174	5.022222	0.934772
36	1	0	1.920645	4.694256	0.471099

###

TP-C=O-INT1

Standard orientation:

Center Number	Atomic Number	Atomic Type	Coordinates (Angstroms) X	Y	Z
1	6	0	-2.593346	-0.433266	1.361284
2	6	0	-2.112937	1.031549	1.436335
3	16	0	-0.304338	1.303154	1.539318
4	6	0	0.316700	0.627964	-0.262047
5	6	0	-0.334078	-0.592379	-0.720425
6	6	0	-1.794461	-0.618007	-1.125512
7	6	0	-2.815730	-0.976946	-0.052226
8	8	0	-3.814513	-1.637703	-0.292856
9	6	0	1.847364	0.535546	-0.100779
10	6	0	2.407363	-0.826845	0.369027
11	6	0	1.855747	-1.897645	-0.585248
12	6	0	0.337325	-1.836707	-0.829856
13	8	0	-0.216058	-2.904763	-1.191201
14	6	0	2.033588	-1.174313	1.822137
15	6	0	3.945019	-0.767903	0.270822
16	1	0	-3.547870	-0.549881	1.889572
17	1	0	-1.860833	-1.080832	1.859658
18	1	0	-2.515534	1.619318	0.602677
19	1	0	-2.498163	1.486947	2.355871
20	1	0	-2.111930	0.367739	-1.502005
21	1	0	-1.940707	-1.344150	-1.930437
22	1	0	2.281084	0.736661	-1.089207
23	1	0	2.204190	1.335837	0.562984
24	1	0	2.087035	-2.906777	-0.220293
25	1	0	2.351598	-1.803859	-1.564929
26	1	0	2.354797	-0.389084	2.518340
27	1	0	2.523586	-2.112807	2.117104
28	1	0	0.955389	-1.296022	1.939166
29	1	0	4.361385	-0.010881	0.950655
30	1	0	4.388713	-1.736193	0.538383

31	1	0	4.271959	-0.521050	-0.747675
32	16	0	-0.070466	2.029720	-1.500257
33	6	0	0.663346	3.538253	-0.770385
34	1	0	0.286911	3.688867	0.244847
35	1	0	1.757920	3.496989	-0.751249
36	1	0	0.356494	4.377124	-1.404746

##

TP-C=O-INT2

Standard orientation:

Center Number	Atomic Number	Atomic Type	Coordinates (Angstroms) X	Y	Z
1	6	0	-2.188598	-0.754375	1.091706
2	6	0	-2.519095	0.758027	0.874405
3	16	0	-1.627877	1.893803	2.017828
4	6	0	0.623708	0.341357	-1.062703
5	6	0	0.079838	-0.894928	-0.883103
6	6	0	-1.302830	-1.326076	-1.312040
7	6	0	-2.342548	-1.554361	-0.181180
8	8	0	-3.287969	-2.292820	-0.405452
9	6	0	1.999717	0.730944	-0.561810
10	6	0	2.591959	-0.179504	0.537363
11	6	0	2.324906	-1.641894	0.136779
12	6	0	0.868636	-1.938856	-0.189745
13	8	0	0.381779	-3.036729	0.070433
14	6	0	1.959543	0.148667	1.905069
15	6	0	4.111706	0.054023	0.620834
16	1	0	-2.841297	-1.182159	1.858889
17	1	0	-1.157252	-0.827705	1.444237
18	1	0	-2.283191	1.027186	-0.163620
19	1	0	-3.607404	0.873091	0.971109
20	1	0	-1.740779	-0.576312	-1.978340
21	1	0	-1.255833	-2.270660	-1.862468
22	1	0	2.673457	0.744784	-1.434442
23	1	0	1.968968	1.761443	-0.190690
24	1	0	2.643963	-2.342776	0.916240
25	1	0	2.909669	-1.880774	-0.767132
26	1	0	2.317611	1.122741	2.261226
27	1	0	2.242926	-0.604576	2.651948
28	1	0	0.866600	0.227349	1.872950

29	1	0	4.333395	1.104481	0.848800
30	1	0	4.556305	-0.558935	1.415318
31	1	0	4.613689	-0.200519	-0.322226
32	16	0	-0.182658	1.549086	-2.104368
33	6	0	-0.133807	3.084080	-1.094187
34	1	0	-0.496733	2.864542	-0.072954
35	1	0	0.862804	3.535347	-1.084217
36	1	0	-0.827641	3.770990	-1.588132

##

TP-C=O-INT3

Standard orientation:

Center Number	Atomic Number	Atomic Type	Coordinates (Angstroms) X	Y	Z
1	6	0	-2.102654	0.394160	-1.236482
2	6	0	-1.902328	-0.843113	-0.372052
3	16	0	-0.292780	-1.708128	-0.634234
4	6	0	0.974614	-0.666623	0.015010
5	6	0	0.885431	0.641929	0.399987
6	6	0	-0.337328	1.543146	0.275893
7	6	0	-1.032008	1.442494	-1.087314
8	8	0	-0.722064	2.197341	-1.996391
9	6	0	2.264425	-1.465772	0.129085
10	6	0	3.548608	-0.615337	0.111052
11	6	0	3.358183	0.492546	1.158051
12	6	0	2.077569	1.304904	0.980050
13	8	0	2.058875	2.474909	1.355608
14	6	0	3.787122	-0.010380	-1.286835
15	6	0	4.751825	-1.496893	0.486922
16	1	0	-3.093781	0.781940	-0.906540
17	1	0	-2.198119	0.140888	-2.297241
18	1	0	-2.004193	-0.625144	0.691502
19	1	0	-2.683956	-1.574937	-0.597313
20	1	0	-1.052326	1.338680	1.082736
21	1	0	0.014197	2.569101	0.399650
22	1	0	2.226558	-2.041874	1.066583
23	1	0	2.299032	-2.211649	-0.676256
24	1	0	4.195048	1.200058	1.166701
25	1	0	3.317841	0.038120	2.160840
26	1	0	3.929884	-0.802877	-2.032005

27	1	0	4.687632	0.616343	-1.286642
28	1	0	2.945991	0.608255	-1.613437
29	1	0	4.883242	-2.313411	-0.234775
30	1	0	5.678265	-0.909028	0.498467
31	1	0	4.625557	-1.944257	1.480848
32	6	0	-5.226635	-0.408268	1.886593
33	1	0	-4.378318	-0.998256	2.269047
34	1	0	-6.138461	-0.868069	2.297150
35	1	0	-5.146483	0.593478	2.335413
36	16	0	-5.274910	-0.345129	0.044345

##

TP-C=O-INT4

Standard orientation:

Center Number	Atomic Number	Atomic Type	Coordinates (Angstroms) X	Y	Z
1	6	0	-2.575298	1.445414	-0.132275
2	6	0	-3.220699	0.067518	-0.328437
3	16	0	-0.130675	-1.911987	0.770609
4	6	0	1.204754	-0.839162	0.598553
5	6	0	1.127266	0.546299	0.692457
6	6	0	-0.192268	1.236338	0.892242
7	6	0	-1.057958	1.325601	-0.358046
8	8	0	-0.619348	1.336796	-1.493926
9	6	0	2.567875	-1.481480	0.358821
10	6	0	3.573545	-0.593416	-0.404465
11	6	0	3.651556	0.749476	0.339424
12	6	0	2.289663	1.402120	0.599999
13	8	0	2.252049	2.635235	0.757477
14	6	0	3.106861	-0.375440	-1.858730
15	6	0	4.954381	-1.271719	-0.419532
16	1	0	-2.789649	1.818261	0.876166
17	1	0	-2.976688	2.155051	-0.864784
18	1	0	-2.775939	-0.658557	0.359545
19	1	0	-3.034784	-0.277889	-1.350264
20	1	0	-0.779176	0.763837	1.688586
21	1	0	0.010498	2.277986	1.188269
22	1	0	3.001676	-1.745925	1.337456
23	1	0	2.416128	-2.429320	-0.168917
24	1	0	4.268066	1.480738	-0.198907

25	1	0	4.136151	0.593550	1.317267
26	1	0	3.041432	-1.333243	-2.391518
27	1	0	3.817364	0.263277	-2.401297
28	1	0	2.122325	0.100008	-1.895162
29	1	0	4.912028	-2.241742	-0.933422
30	1	0	5.695409	-0.649931	-0.940557
31	1	0	5.323750	-1.449361	0.599160
32	6	0	-5.445709	-1.618305	-0.195101
33	1	0	-5.203061	-1.996952	-1.193329
34	1	0	-6.521441	-1.726413	-0.027512
35	1	0	-4.906757	-2.207308	0.553296
36	16	0	-5.039497	0.157502	-0.043855

##

TP-C=O-INT5

Standard orientation:

Center Number	Atomic Number	Atomic Type	Coordinates (Angstroms) X	Y	Z
1	6	0	-2.440746	-0.766649	-0.265244
2	6	0	-3.501335	0.235502	0.197549
3	6	0	1.282189	0.515439	-0.351122
4	6	0	1.346748	-0.833588	-0.229219
5	6	0	0.016317	-1.531950	-0.145545
6	6	0	-1.047720	-0.506212	0.309889
7	8	0	-1.178835	-0.469940	1.708052
8	6	0	2.480066	1.406640	-0.528256
9	6	0	3.785388	0.757288	0.006618
10	6	0	3.868434	-0.698547	-0.509226
11	6	0	2.626089	-1.547492	-0.242576
12	8	0	2.694170	-2.763333	-0.095817
13	6	0	3.795249	0.775075	1.548595
14	6	0	4.992392	1.559518	-0.508601
15	1	0	-2.375985	-0.772953	-1.357865
16	1	0	-2.731885	-1.776162	0.053300
17	1	0	-3.242218	1.249542	-0.130430
18	1	0	-3.568341	0.233099	1.288773
19	1	0	-0.252050	-1.935210	-1.131847
20	1	0	0.026060	-2.377425	0.550349
21	1	0	2.592179	1.625201	-1.601363
22	1	0	2.311429	2.373782	-0.038167

23	1	0	4.732167	-1.222298	-0.085426
24	1	0	4.013087	-0.688119	-1.601030
25	1	0	3.756756	1.803624	1.926677
26	1	0	4.710664	0.310490	1.932643
27	1	0	2.944353	0.229413	1.969869
28	1	0	4.946903	2.601283	-0.167631
29	1	0	5.931824	1.129638	-0.141610
30	1	0	5.031514	1.565363	-1.604541
31	6	0	-6.164947	1.078678	0.238586
32	1	0	-6.144793	1.016103	1.330965
33	1	0	-7.189287	0.912118	-0.104836
34	1	0	-5.844986	2.075985	-0.078927
35	16	0	-5.130193	-0.222548	-0.517039
36	16	0	-0.351361	1.172917	-0.303460
37	1	0	-0.293938	-0.355423	2.092515

##

TP-C=O-INT6

Standard orientation:

Center Number	Atomic Number	Atomic Type	Coordinates (Angstroms) X	Y	Z
1	6	0	-2.619844	0.390430	-0.585575
2	6	0	-3.663367	-0.075971	0.425055
3	6	0	2.274597	1.109123	-0.124172
4	6	0	1.195082	0.490507	-0.659460
5	6	0	-0.151660	1.120285	-0.895584
6	6	0	-1.226194	0.496983	0.011344
7	8	0	-0.970735	0.137321	1.144001
8	6	0	3.576326	0.388833	0.158638
9	6	0	3.421782	-1.141553	0.321097
10	6	0	2.593759	-1.659470	-0.870846
11	6	0	1.264617	-0.941291	-1.029038
12	8	0	0.277248	-1.512148	-1.481725
13	6	0	2.712341	-1.473125	1.650488
14	6	0	4.815719	-1.791043	0.313467
15	1	0	-2.895345	1.361265	-1.022334
16	1	0	-2.548691	-0.309942	-1.429579
17	1	0	-3.705387	0.615184	1.273259
18	1	0	-3.391645	-1.062220	0.812748
19	1	0	-0.142173	2.196320	-0.679865

20	1	0	-0.450948	1.006236	-1.943008
21	1	0	4.278069	0.606031	-0.661732
22	1	0	4.034054	0.802270	1.066676
23	1	0	2.390368	-2.732437	-0.790926
24	1	0	3.161106	-1.510892	-1.803174
25	1	0	3.304618	-1.118325	2.502825
26	1	0	2.592967	-2.557543	1.757924
27	1	0	1.718960	-1.017696	1.715533
28	1	0	5.430094	-1.415345	1.141090
29	1	0	4.736511	-2.878571	0.425888
30	1	0	5.350306	-1.588389	-0.622796
31	6	0	-6.308907	-0.728184	1.040650
32	1	0	-5.968711	-1.705070	1.397899
33	1	0	-7.340880	-0.820453	0.691970
34	1	0	-6.277532	-0.005712	1.862219
35	16	0	-5.311107	-0.159091	-0.379342
36	16	0	2.219757	2.852392	0.232242
37	1	0	3.428254	2.927947	0.824391

##

TP-C=O-INT7

Standard orientation:

Center Number	Atomic Number	Atomic Type	Coordinates (Angstroms) X	Y	Z
1	6	0	-2.352511	0.160295	-0.674316
2	6	0	-3.434943	-0.472958	0.195848
3	6	0	1.726575	1.143446	-0.577306
4	6	0	1.192021	-0.145395	-0.946944
5	6	0	-0.053726	-0.245223	-1.822245
6	6	0	-1.249286	-0.794858	-1.060574
7	8	0	-1.288421	-1.988479	-0.765671
8	6	0	3.092129	1.169609	0.093663
9	6	0	3.405457	-0.038660	1.000909
10	6	0	3.151529	-1.315868	0.175385
11	6	0	1.825764	-1.298991	-0.530523
12	8	0	1.340981	-2.524339	-0.740388
13	6	0	2.510625	-0.018978	2.255587
14	6	0	4.879820	0.010811	1.433872
15	1	0	-1.885051	1.027657	-0.186134
16	1	0	-2.778911	0.562264	-1.606208

17	1	0	-3.003044	-0.826575	1.137966
18	1	0	-3.871964	-1.337242	-0.314005
19	1	0	-0.269490	0.744593	-2.228108
20	1	0	0.139957	-0.935323	-2.655246
21	1	0	3.837853	1.204944	-0.716930
22	1	0	3.196028	2.107513	0.645950
23	1	0	3.197782	-2.214105	0.801354
24	1	0	3.939364	-1.426060	-0.586070
25	1	0	2.680119	0.896796	2.833244
26	1	0	2.735654	-0.872797	2.905728
27	1	0	1.446423	-0.059628	2.001595
28	1	0	5.085253	0.918661	2.013091
29	1	0	5.132676	-0.850757	2.063883
30	1	0	5.553333	0.004606	0.568225
31	6	0	-5.862617	-0.263119	1.556280
32	1	0	-6.233256	-1.123114	0.989936
33	1	0	-6.710886	0.367418	1.834862
34	1	0	-5.366297	-0.608929	2.468234
35	16	0	-4.743559	0.765118	0.543393
36	16	0	0.956002	2.589766	-0.891275
37	1	0	0.369849	-2.496177	-0.954596

###

TP-C=O-INT8

Standard orientation:

Center Number	Atomic Number	Atomic Type	Coordinates (Angstroms) X	Y	Z
1	6	0	-2.368230	0.836567	-0.117265
2	6	0	-3.451420	-0.193723	0.217823
3	6	0	2.539213	1.037352	-0.416299
4	6	0	1.198355	0.722065	-0.048641
5	6	0	0.060659	1.648306	0.294473
6	6	0	-1.072042	0.670546	0.680594
7	8	0	-1.250726	0.745574	2.062473
8	6	0	3.398304	-0.157259	-0.817032
9	6	0	3.076132	-1.488833	-0.086884
10	6	0	1.558904	-1.789672	-0.224044
11	6	0	0.754399	-0.562706	0.032575
12	8	0	-0.542834	-0.686503	0.367268
13	6	0	3.461512	-1.384205	1.402309

14	6	0	3.878594	-2.628775	-0.735650
15	1	0	-2.742421	1.849551	0.072500
16	1	0	-2.122262	0.774427	-1.183184
17	1	0	-3.760009	-0.110307	1.268218
18	1	0	-3.076544	-1.207577	0.044167
19	1	0	0.287654	2.303137	1.140644
20	1	0	-0.217504	2.287506	-0.551245
21	1	0	3.255252	-0.303771	-1.899341
22	1	0	4.451959	0.103451	-0.680707
23	1	0	1.249110	-2.587888	0.462176
24	1	0	1.335798	-2.151659	-1.239708
25	1	0	4.528897	-1.158671	1.506963
26	1	0	3.267139	-2.330401	1.921980
27	1	0	2.902904	-0.595008	1.915395
28	1	0	4.955798	-2.446846	-0.646245
29	1	0	3.661493	-3.588283	-0.250132
30	1	0	3.642798	-2.729620	-1.802090
31	6	0	-6.003242	-1.244286	-0.236304
32	1	0	-5.550926	-2.225455	-0.410501
33	1	0	-6.935224	-1.181248	-0.803976
34	1	0	-6.231873	-1.126316	0.827534
35	16	0	-4.923939	0.100292	-0.838047
36	16	0	3.121592	2.592481	-0.482640
37	1	0	-1.902829	0.075797	2.326683

##

TP-C=O-TS1

Standard orientation:

Center Number	Atomic Number	Atomic Type	Coordinates (Angstroms) X	Y	Z
1	6	0	3.119012	-0.771209	-0.843161
2	6	0	2.650804	0.683660	-0.870286
3	16	0	0.940496	0.939478	-1.467200
4	6	0	-0.119743	0.533808	0.013218
5	6	0	0.071046	-0.702749	0.698714
6	6	0	1.447942	-1.187862	1.133303
7	6	0	2.271572	-1.748486	-0.014246
8	8	0	2.313550	-2.936961	-0.288975
9	6	0	-1.562885	0.921565	-0.356457
10	6	0	-2.511083	-0.276206	-0.581328

11	6	0	-2.446075	-1.121506	0.700812
12	6	0	-1.026202	-1.506346	1.144994
13	8	0	-0.915556	-2.523826	1.862090
14	6	0	-2.109909	-1.110738	-1.812098
15	6	0	-3.939996	0.258406	-0.791531
16	1	0	4.135108	-0.804755	-0.413171
17	1	0	3.195521	-1.184273	-1.855017
18	1	0	2.709397	1.159634	0.114469
19	1	0	3.281119	1.263585	-1.554785
20	1	0	2.005315	-0.377767	1.627166
21	1	0	1.274938	-2.002094	1.842290
22	1	0	-1.968458	1.515440	0.469379
23	1	0	-1.562982	1.578038	-1.237557
24	1	0	-3.009911	-2.057175	0.594632
25	1	0	-2.920157	-0.564418	1.524528
26	1	0	-2.134982	-0.500770	-2.725040
27	1	0	-2.804966	-1.950417	-1.948726
28	1	0	-1.100799	-1.515472	-1.702509
29	1	0	-4.006212	0.877238	-1.697271
30	1	0	-4.654019	-0.569044	-0.899933
31	1	0	-4.265753	0.871611	0.058566
32	16	0	0.461328	2.156292	1.485339
33	6	0	0.354231	3.730931	0.552549
34	1	0	0.840406	3.628260	-0.423944
35	1	0	-0.684894	4.044410	0.390358
36	1	0	0.865918	4.522352	1.114514

##

TP-C=O-TS2

Standard orientation:

Center Number	Atomic Number	Atomic Type	Coordinates (Angstroms) X	Y	Z
1	6	0	-2.533549	-0.487054	1.339573
2	6	0	-2.114954	0.999432	1.464989
3	16	0	-0.320289	1.340414	1.626253
4	6	0	0.343105	0.613963	-0.370439
5	6	0	-0.327876	-0.586869	-0.768132
6	6	0	-1.787401	-0.611611	-1.170131
7	6	0	-2.807234	-0.960619	-0.086299
8	8	0	-3.844336	-1.549479	-0.349947

9	6	0	1.862938	0.529108	-0.175849
10	6	0	2.419761	-0.822658	0.330637
11	6	0	1.830750	-1.933014	-0.554244
12	6	0	0.309672	-1.863128	-0.755640
13	8	0	-0.288397	-2.934725	-1.002523
14	6	0	2.085106	-1.098944	1.808030
15	6	0	3.954396	-0.790750	0.184897
16	1	0	-3.452602	-0.677907	1.907152
17	1	0	-1.743071	-1.119850	1.761860
18	1	0	-2.518709	1.584423	0.628954
19	1	0	-2.563516	1.412739	2.375419
20	1	0	-2.102136	0.372669	-1.547660
21	1	0	-1.948951	-1.337637	-1.972469
22	1	0	2.315857	0.718532	-1.160297
23	1	0	2.202808	1.338731	0.482838
24	1	0	2.066469	-2.925955	-0.150622
25	1	0	2.292556	-1.887379	-1.553879
26	1	0	2.461290	-0.298825	2.457888
27	1	0	2.550104	-2.043285	2.124408
28	1	0	1.008443	-1.165631	1.970987
29	1	0	4.397694	-0.000589	0.806988
30	1	0	4.394800	-1.746988	0.497406
31	1	0	4.254903	-0.608423	-0.855175
32	16	0	-0.089860	2.028703	-1.505865
33	6	0	0.686228	3.501063	-0.753062
34	1	0	0.357383	3.597615	0.284977
35	1	0	1.779714	3.458581	-0.795094
36	1	0	0.345555	4.363424	-1.336124

##

TP-C=O-TS3

Standard orientation:

Center Number	Atomic Number	Atomic Type	Coordinates (Angstroms) X	Y	Z
1	6	0	2.337640	1.064232	0.964462
2	6	0	2.448158	-0.345278	0.430958
3	16	0	0.429840	-1.287634	1.028646
4	6	0	-0.927409	-0.613621	0.168080
5	6	0	-0.937968	0.588477	-0.495951
6	6	0	0.261122	1.516734	-0.548893

7	6	0	0.963173	1.703313	0.797548
8	8	0	0.476948	2.401456	1.669811
9	6	0	-2.159727	-1.508180	0.188659
10	6	0	-3.497455	-0.759778	0.012052
11	6	0	-3.357857	0.134552	-1.230606
12	6	0	-2.128317	1.043835	-1.215337
13	8	0	-2.169452	2.110409	-1.836229
14	6	0	-3.809780	0.091621	1.259308
15	6	0	-4.631592	-1.778403	-0.194272
16	1	0	3.054564	1.695472	0.420237
17	1	0	2.601620	1.105068	2.024091
18	1	0	2.358848	-0.535495	-0.625418
19	1	0	2.956649	-1.094618	1.014657
20	1	0	0.980687	1.172485	-1.303121
21	1	0	-0.105755	2.495582	-0.872355
22	1	0	-2.052428	-2.250832	-0.617737
23	1	0	-2.168828	-2.081868	1.123126
24	1	0	-4.236808	0.773702	-1.376794
25	1	0	-3.280416	-0.502237	-2.126609
26	1	0	-3.920728	-0.546270	2.145335
27	1	0	-4.747754	0.645640	1.124447
28	1	0	-3.014794	0.814652	1.464409
29	1	0	-4.724967	-2.444846	0.673189
30	1	0	-5.595566	-1.271411	-0.331732
31	1	0	-4.451710	-2.402557	-1.078815
32	6	0	5.065958	-1.675787	-1.295540
33	1	0	4.293367	-2.384634	-0.963636
34	1	0	6.036394	-2.173344	-1.169005
35	1	0	4.916816	-1.506875	-2.370562
36	16	0	5.000483	-0.110669	-0.332719

##

TC-CHD-INT0

Standard orientation:

Center Number	Atomic Number	Atomic Type	Coordinates (Angstroms) X	Y	Z
1	16	0	-0.435709	-2.261254	-0.344666
2	8	0	-1.900917	2.692115	0.442158
3	6	0	-1.057352	0.509278	0.001033
4	6	0	-1.402213	-0.777645	-0.315731
5	6	0	-2.126165	1.542241	0.073004

6	6	0	0.328599	1.031711	0.299666
7	1	0	0.304202	2.119927	0.219803
8	1	0	1.064319	0.695478	-0.481069
9	6	0	0.638516	-0.612044	2.328648
10	1	0	-0.426304	-0.859459	2.249981
11	1	0	0.892927	-0.509173	3.388754
12	6	0	-3.562468	1.162979	-0.287485
13	1	0	-4.049220	2.064241	-0.674544
14	1	0	-4.081603	0.900792	0.648353
15	6	0	-2.831211	-1.176015	-0.670637
16	1	0	-3.337727	-1.551549	0.232801
17	1	0	-2.807905	-2.016919	-1.374519
18	6	0	1.469749	-1.757901	1.718413
19	1	0	1.231552	-2.681674	2.263417
20	1	0	2.535647	-1.550040	1.886453
21	6	0	1.289558	-1.977848	0.212581
22	1	0	1.763152	-2.924213	-0.076254
23	1	0	1.771703	-1.205236	-0.416762
24	6	0	-3.627080	-0.011783	-1.255704
25	6	0	0.923028	0.734003	1.665598
26	8	0	1.671318	1.529126	2.214795
27	1	0	-4.665524	-0.315683	-1.443589
28	1	0	-3.192580	0.276852	-2.221387
29	16	0	2.851405	0.254571	-2.055725
30	6	0	3.913479	1.455041	-1.140260
31	1	0	4.162878	2.324762	-1.765201
32	1	0	3.417640	1.835530	-0.237460
33	1	0	4.863872	1.001267	-0.822916

##

TC-CHD-INT2

Standard orientation:

Center Number	Atomic Number	Atomic Type	Coordinates (Angstroms) X	Y	Z
1	6	0	3.620044	-1.626116	-0.421096
2	6	0	4.012487	-0.485698	0.513370
3	6	0	3.357705	0.821141	0.053941
4	6	0	1.863204	0.658084	-0.136029
5	6	0	1.270402	-0.541867	-0.385702
6	6	0	2.105489	-1.756368	-0.569674

7	16	0	-4.790364	-0.125341	-1.198270
8	6	0	-4.457185	0.503333	0.494292
9	6	0	-2.962568	0.482984	0.887204
10	6	0	-2.391001	-0.953437	0.880055
11	6	0	-0.888399	-0.996337	0.884795
12	6	0	-0.215700	-0.742330	-0.482229
13	8	0	-0.213473	-1.235831	1.877020
14	1	0	4.035609	-1.448458	-1.425923
15	1	0	4.005122	-2.595246	-0.088125
16	1	0	3.822512	1.159436	-0.886414
17	1	0	3.551631	1.607862	0.791526
18	1	0	-4.796732	1.546319	0.594479
19	1	0	-5.017054	-0.072407	1.251162
20	1	0	-2.804984	0.932595	1.882630
21	1	0	-2.420811	1.101406	0.160318
22	1	0	-2.753874	-1.504371	1.756146
23	1	0	-2.796046	-1.413237	-0.037991
24	1	0	-0.415952	-1.634746	-1.087509
25	1	0	-0.717202	0.093894	-0.981963
26	16	0	0.834057	2.095402	0.091261
27	6	0	1.905141	3.472692	-0.461804
28	1	0	1.279516	4.366694	-0.392038
29	1	0	2.782728	3.611839	0.175004
30	1	0	2.216978	3.345634	-1.502160
31	8	0	1.618461	-2.833443	-0.892645
32	1	0	5.103110	-0.367161	0.555087
33	1	0	3.668748	-0.713316	1.530179

###

15. NMR spectrum of products

^{1}H NMR (700 MHz, $CDCl_3$) and ^{13}C NMR (176 MHz, $CDCl_3$) spectra for TP-C=O

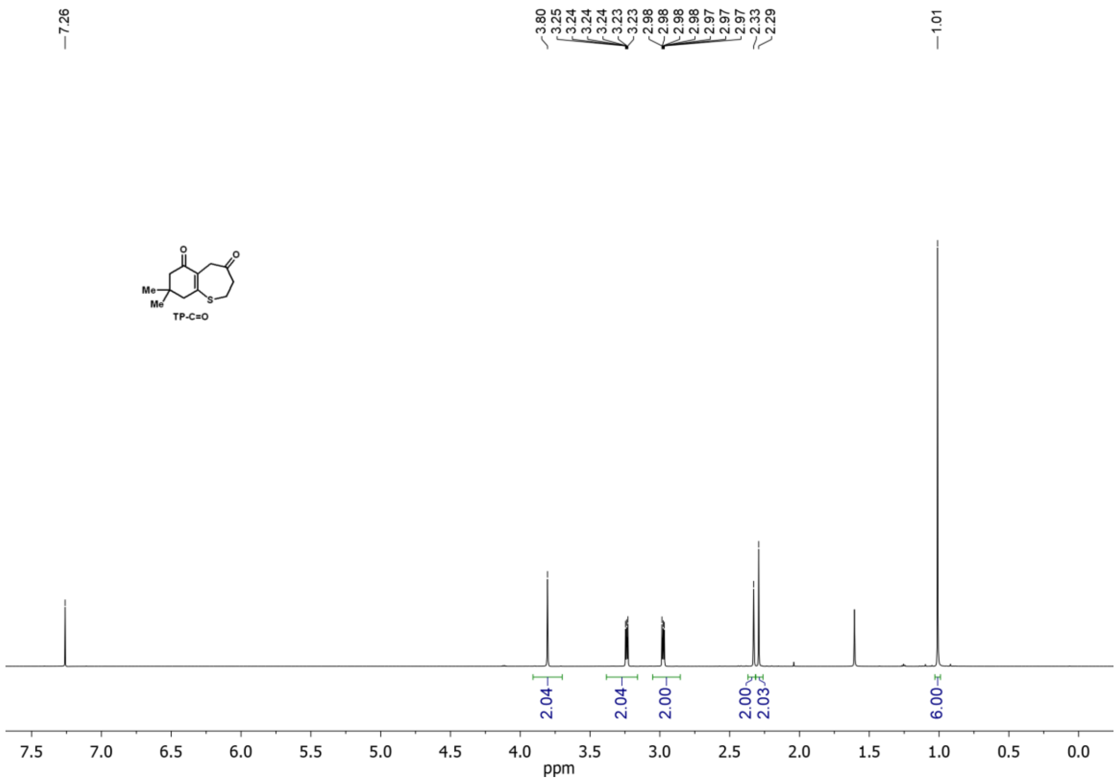

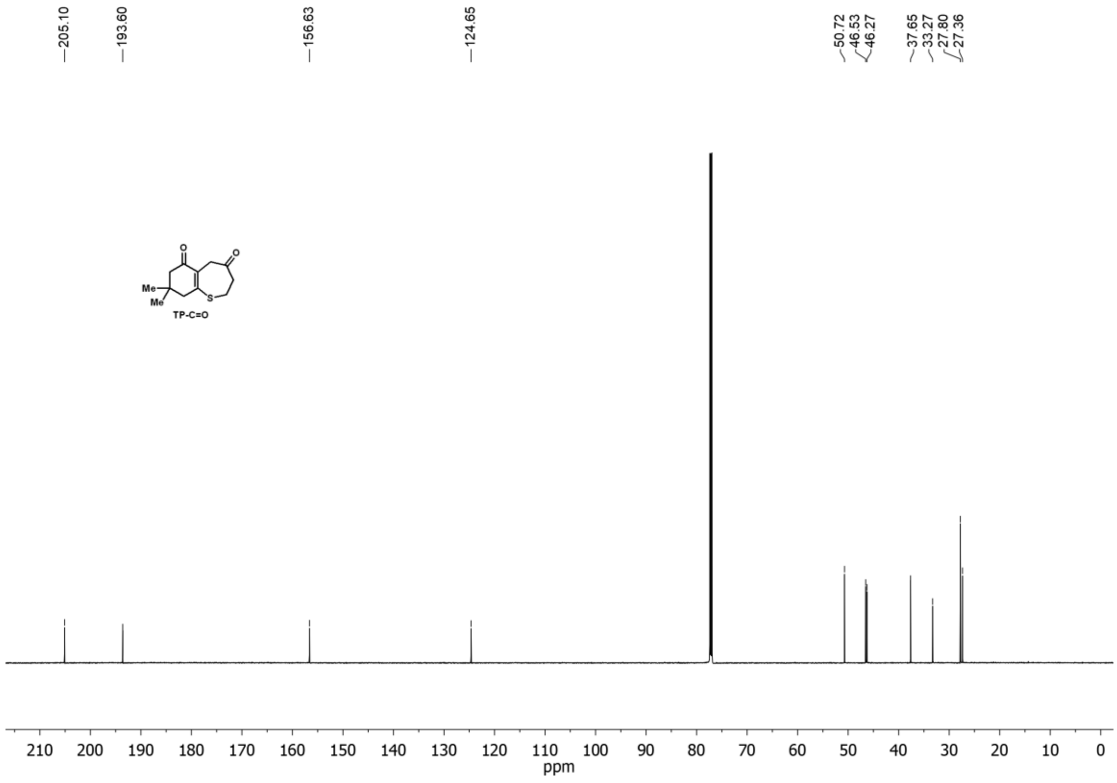

^{1}H NMR (500 MHz, $CDCl_3$) and ^{13}C NMR (176 MHz, $CDCl_3$) spectra for TC-C=O

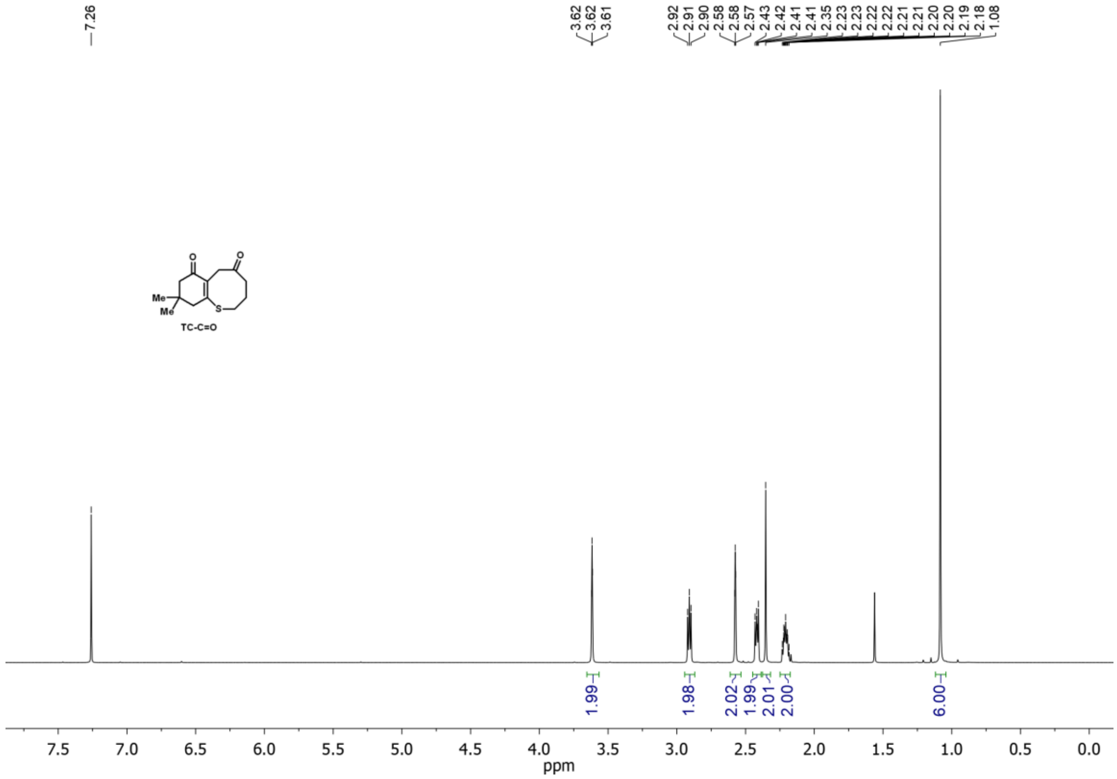

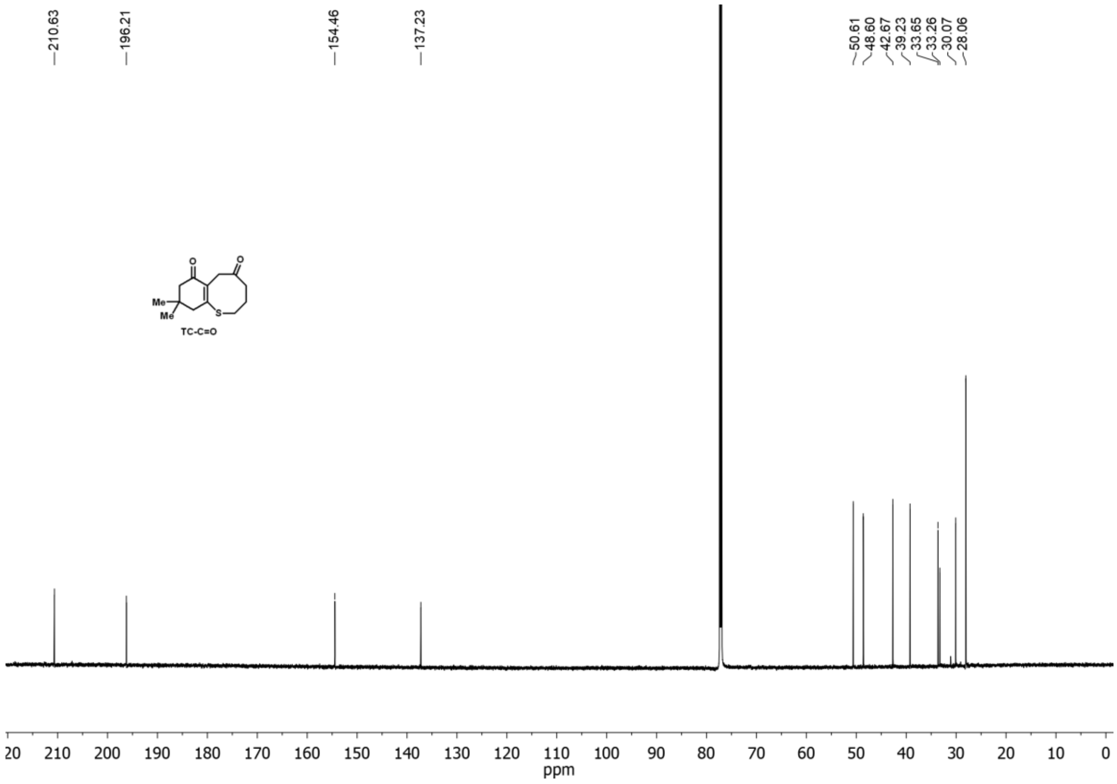

^{1}H NMR (400 MHz, $CDCl_3$) and ^{13}C NMR (176 MHz, $CDCl_3$) spectra for TC-C=C

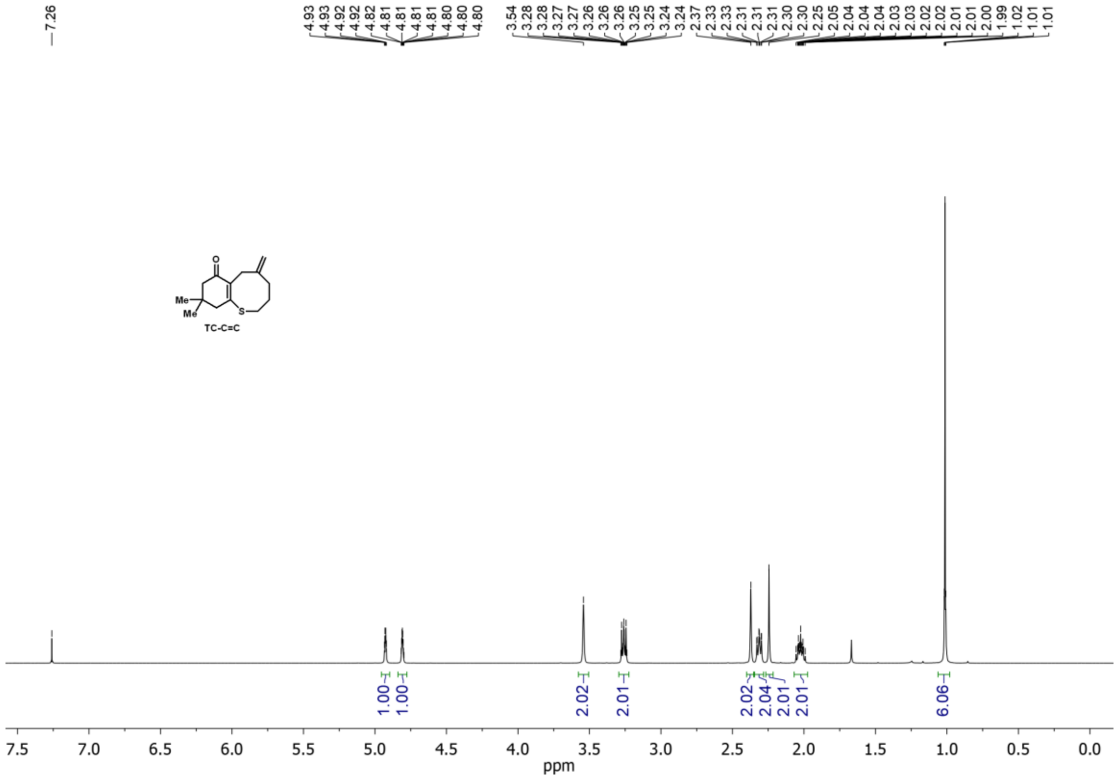

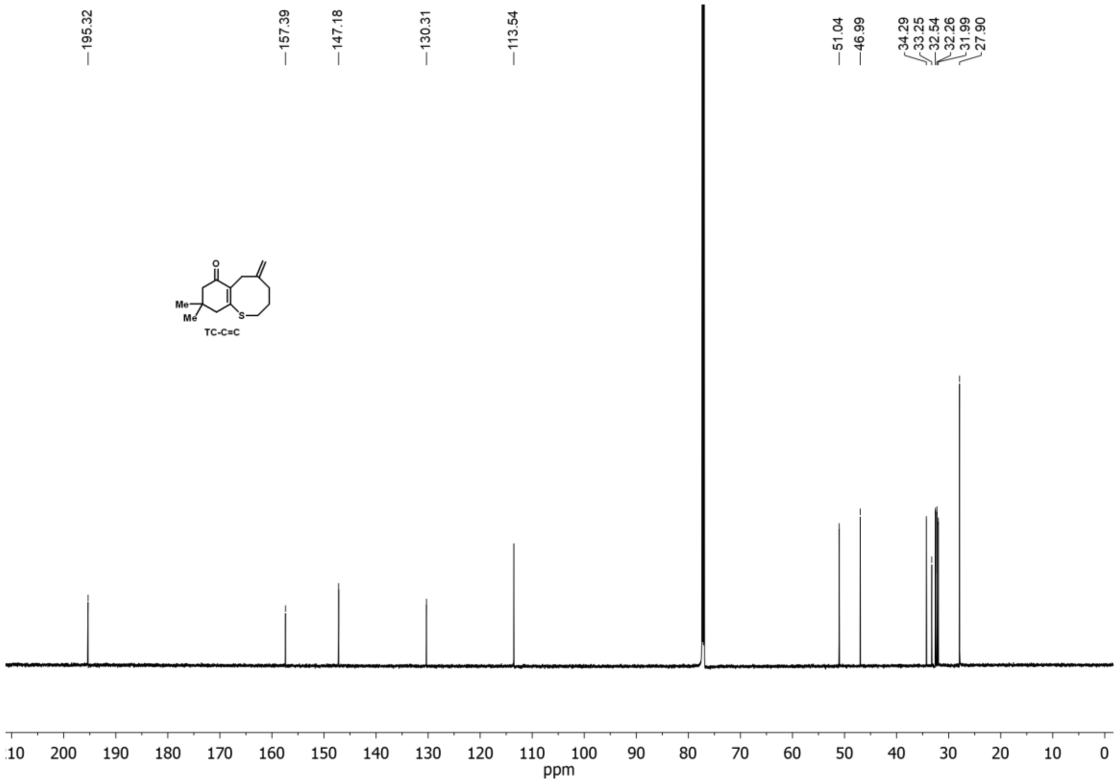

^{1}H NMR (700 MHz, $CDCl_3$) and ^{13}C NMR (176 MHz, $CDCl_3$) spectra for TC-OH

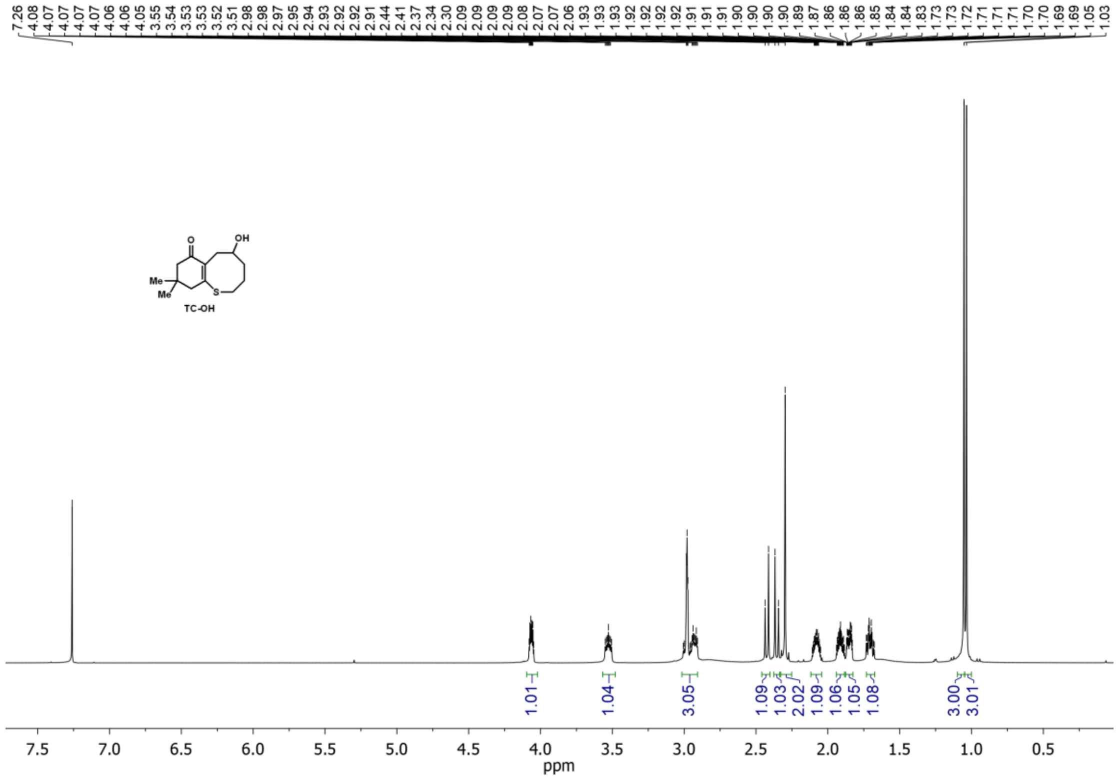

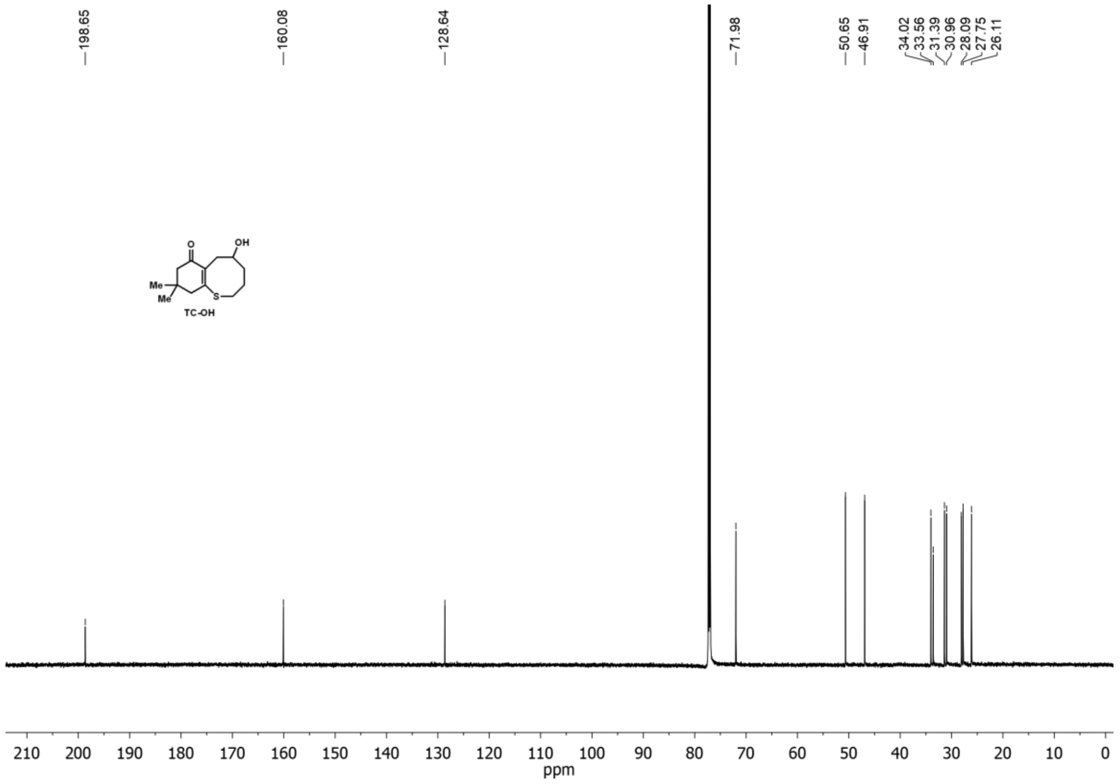

^{1}H NMR (500 MHz, CDCl$_3$) and ^{13}C NMR (176 MHz, CDCl$_3$) spectra for TC-OAc

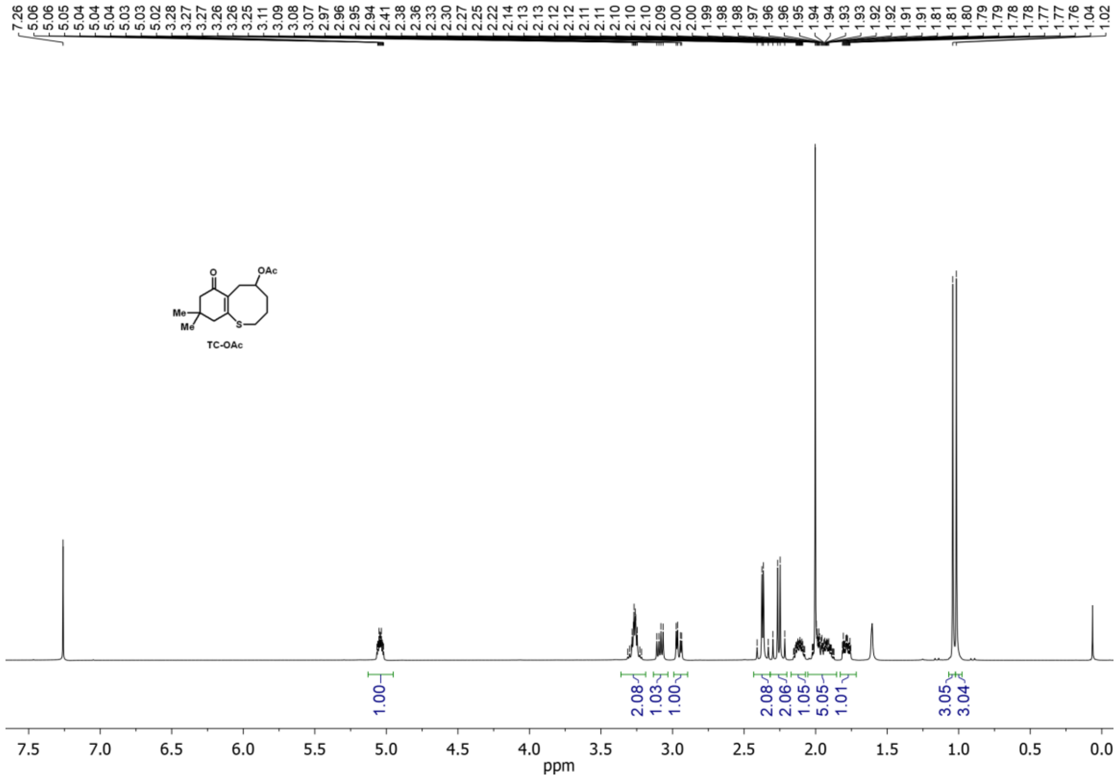

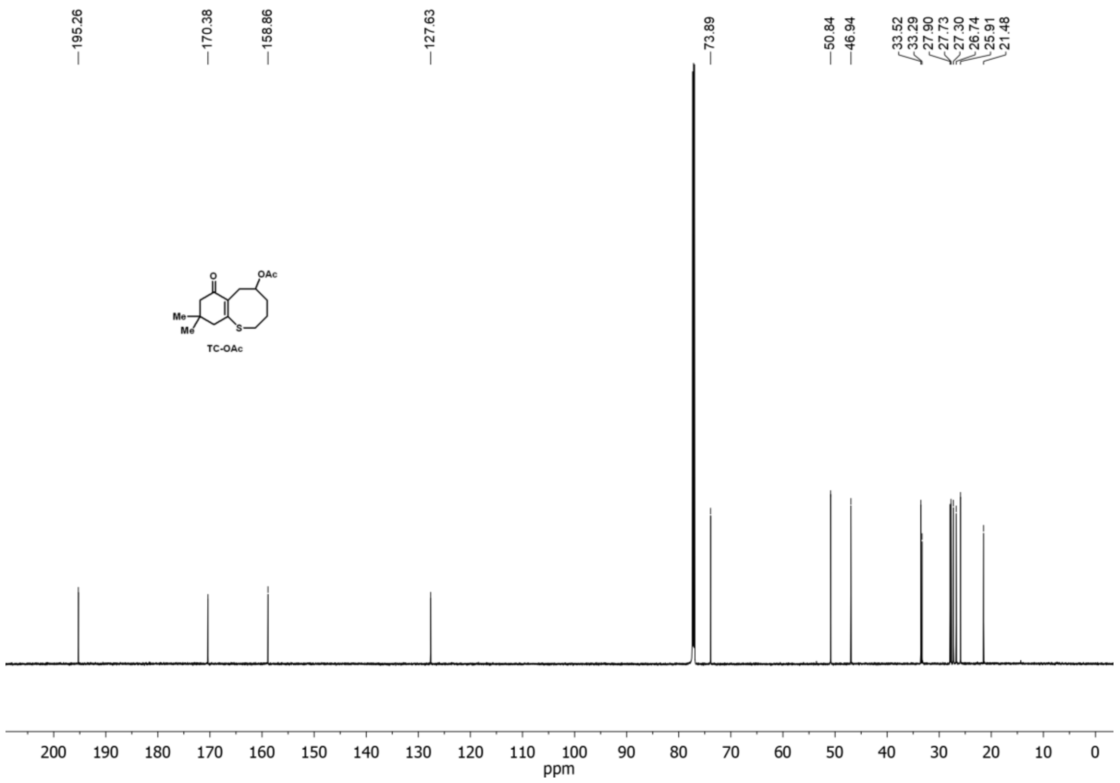

^{1}H NMR (700 MHz, $CDCl_3$) and ^{13}C NMR (176 MHz, $CDCl_3$) spectra for TC-OBz

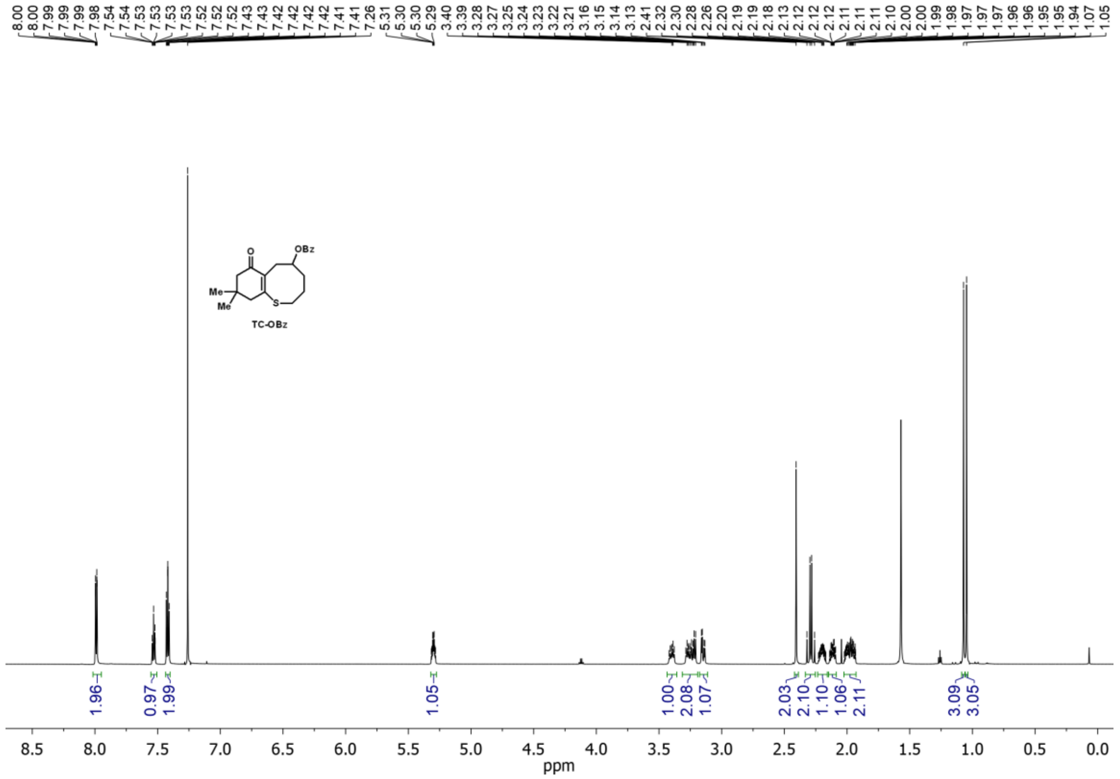

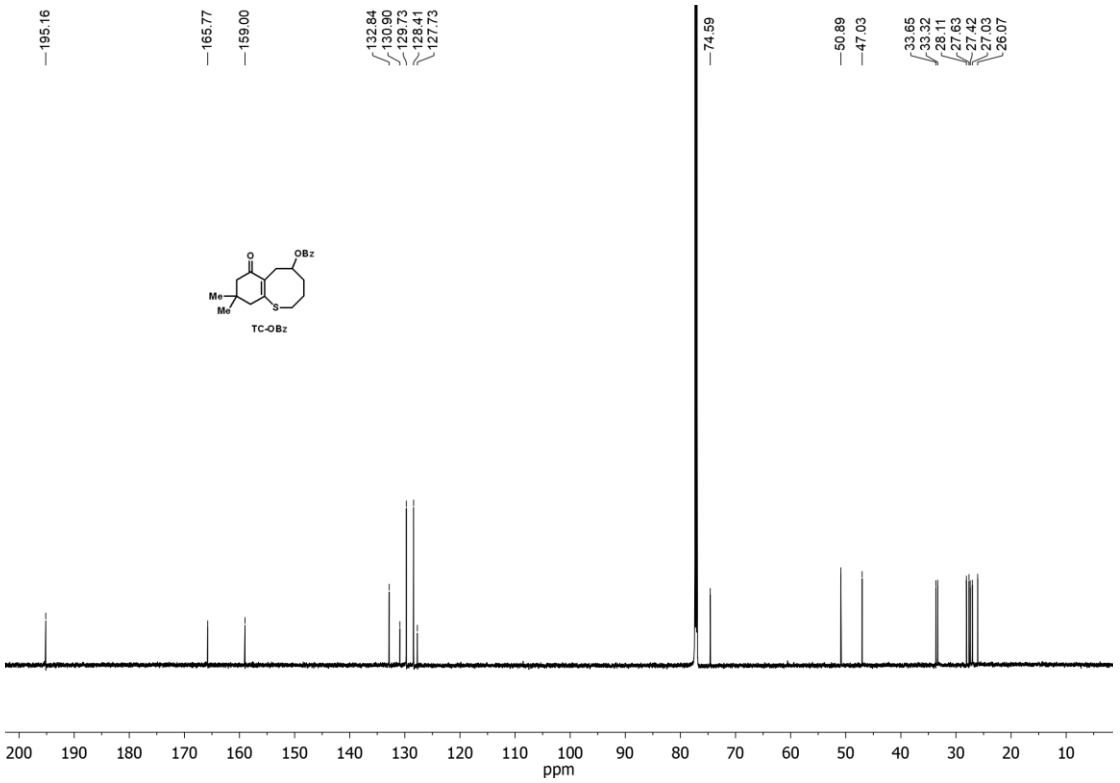

^{1}H NMR (700 MHz, $CDCl_3$) and ^{13}C NMR (176 MHz, $CDCl_3$) spectra for TC-CHD

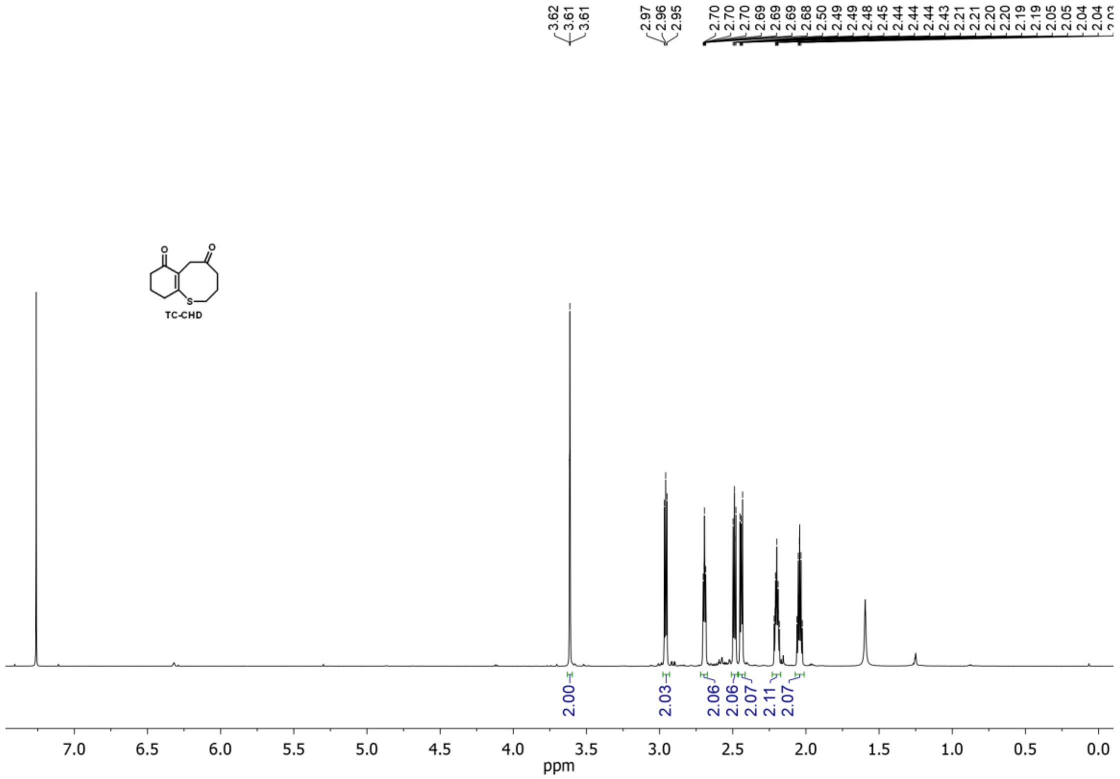

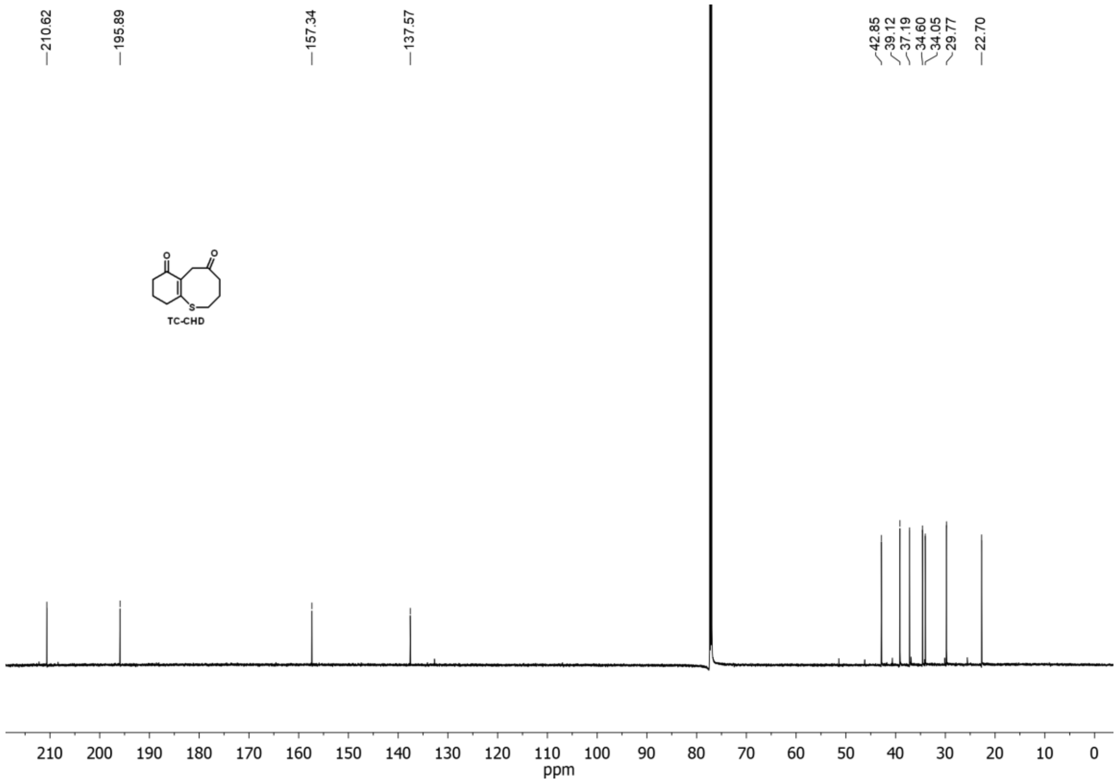

^{1}H NMR (700 MHz, $CDCl_3$) and ^{13}C NMR (176 MHz, $CDCl_3$) spectra for PTC-C=O

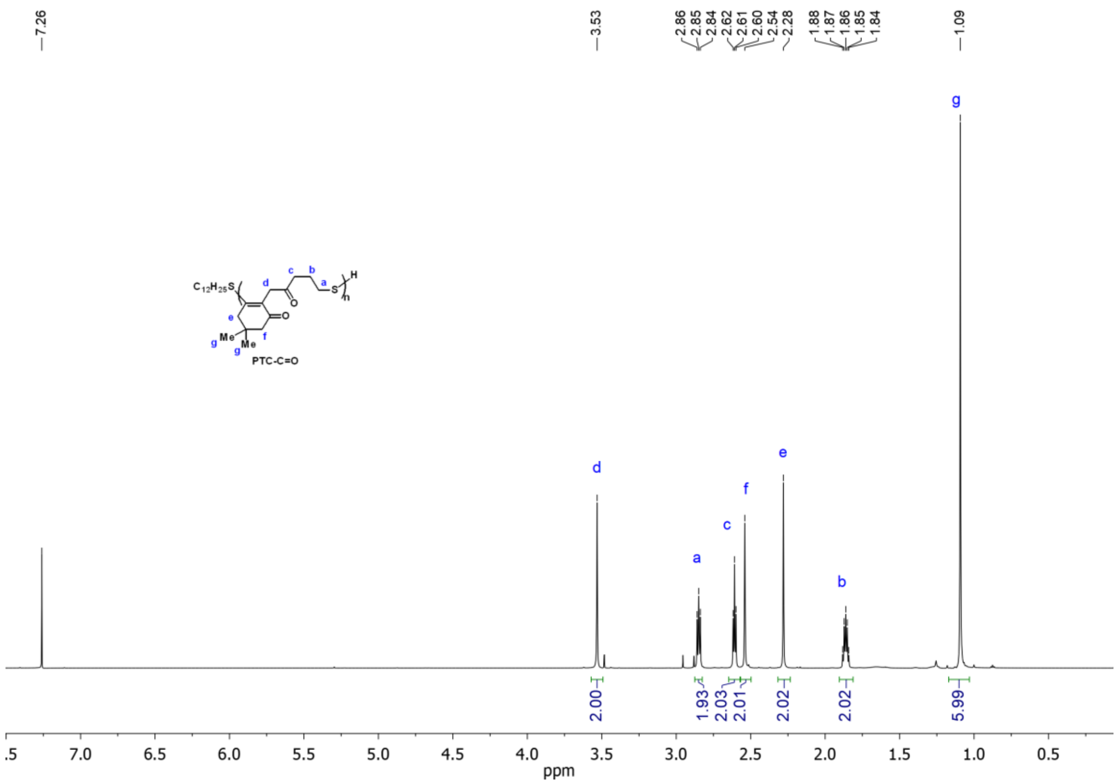

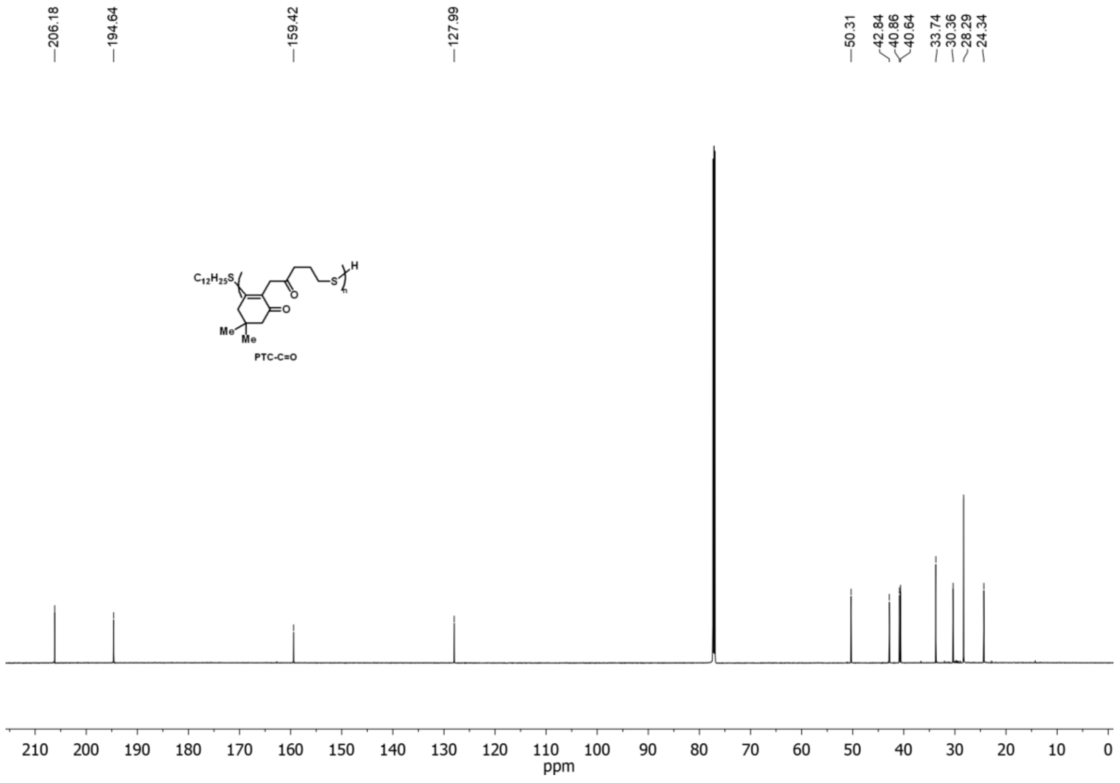

^{1}H NMR (700 MHz, $CDCl_3$) and ^{13}C NMR (176 MHz, $CDCl_3$) spectra for PTC-C=C

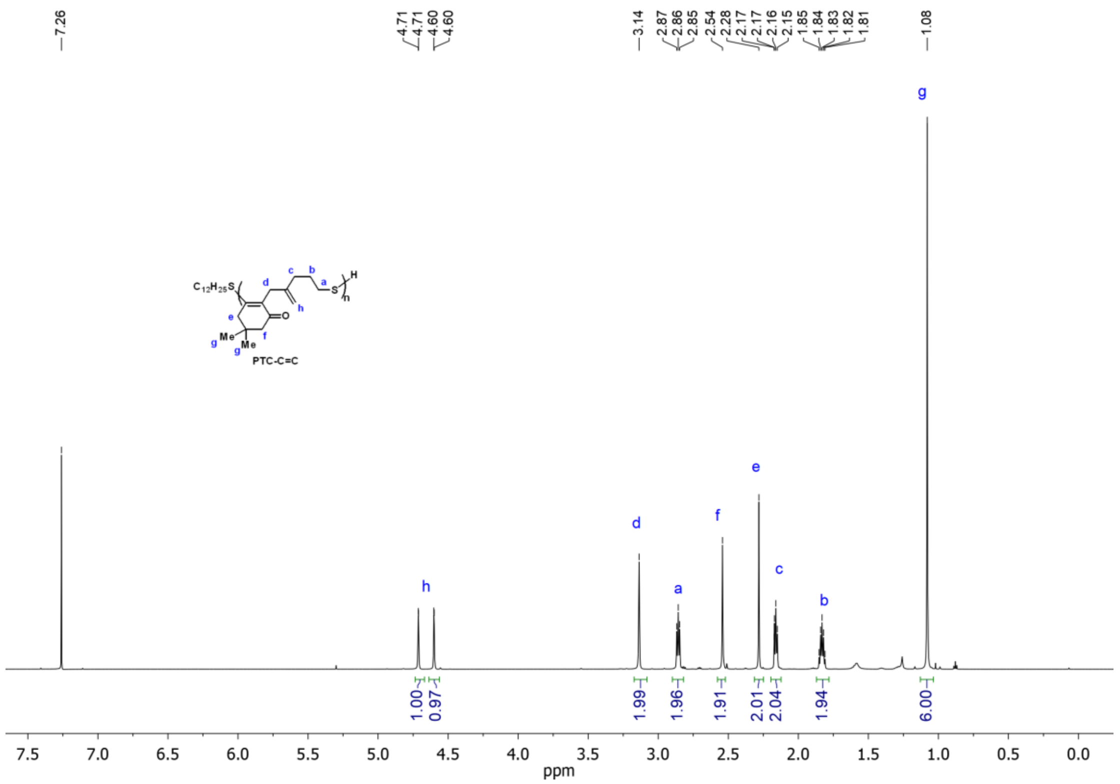

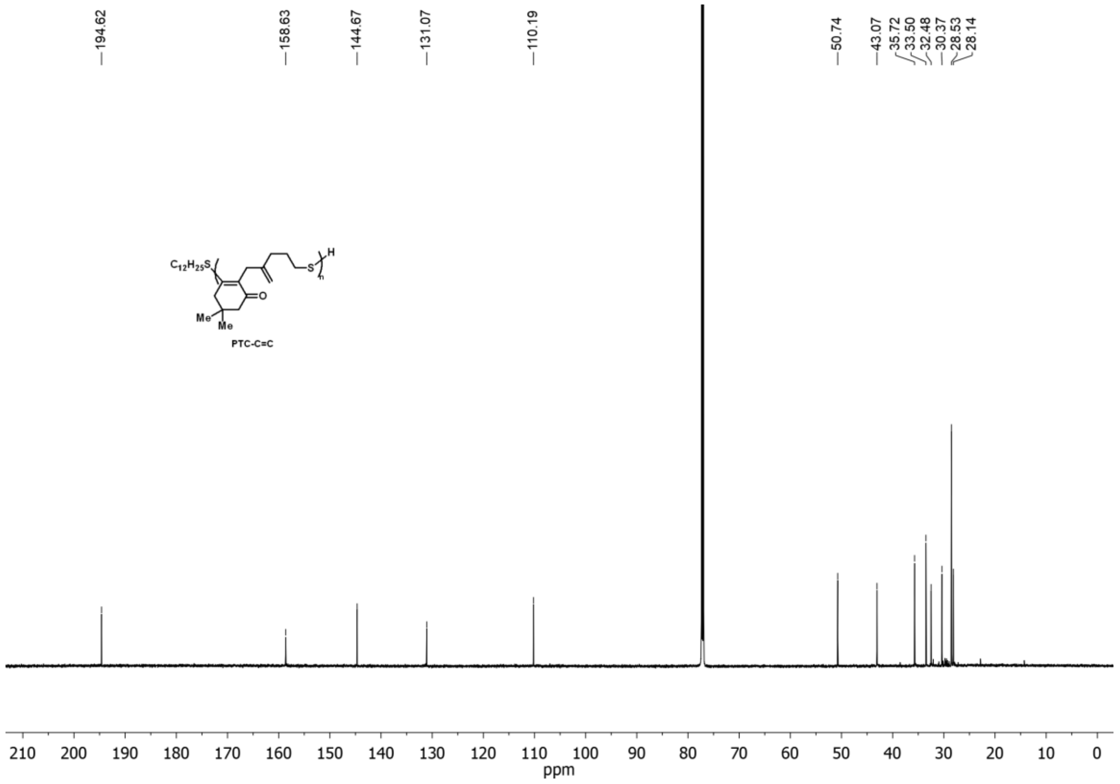

^{1}H NMR (700 MHz, $CDCl_3$) and ^{13}C NMR (176 MHz, $CDCl_3$) spectra for PTC-OAc

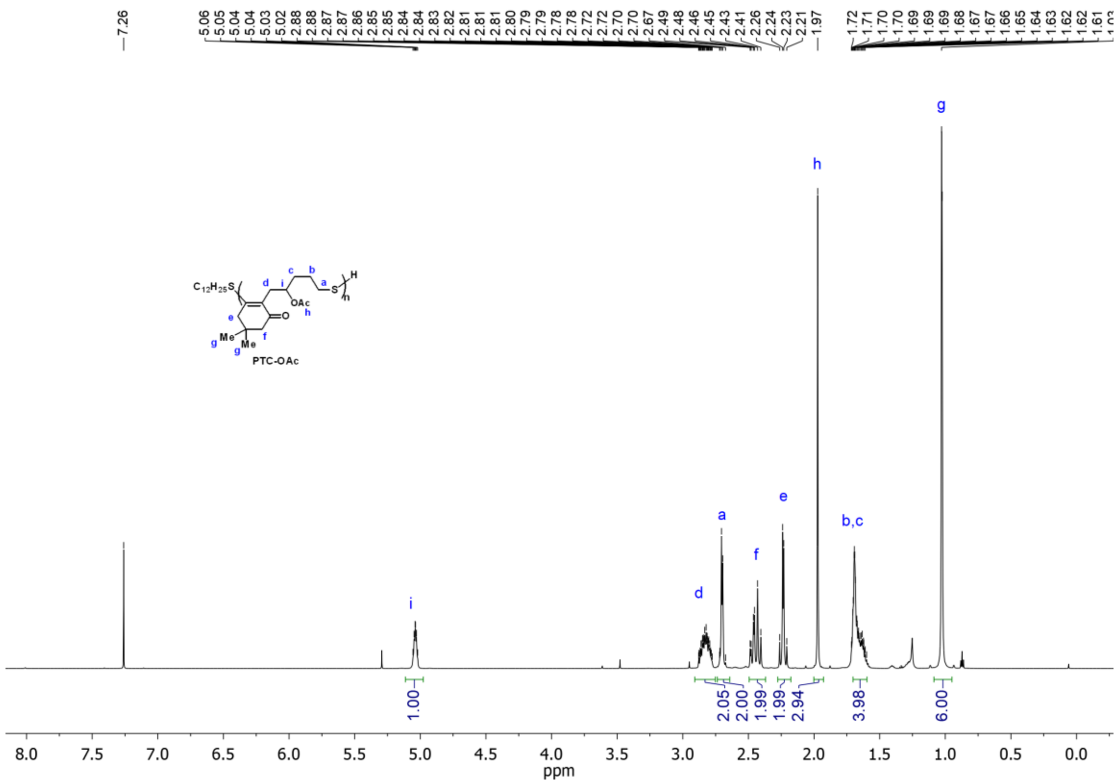

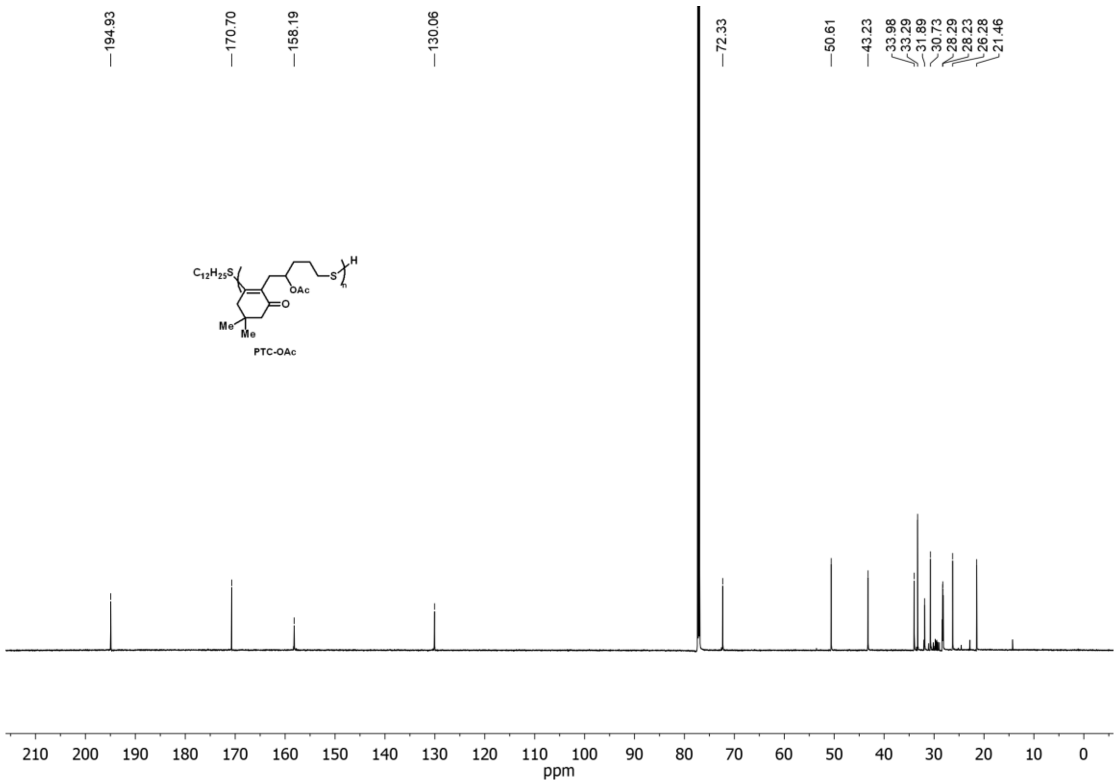

^{1}H NMR (700 MHz, $CDCl_3$) and ^{13}C NMR (176 MHz, $CDCl_3$) spectra for PTC-OBz

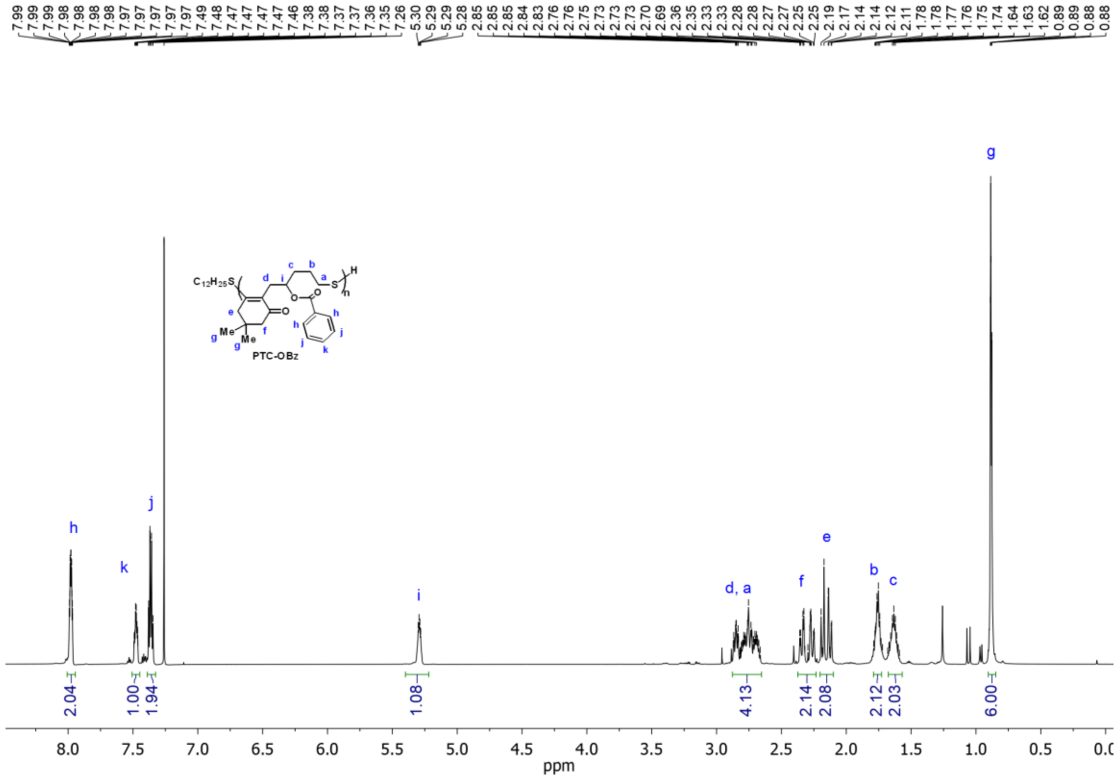

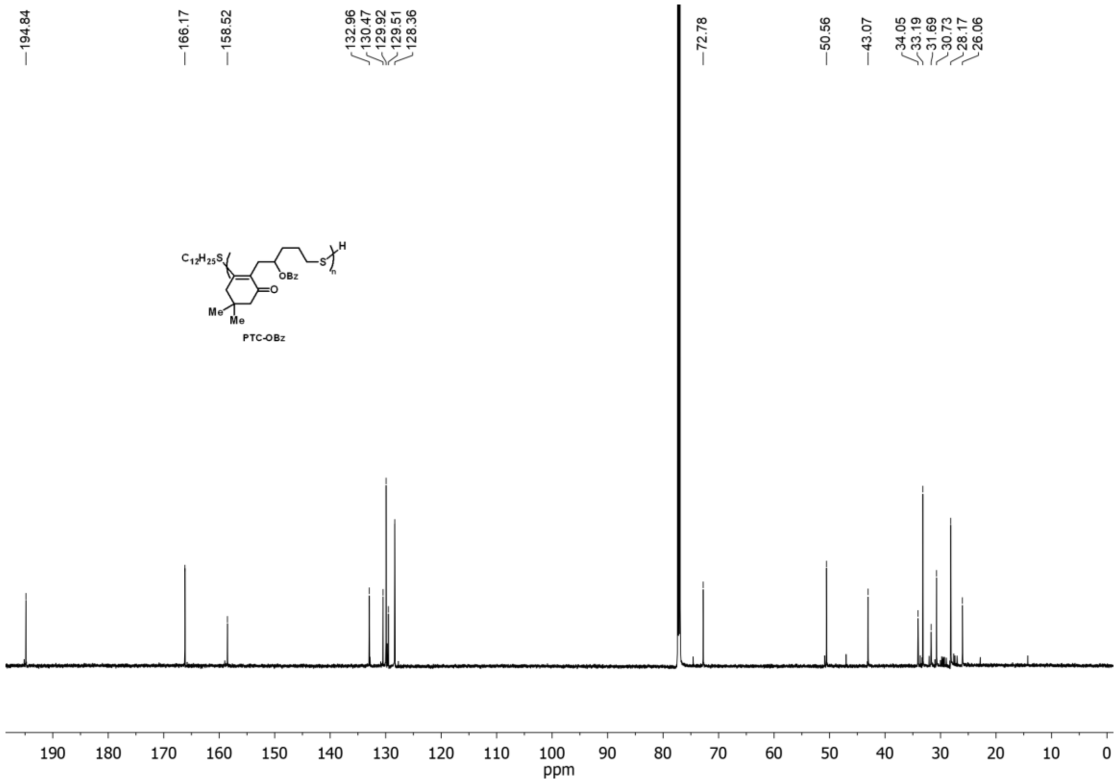

^{1}H NMR (700 MHz, $CDCl_3$) and ^{13}C NMR (176 MHz, $CDCl_3$) spectra for PTC-CHD

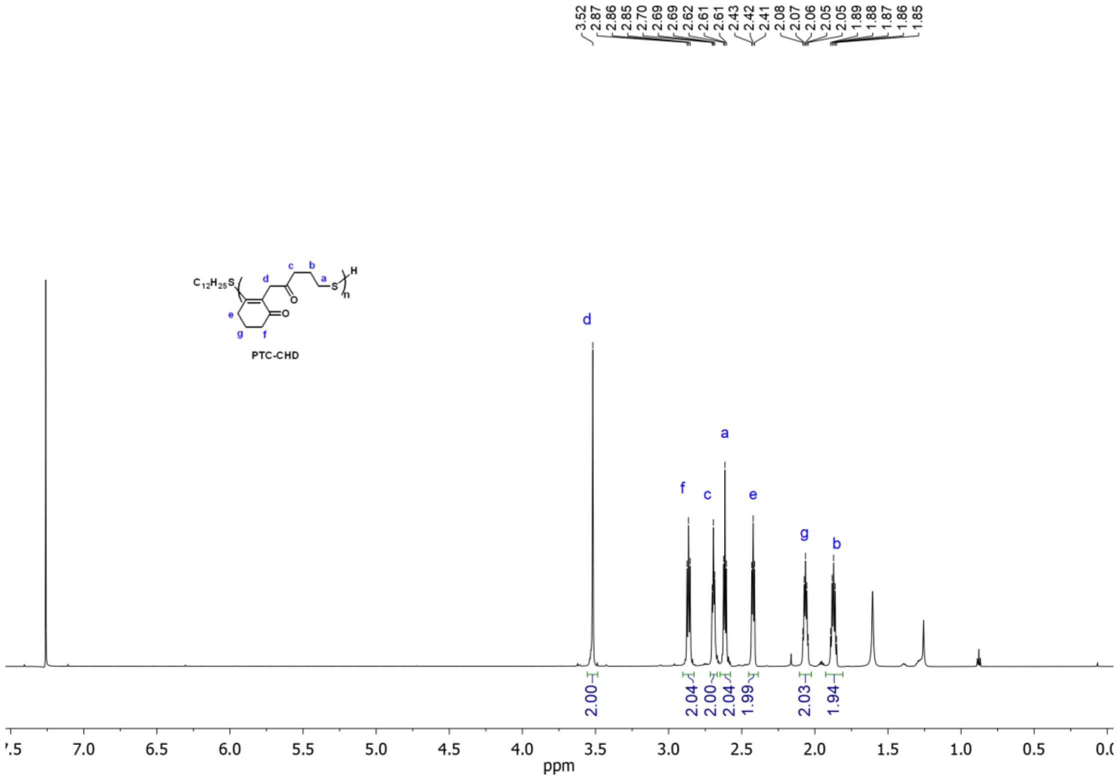

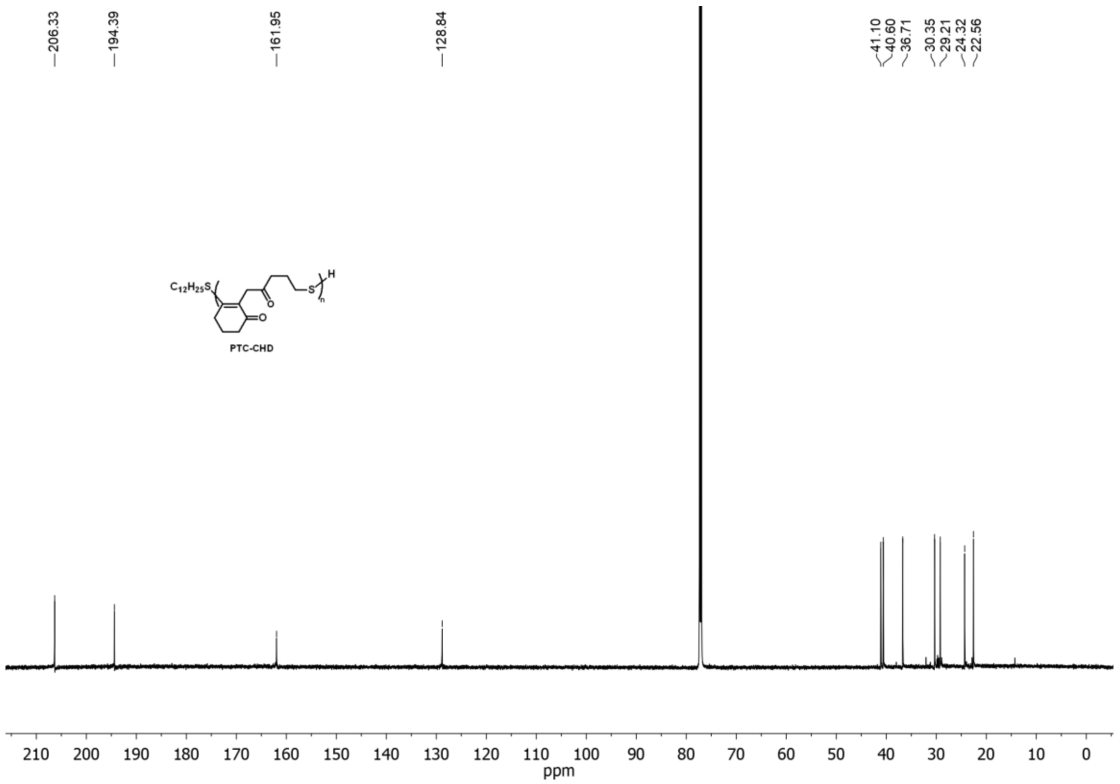

^{1}H NMR (700 MHz, $CDCl_3$) spectra for PTC-C=O-PTC-OAc copolymer

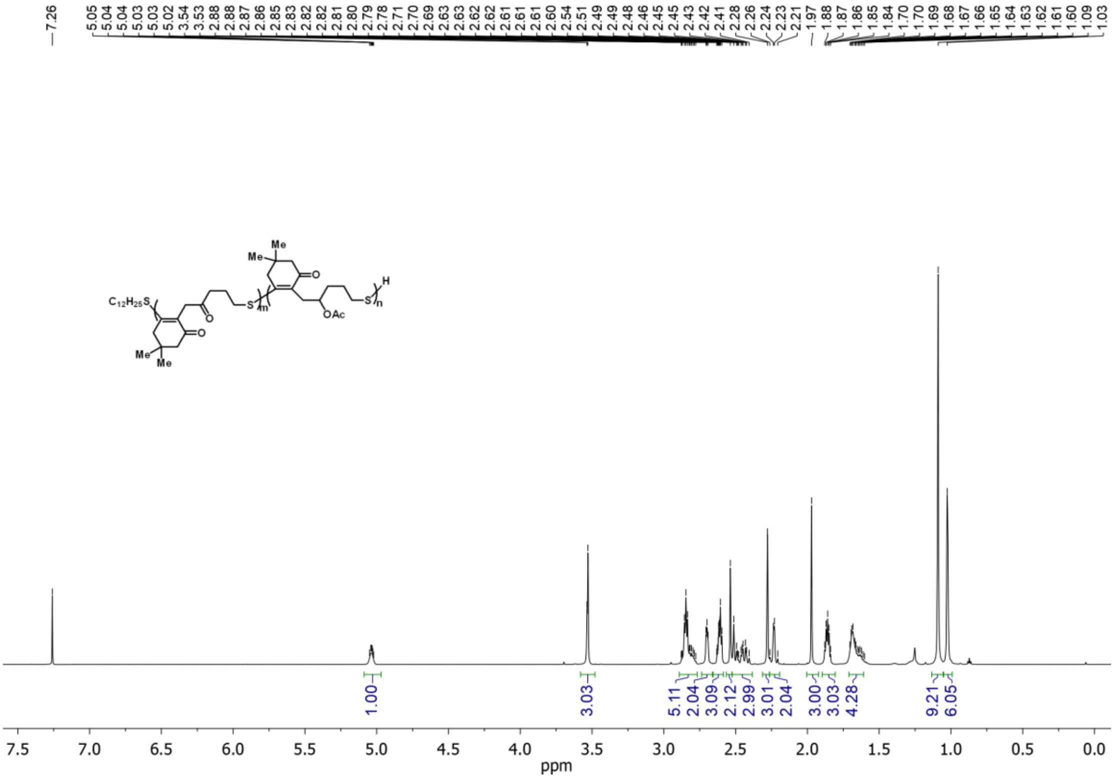

^{1}H NMR (500 MHz, $CDCl_3$) spectra for TP-A

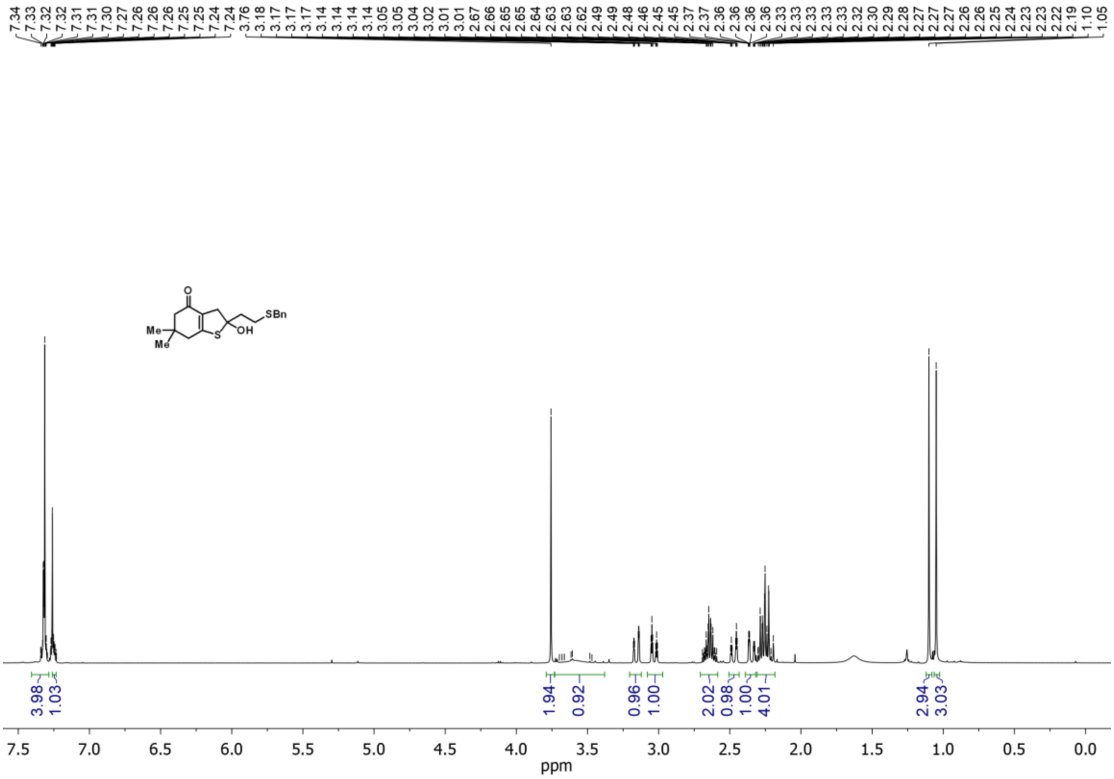

^{1}H NMR (700 MHz, DMSO-d_6) and ^{13}C NMR (176 MHz, DMSO-d_6) spectra for TP-A

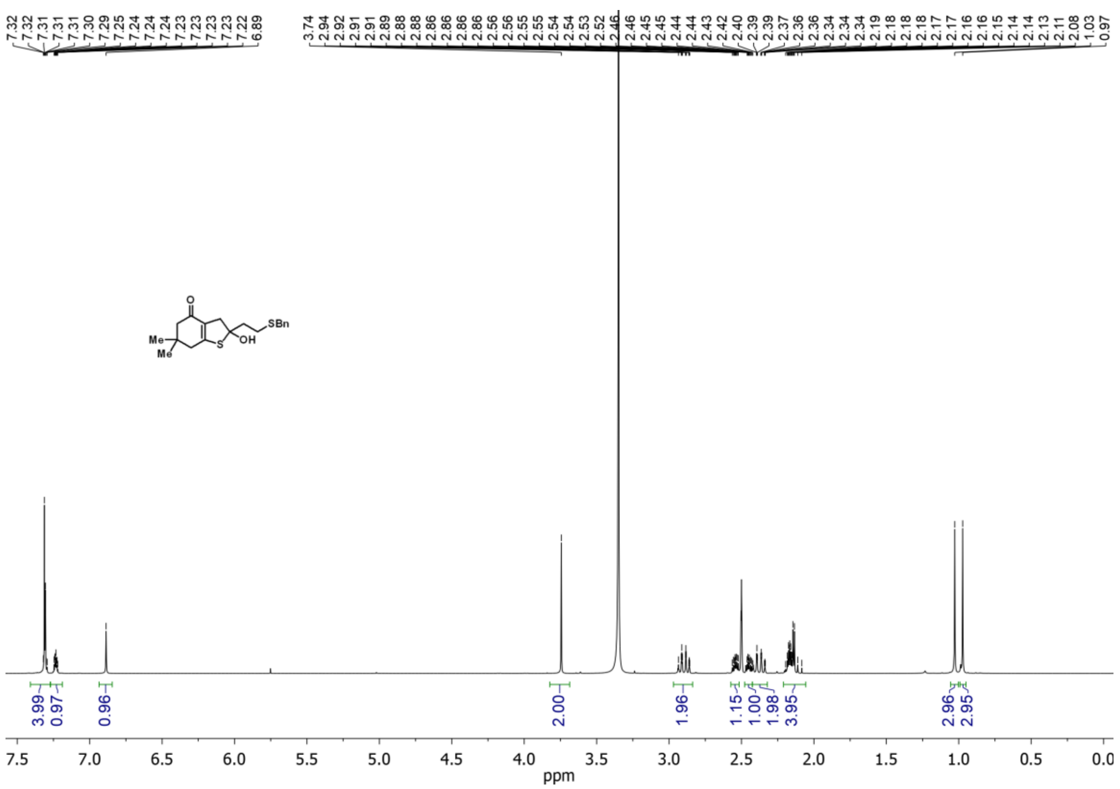

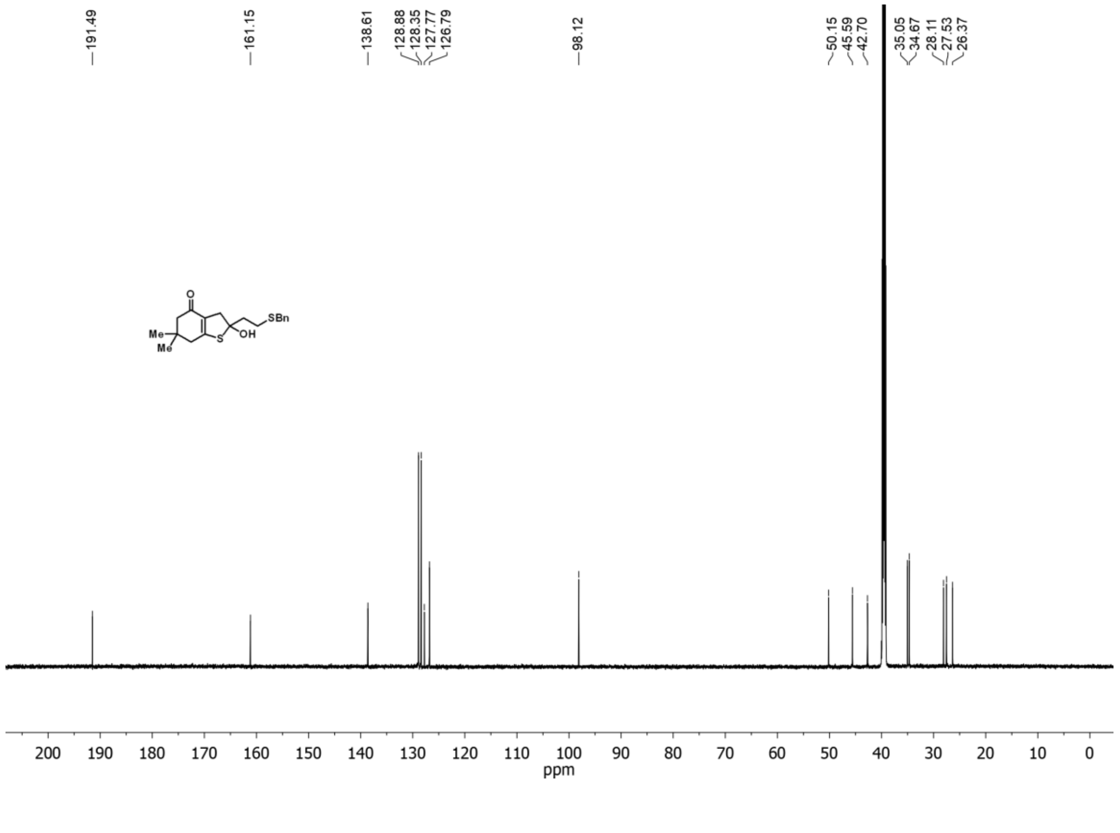

16. References

1. Lu, B.; Li, Y.; Wang, Y.; Aue, D. H.; Luo, Y.; Zhang, L. *J. Am. Chem. Soc.* **2013**, *135*, 8512-8524.

2. Dolomanov, O.V., Bourhis, L.J., Gildea, R.J, Howard, J.A.K. & Puschmann, H. *J. Appl. Cryst*. **2009**, *42*, 339-341.

3. Sheldrick, G.M. *Acta Cryst*. 2**015**, *A71*, 3-8.

4. Sheldrick, G.M. *Acta Cryst*. **2015**, *C71*, 3-8.

5. Frisch, M. J., Trucks, G. W., Schlegel, H. B., Scuseria, G. E., Robb, M. A., Cheeseman, J. R., Scalmani, G., Barone, V., Mennucci, B., Petersson, G. A., Nakatsuji, H., Caricato, M., Li, X., Hratchian, H. P., Izmaylov, A. F., Bloino, J., Zheng, G., Sonnenberg, J. L., Hada, M., Ehara, M., Toyota, K., Fukuda, R., Hasegawa, J., Ishida, M., Nakajima, T., Honda, Y., Kitao, O., Nakai, H., Vreven, T., Montgomery, J. A., Jr., Peralta, J. E., Ogliaro, F., Bearpark, M., Heyd, J. J., Brothers, E., Kudin, K. N., Staroverov, V. N., Kobayashi, R., Normand, J., Raghavachari, K., Rendell, A., Burant, J. C., Iyengar, S. S., Tomasi, J., Cossi, M., Rega, N.,Millam, J. M., Klene, M., Knox, J. E., Cross, J. B., Bakken, V., Adamo, C., Jaramillo, J., Gomperts, R., Stratmann, R. E., Yazyev, O., Austin, A. J., Cammi, R., Pomelli, C., Ochterski, J. W., Martin, R. L., Morokuma, K., Zakrzewski, V. G., Voth, G. A., Salvador, P., Dannenberg, J. J., Dapprich, S., Daniels, A. D., Farkas, O., Foresman, J. B., Ortiz, J. V., Cioslowski, J., and Fox, D. J.; Gaussian 09, revision D.01; Gaussian Inc.: Wallingford, CT, 2013.

6. Becke, A. D., *J. Chem. Phys.* **1993**, 98, 5648−5652.

7. Lee, C.; Yang, W.; Parr, R. G., *Phys. Rev. B*, **1988**, *37*, 785−789.

8. McLean, A. D.; Chandler, G. S., *J. Chem. Phys.* **1980**, *72*, 5639–5648.

9. Zhao, Y.; Truhlar, D. G., *Theor. Chem. Acc.* **2008**, *120*, 215-241.

10. Weigend F.; Ahlrichs R., *Phys. Chem. Chem. Phys.* **2005**, *7*, 3297–3305.
